# Reference standards to assess physical fitness of children and adolescents of Brazil: an approach to the students of the Lake Itaipú region—Brazil

**DOI:** 10.7717/peerj.4032

**Published:** 2017-11-30

**Authors:** Edilson Hobold, Vitor Pires-Lopes, Rossana Gómez-Campos, Miguel de Arruda, Cynthia Lee Andruske, Jaime Pacheco-Carrillo, Marco Antonio Cossio-Bolaños

**Affiliations:** 1 Universidade Estadual do Oeste do Paraná, Parana, Brazil; 2 Research Center in Sports Sciences, Health Sciences and Human Development (CIDESD) and Department of Sports Science of Polytechnic Institute of Bragança, Braganca, Portugal; 3 Universidad Autonoma de Chile, Talca, Chile; 4 Faculty of Physical Education, State University of Campinas, Campinas, Brazil; 5 Faculty of Educational Sciences, Universidad de Talca, Linares, Chile; 6 Universidad del Bio Bio, Chillan, Chile; 7 Universidad Católica del Maule, Talca, Chile; 8 Instituto del Deporte Universitario, IDUNSA, Universidad Nacional de San Agustín, Arequipa, Arequipa, Perú

**Keywords:** Reference, Children, Adolescents, Physical fitness

## Abstract

**Background:**

The importance of assessing body fat variables and physical fitness tests plays an important role in monitoring the level of activity and physical fitness of the general population. The objective of this study was to develop reference norms to evaluate the physical fitness aptitudes of children and adolescents based on age and sex from the lake region of Itaipú, Brazil.

**Methods:**

A descriptive cross-sectional study was carried out with 5,962 students (2,938 males and 3,024 females) with an age range of 6.0 and 17.9 years. Weight (kg), height (cm), and triceps (mm), and sub-scapular skinfolds (mm) were measured. Body Mass Index (BMI kg/m^2^) was calculated. To evaluate the four physical fitness aptitude dimensions (morphological, muscular strength, flexibility, and cardio-respiratory), the following physical education tests were given to the students: sit-and-reach (cm), push-ups (rep), standing long jump (cm), and 20-m shuttle run (m).

**Results and Discussion:**

Females showed greater flexibility in the sit-and-reach test and greater body fat than the males. No differences were found in BMI. Percentiles were created for the four components for the physical fitness aptitudes, BMI, and skinfolds by using the LMS method based on age and sex. The proposed reference values may be used for detecting talents and promoting health in children and adolescents.

## Introduction

Physical fitness is a strong indicator of health in childhood years as well as in adulthood (Blaes et al., 2011; Ortega et al., 2008). It is associated with the decrease in the risk of cardiovascular diseases (Soares-Miranda et al., 2015) and the development of a healthy body weight (Lu et al., 2014), among other aspects.

In general, physical fitness tests within the school educational system are an important tool to measure the achievements of the learning standards associated with physical education (Tremblay & Lloyd, 2010a). These standards are set by the results of field physical tests (Golle et al., 2015). These are commonly used by international schemes to assess the levels of physical wellbeing of children and adolescents in schools (Catley & Tomkinson, 2013; De Miguel-Etayo et al., 2014; Meredith, Welk & the Cooper Institute, 2007; Ruiz et al., 2011; Tremblay et al., 2010b).

In Brazil, reference standards to evaluate physical fitness associated with health are currently missing. These standards are necessary to rank children and to monitor physical fitness of the school community (De Miguel-Etayo et al., 2014).

Currently, the test models used nationally and internationally allow us to analyze the results using the morphological, muscular strenght, flexibility, and cardiovascular factors. Nonetheless, to our knowledge, no recent studies exist that are capable of assessing the wide range of factors associated with the physical fitness of Brazilian children and adolescents. This is due to the fact that it is generally known that today’s children and adolescents have a variety of paces and patterns of physical development throughout the world. In addition, the multiple protocols developed by countries to measure the components of physical aptitude and the indicator values for human development are not adequate for comparing the references for levels of physical aptitude between regions. This forces the countries and their administrative regions to establish their own models to measure the general wellbeing of their populations, with all physical fitness factors included.

Consequently, the regulatory data from field tests represents the possibility for analyzing and studying health promotion and sports skills. Therefore, this would provide objective recommendations for assessing physical fitness during physical education classes (Golle et al., 2015). Furthermore, in the past few years, the increasing number of publications about obesity, cardiovascular diseases, and metabolic problems (American Diabetes Association, 2000) show the emergence and the importance of these health problems that today’s society faces.

In that context, studying physical fitness using its four factors and the chronological age as starting points could provide a way towards achieving relevant data and reaching a common references to describe physical fitness patterns of students of the Itaipú (Brazil). This is particularly feasible since physical education classes are held for 50 min two to three times a week.

Thus, the goal of this study was to develop reference standards that allow us to assess the physical fitness factors of children and adolescents based on age and gender in the Lake Itaipú region-Brazil.

**Figure 1:**
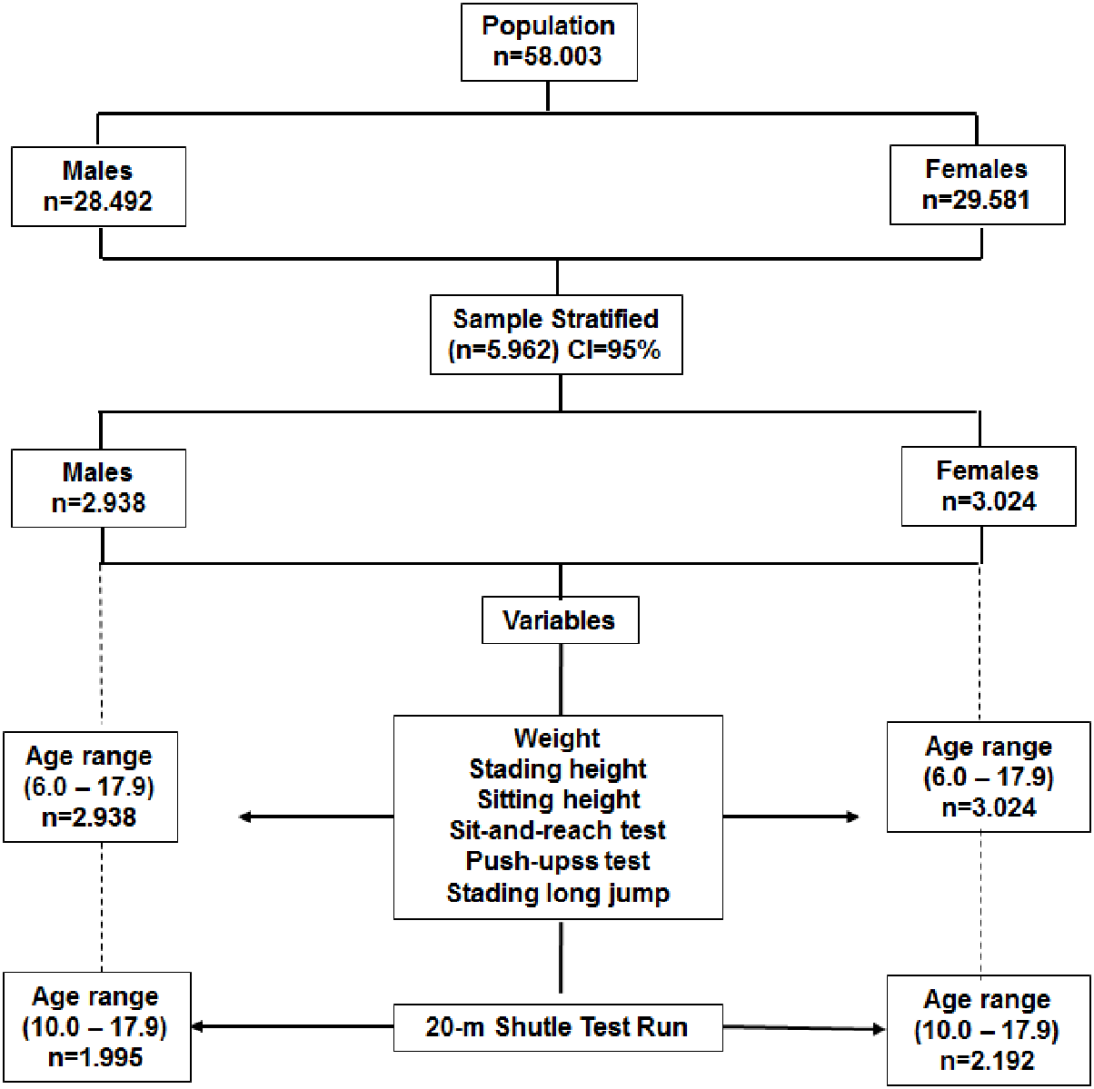
Flowchart outlined to select the study sample.

## Materials

The researcher team designed a cross-sectional descriptive study that included 5,962 students (2,938 men and 3,024 females) from the Lake Itaipú region in Paraná (Brazil). The age range ranged from 6.0 to 17.9 years old. Sample selection was probabilistic (stratified). Age and sex was used to randomly select the sample from the total available population of students. The sample selection process is illustrated in the flowchart of Fig. 1. The educational system for the state of Paraná (Brazil) provides physical education for elementary education in the development of sports, games, gymnastics, boxing, and dance (DCEBEF, 2008). Physical education classes are 50 min a day three times a week for elementary (6–14 years old) and two times a week for high school students (15–18 years old). The study was conducted in accordance with the guidelines established by the Ethics Committee of the School of Medical Sciences of the University of Campinas (São Paulo, Brazil)- 2012-785.

There were 34 public education schools (elementary and high school education) included in the study. These schools are located in 11 counties that are part of the Lake Itaipú region in the state of Paraná (Brazil). This region is located in the west of Paraná, 627 km from the capital of Curitiba. It is bordered and contained by the neighboring country of Paraguay. According to the United Nations Program (PNUD, 2010), the Human Development Index (HDI) of this region in 2010 was between 0.700 and 0.799.

Students with physical or mental limitations and subjects with a Body Mass Index (IBMI) greater than the p97 percentile based on cut off points from the Centers for Disease Control and Prevention were not included in the study (Kuczmarski et al., 2000). Written informed consent was obtained from the parents or guardians and students. The study was approved by the ethical committee of the Medical School of the State University of Campinas SP (Brazil).

## Methods

The anthropometric evaluation and the physical fitness testing were conducted from April to November 2012. The data collection was done from Monday to Friday during mornings and afternoons (8 am to 12 pm and 1 pm to 5 pm) inside the facilities of the respective schools.

### Anthropometric measures

All of the anthropometric measurements were carried out by five experienced anthropometrists. The technical measurement error was below 1.5%. A standard anthropometric measurement protocol was adopted as described by Ross & Marfell-Jones (1991).

Body weight and height were measured with the students in bare feet and with as few clothes on as possible. A digital scale (model BC601; Tanita, Manchester, UK) and a portable stadiometer (model 217; Seca Gmbh & Co. KG, Hamburg, Germany) were used to take all respective measurements. Body Mass Index (BMI) was calculated using the standard equation: weight (kg)/height squared (m^2^). Triceps and subscapular skinfolds were measured with a Harpenden skinfold caliper (Harpenden, England).

**Figure 2:**
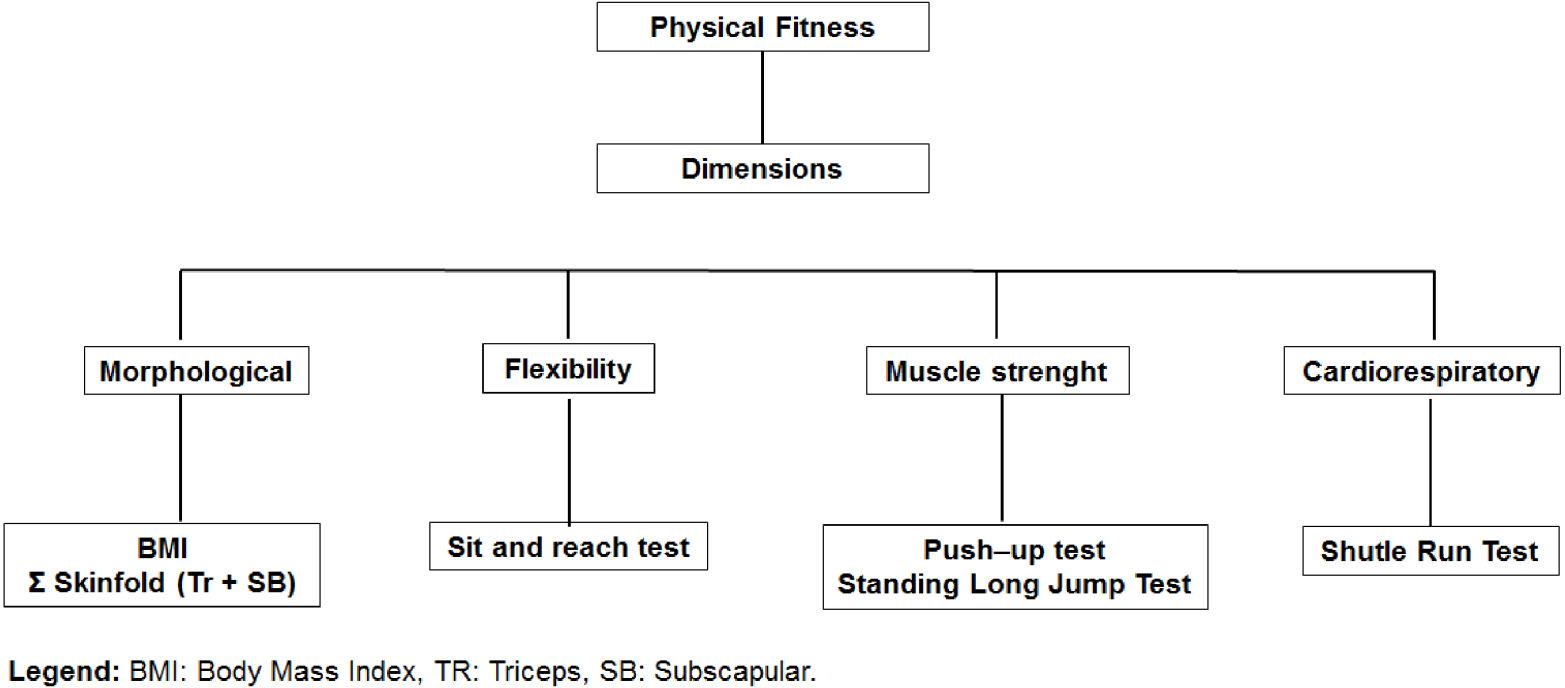
Organization of physical fitness dimensions: BMI, Body Mass Index; TR, Triceps; SE, Subscapular.

### Physical fitness

The test battery was designed from four dimensions of physical fitness (morphological, muscular strength, flexibility, and cardio-respiratory). Figure 2 illustrates the organization of the tests for each dimension.

In all of the cases, the highest points obtained were interpreted as indicators of better physical performance. During the physical tests carried out in each of the schools, a nurse was always present. The nurse was to assist in case of an emergency during the physical tests. All of the tests, except for the 20-m Shuttle Run Test, were conducted twice (*n* = 596). The purpose was to verify the Technical Error of Measurement (TEM) of 10% of the sample in the research study. The values varied from 0.8 to 2.7%.

Physical fitness evaluation included the following test items:

(1) Sit-and-reach to measure flexibility of the hamstrings muscles and lower back. The procedures followed the YMCA of the USA (2000) suggestions. Three trials were conducted, and the best performance was recorded.

(2) Push-ups test to measure muscle endurance. For girls, the push-up test was modified by resting on the knees (as opposed to toes). The number of push-ups was registered (Canadian Association of Sport Sciences, 1986).

(3) Standing long jump to measure power muscular fitness of leg muscles. Three trials were carried out, and the best performance was recorded (Castro-Piñero et al., 2010).

(4) A 20-m shuttle run to measure aerobic fitness (Leger et al., 1998). The running pace was imposed by a sound signal. The initial speed was 8.5 km/h-1, and it increased in 0.5 km/h in 1 min intervals. The testing finished when the student stopped, due to fatigue, or when he/she did not reach the line at the same time as the audio signal, on two consecutive occasions. This test was performed only once. A group of 6–8 students were tested simultaneously.

The evaluation of physical testing was performed in the facilities of each school. Previously, a warm up of about 10 to 15 min was carried out to familiarize the students with the procedures. Afterwards, the tests were carried out in the following order: sit-and-reach, push-ups, standing long jump and Shuttle Run test.

### Statistics

The normal distribution of the data was verified by using the Kolmogorov–Smirnov Test. Descriptive statistics of the arithmetic average, standard deviation, frequencies, and percentages. The differences between both sexes were determined by a *t*-test of independent samples. Smoothed percentile curves were created for physical fitness for each sex based on the LMS method (Cole et al., 2000). LMS Chart Maker Pro Version 2.3 software (Pan, Cole & Chartmaker, 2006) was used. The final percentile curves were the result of smoothing three age-specific curves: L (lambda; skewness), M (Mu; median), and S (sigma; coefficient of variation). P10, P50, and P85 to BMI and to the sum of skinfolds. P15, P50, and P85 to the physical tests.

## Results

Table 1 shows the descriptive statistics (mean and standard-deviation) for all variables measured.

**Table 1:** Descriptive statistics (mean ± standard-deviation) for somatic variables and physical fitness ítems for males and females. And results for difference between males and females.

	Males (*n* = 2,938) X ± SD	Females (*n* = 3,024) X ± SD	*p*
Age (years)	11.7 ± 3.2	12.1 ± 3.2	
***Somatic measures***			
Weight (kg)	45.4 ± 17.8	43.9 ± 14.7	^***^
Height (cm)	151.1 ± 18.8	149.1 ± 15.1	^***^
Sitting height (cm)	77.9 ± 9.3	78.3 ± 8.1	
BMI (kg/m^2^)	19.1 ± 4.0	19.2 ± 4.0	
Σ skinfolds (TR+SB)	20.5 ± 12.4	26.8 ± 12.9	^***^
***Physical fitness***			
Sit-and-reach (cm)	24.2 ± 7.0	25.8 ± 7.0	^***^
Push-ups (# Reps)	11.3 ± 8.3	9.8 ± 7.1	^***^
Standing long jump (cm)	142.2 ± 33.8	113.9 ± 23.0	^***^
20-m Shutle Run (m)	815.8 ± 382.7	445.1 ± 199.3	^***^

**Notes.**

BMI body mass index

For age [(*F*(*df*:5960) = 183.9); *p* < 0.001)].

*** significant for *p* < 0.001.

In general, male subjects showed greater weight and stature and better performance in push-ups, standing long jump, and 20-m shuttle run (*p* < 0.001). On the contrary, female students demonstrated better performance in the sit-and-reach test. No significate differences were observed in the BMI (*p* = 0.3345). However, females showed a greater sum of skinfolds than males.

**Figure 3:**
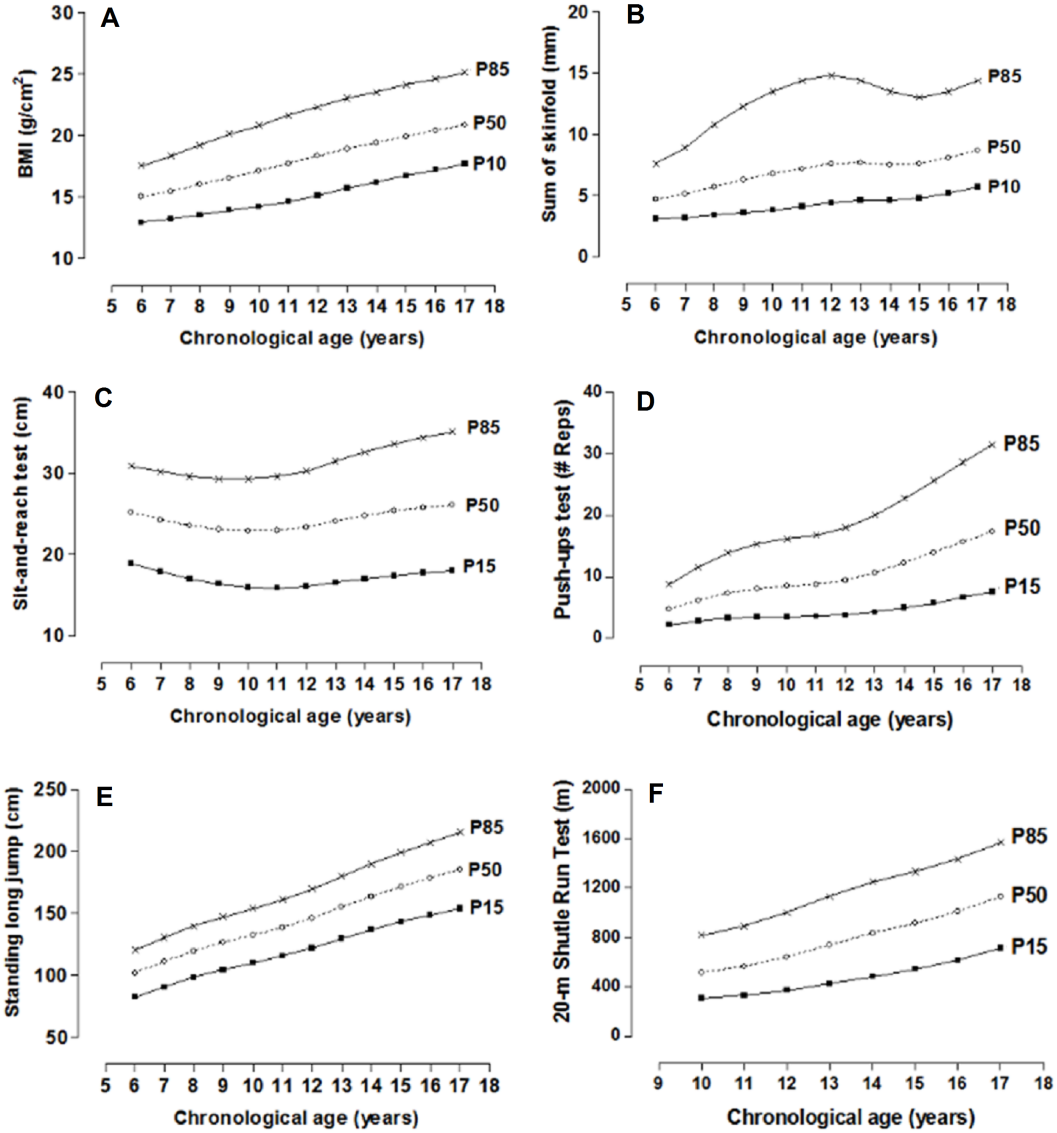
Smoothed centile curves for BMI and Skinfolds (P10, P50 and P85) and physical fitness tests (P15, P50 and P85) by chronological age in males.

**Figure 4:**
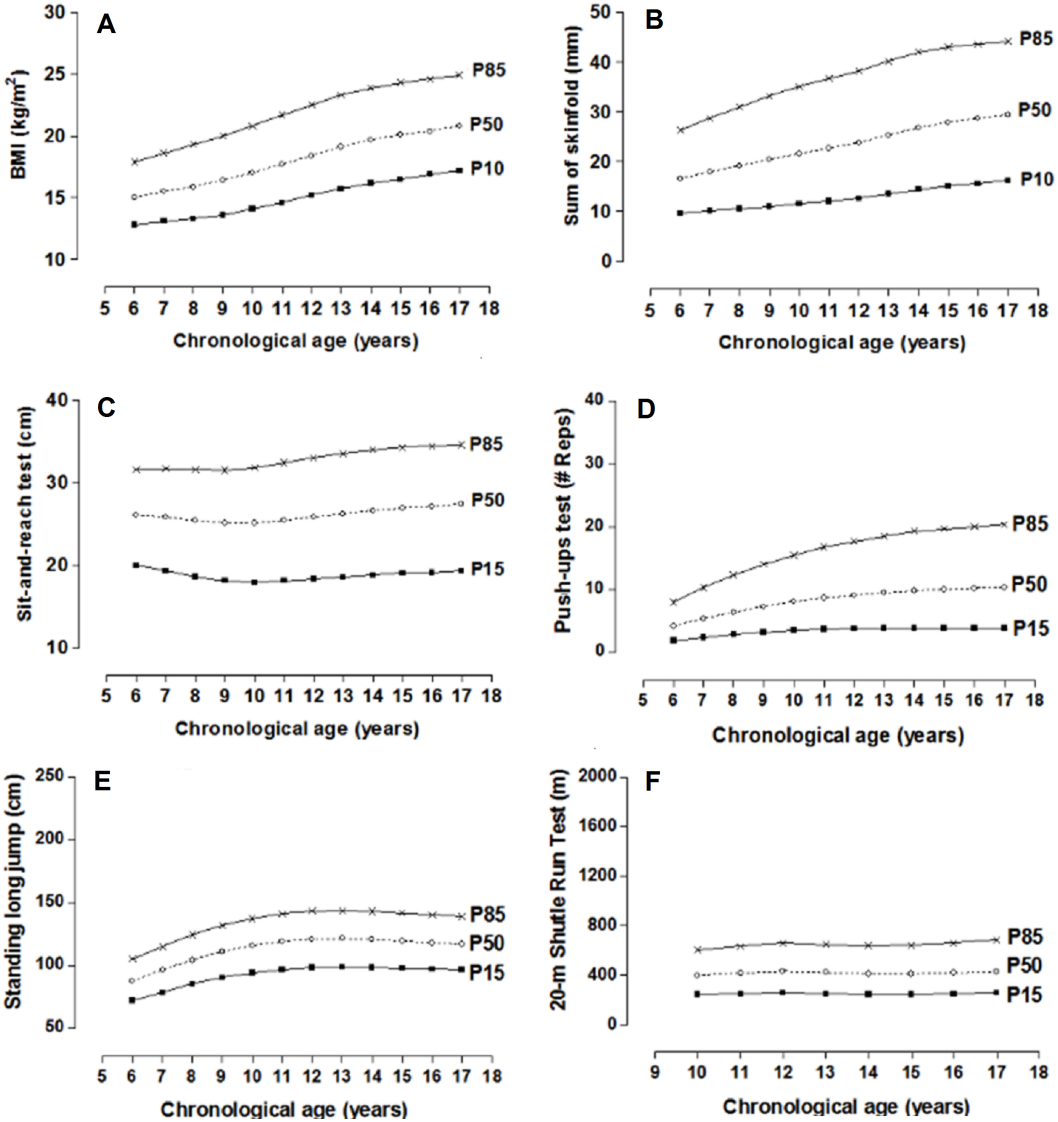
Smoothed centile curves for BMI and Skinfolds (P10, P50 and P85) and physical fitness tests (P15, P50 and P85) by chronological age in females.

The physical fitness dimension norms distributed in percentiles by age and sex are illustrated in Tables 2 and 3. For the morphological dimension (BMI and skinfolds), the P10, P50, and P85 percentiles have been used and for the other dimensions in P15, P50, and P85. Smoothed curves are also shown through the LMS method for the four dimensions of physical fitness by age and sex (Figs. 3 and 4).

**Table 2:** Smooth centile scores for body mass index (BMI), Skinfolds, sit-and-reach, push-ups test, standing long jump, and **20-m shuttle run** by chronological age in males.

Age	n	L	M	S	P10	p50	P85	L	M	S	P15	p50	P85	L	M	S	P15	p50	P85
		BMI (kg/m^2^)						Sit-and-reach test (cm)						Standing long jump (cm)					
6.0–6.9	284	−2.03	14.96	0.13	12.9	15.0	17.5	1.42	25.17	0.23	18.9	25.2	30.9	1.32	101.75	0.18	82.4	101.7	120.0
7.0–7.9	222	−1.91	15.44	0.14	13.2	15.4	18.3	1.40	24.32	0.24	17.9	24.3	30.2	1.29	110.90	0.17	90.5	110.9	130.2
8.0–8.9	210	−1.79	15.99	0.15	13.5	16.0	19.2	1.37	23.62	0.26	17	23.6	29.6	1.26	119.28	0.17	98.1	119.3	139.5
9.0–9.9	227	−1.67	16.53	0.16	13.9	16.5	20.1	1.34	23.13	0.27	16.4	23.1	29.3	1.24	126.26	0.16	104.4	126.3	147.2
10.0–10.9	236	−1.56	17.08	0.17	14.2	17.1	20.8	1.30	22.95	0.28	16	22.9	29.3	1.21	132.26	0.16	109.9	132.3	153.9
11.0–11.9	244	−1.46	17.66	0.17	14.6	17.7	21.6	1.24	23.02	0.28	15.9	23	29.6	1.17	138.55	0.16	115.5	138.5	161.0
12.0–12.9	297	−1.43	18.28	0.17	15.1	18.3	22.3	1.18	23.42	0.29	16.1	23.4	30.3	1.14	146.08	0.16	122.0	146.1	169.6
13.0–13.9	354	−1.47	18.85	0.17	15.7	18.9	23.0	1.10	24.14	0.30	16.6	24.1	31.5	1.12	154.85	0.16	129.5	154.9	179.7
14.0–14.9	326	−1.57	19.36	0.16	16.2	19.4	23.5	1.01	24.82	0.30	17	24.8	32.6	1.14	163.54	0.16	136.7	163.5	189.8
15.0–15.9	241	−1.70	19.87	0.16	16.7	19.9	24.1	0.91	25.41	0.31	17.4	25.4	33.6	1.18	171.38	0.16	143.0	171.4	199.0
16.0–16.9	167	−1.83	20.37	0.15	17.2	20.4	24.6	0.80	25.84	0.31	17.8	25.8	34.4	1.23	178.45	0.16	148.6	178.5	207.2
17.0–17.9	130	−1.96	20.85	0.15	17.7	20.8	25.1	0.69	26.14	0.32	18	26.1	35.1	1.30	185.35	0.16	153.9	185.4	215.3
		Σ skinfolds (TR+SB)						Push-ups (# Reps)						20-m Shuttle run (m)					
6.0–6.9	284	−0.78	4.69	0.39	3.1	4.7	7.6	0.33	4.80	0.65	2	5	9						
7.0–7.9	222	−0.74	5.08	0.44	3.2	5.1	8.9	0.35	6.24	0.67	3	6	12						
8.0–8.9	210	−0.73	5.72	0.49	3.4	5.7	10.8	0.36	7.38	0.68	3	7	14						
9.0–9.9	227	−0.62	6.33	0.52	3.6	6.3	12.3	0.39	8.14	0.70	4	8	15						
10.0–10.9	236	−0.60	6.81	0.54	3.8	6.8	13.5	0.41	8.53	0.71	4	9	16	0.23	515.79	0.47	308.1	515.8	817.4
11.0–11.9	244	−0.63	7.21	0.54	4.1	7.2	14.4	0.44	8.84	0.72	4	9	17	0.33	565.85	0.47	331.2	565.9	892.5
12.0–12.9	297	−0.67	7.57	0.52	4.4	7.6	14.8	0.47	9.52	0.72	4	10	18	0.43	642.75	0.47	370.8	642.8	1,003.0
13.0–13.9	354	−0.73	7.67	0.49	4.6	7.7	14.4	0.49	10.73	0.71	4	11	20	0.54	739.81	0.46	424.6	739.8	1,133.6
14.0–14.9	326	−0.73	7.54	0.46	4.6	7.5	13.5	0.50	12.27	0.70	5	12	23	0.64	835.25	0.44	484.4	835.2	1,249.0
15.0–15.9	241	−0.67	7.62	0.43	4.8	7.6	13.0	0.49	13.97	0.69	6	14	26	0.73	915.46	0.42	543.0	915.5	1,334.2
16.0–16.9	167	−0.75	8.07	0.41	5.2	8.1	13.5	0.48	15.70	0.67	7	16	29	0.81	1,010.66	0.39	617.7	1,010.7	1,436.3
17.0–17.9	130	−0.82	8.68	0.40	5.7	8.7	14.4	0.47	17.41	0.66	8	17	32	0.87	1,131.05	0.36	715.4	1,131.0	1,567.9

**Notes.**

BMI body mass index TR+SB triceps + subscapular P percentile L skew M median S coefficient of variation

**Table 3:** Smooth centile scores for body mass index (BMI), Skinfolds, sit-and-reach, push-ups test, standing long jump, and 20-m shuttle run by chronological age in females.

Age	n	L	M	S	P10	p50	P85	L	M	S	P15	p50	P85	L	M	S	P15	p50	P85
		BMI (kg/m^2^)						Sit-and-reach test (cm)						Standing long jump (cm)					
6.0–6.9	241	−1.7	15.05	0.146	12.8	15.0	17.9	1.53	26.12	0.21	19.9	26.1	31.6	0.43	87.52	0.18	71.8	87.5	105
7.0–7.9	203	−1.58	15.49	0.154	13.1	15.5	18.6	1.45	25.83	0.23	19.3	25.8	31.7	0.64	96.09	0.18	78.5	96.1	114.9
8.0–8.9	170	−1.47	15.92	0.161	13.3	15.9	19.3	1.37	25.41	0.24	18.6	25.4	31.6	0.84	104.36	0.18	85	104.4	124.3
9.0–9.9	218	−1.38	16.41	0.166	13.6	16.4	20.0	1.29	25.08	0.26	18.1	25.1	31.5	1.01	111.13	0.18	90.3	111.1	131.9
10.0–10.9	276	−1.31	17.04	0.17	14.1	17.0	20.8	1.22	25.07	0.27	17.9	25.1	31.8	1.13	115.88	0.18	94	115.9	137.3
11.0–11.9	323	−1.24	17.75	0.172	14.6	17.7	21.7	1.18	25.4	0.27	18.1	25.4	32.4	1.19	119.20	0.18	96.6	119.2	141.1
12.0–12.9	311	−1.17	18.45	0.172	15.2	18.4	22.5	1.16	25.81	0.27	18.3	25.8	33	1.18	121.03	0.18	98.1	121	143.2
13.0–13.9	337	−1.12	19.12	0.171	15.7	19.1	23.3	1.18	26.22	0.27	18.5	26.2	33.5	1.11	121.40	0.18	98.6	121.4	143.8
14.0–14.9	332	−1.08	19.67	0.169	16.2	19.7	23.9	1.22	26.64	0.27	18.8	26.6	34	1.01	120.67	0.18	98.3	120.7	143
15.0–15.9	276	−1.04	20.06	0.167	16.5	20.1	24.3	1.27	26.93	0.27	19	26.9	34.3	0.86	119.29	0.18	97.6	119.3	141.6
16.0–16.9	198	−1.00	20.40	0.164	16.9	20.4	24.6	1.33	27.12	0.27	19.1	27.1	34.4	0.68	117.93	0.18	97	117.9	140.2
17.0–17.9	139	−0.96	20.77	0.162	17.2	20.8	24.9	1.39	27.38	0.27	19.3	27.4	34.6	0.48	116.72	0.18	96.5	116.7	139
		Σ skinfolds (TR+SB)						Push-ups (# Reps)						20-m Shuttle run (m)					
6.0–6.9	241	−0.59	16.60	0.39	9.6	16.6	26.3	0.27	4.16	0.69	2	4	8						
7.0–7.9	203	−0.52	17.92	0.40	10.1	17.9	28.7	0.29	5.38	0.69	2	5	10						
8.0–8.9	170	−0.46	19.17	0.42	10.6	19.2	31.0	0.30	6.45	0.69	3	6	12						
9.0–9.9	218	−0.41	20.41	0.42	11.0	20.4	33.2	0.32	7.33	0.7	3	7	14						
10.0–10.9	276	−0.37	21.58	0.43	11.5	21.6	35.1	0.34	8.06	0.71	4	8	16	0.36	398.11	0.43	245	398.1	602.1
11.0–11.9	323	−0.33	22.63	0.43	12.0	22.6	36.7	0.35	8.67	0.72	4	9	17	0.37	414.97	0.44	251.2	415	633.2
12.0–12.9	311	−0.30	23.77	0.43	12.6	23.8	38.2	0.36	9.09	0.73	4	9	18	0.39	429.7	0.45	256.6	429.7	660.6
13.0–13.9	337	−0.27	25.32	0.42	13.5	25.3	40.2	0.38	9.48	0.74	4	10	19	0.39	419.45	0.46	248.8	419.4	647.8
14.0–14.9	332	−0.24	26.84	0.41	14.4	26.8	42.0	0.39	9.83	0.74	4	10	19	0.37	410.71	0.46	242.9	410.7	636.9
15.0–15.9	276	−0.20	27.93	0.40	15.1	27.9	43.0	0.41	10.02	0.75	4	10	20	0.34	410.87	0.46	242.8	410.9	641.8
16.0–16.9	198	−0.17	28.71	0.39	15.6	28.7	43.6	0.43	10.18	0.75	4	10	20	0.28	417.53	0.47	247.6	417.5	658.8
17.0–17.9	139	−0.13	29.44	0.38	16.2	29.4	44.1	0.44	10.37	0.76	4	10	20	0.18	428.36	0.47	256.5	428.4	684.3

**Notes.**

BMI body mass index TR+SB triceps + subscapular P percentile L skew M median S coefficient of variation

## Discussion and Conclusions

This study developed percentiles based on the LMS method to evaluate the physical fitness of children and adolescents of both sexes and ages 6.0 to 17.9. The battery of the tests given in the study evaluated four dimensions: For example, the morphological dimensions were determined by BMI and the sum of two skinfolds (tricipital + subescapular), the muscular dimension by the Push-up Test and standing long jump, and flexibility the Sit-and-reach Test, and the cardio-respiratory dimension by the Shuttle Run Test (20 m).

Therefore, to date, no general consensus exists about the definition of the key components and/or dimensions of physical fitness. However, the majority of the studies are in agreement about two defined objectives: health and sports achievements (Cvejić, Pejović & Ostojić, 2013).

In general, independent of the objectives, both depend on the level of physical fitness performed inside and outside of the school system. The percentile values proposed in this study may be useful for identifying children and adolescents with high health risks as well as identifying individuals with moderate and elevated levels of physical fitness.

Various studies have proven that a low level of physical fitness during childhood and adolescence is associated with overweight and obesity as well as cardiovascular diseases, deterioration of bone health, and reduced quality of life (Vicente-Rodriguez et al., 2008; Moliner-Urdiales et al., 2010; Morales et al., 2013), among other ailments.

For example, increase in muscular strength from childhood to adolescences related inversely to changes in body fat. (Ruiz et al., 2011). Moreover, it has been demonstrated that strength is positively associated with a better quality of bone health (Pitukcheewanont, Punyasavatsut & Feuille, 2010). In addition, an adequate level of aerobic conditioning increases the capacity to work efficiently and allow participation and enjoyment in physical activities such as sports, recreation, and leisure (Carnethon, Gulati & Greenland, 2005). Therefore, these benefits are associated with adequate levels of flexibility, reduced risks of lesions, prevent and reduce pain, and improve motor coordination (Castro-Piñero et al., 2010).

As a result, the importance of evaluating body fat variables and physical fitness tests plays an important role in monitoring the level s of physical activity and physical fitness of populations in general. According to the World Health Organization (2010), physical fitness should be considered a priority for public health. Therefore, schools should play a central role in the provision and promotion of physical activity and physical fitness of the young along with other healthy behaviors since children and adolescents spend a majority of their time in the school setting (Pate et al., 2006).This is an important opportunity to introduce physical activity programs for specific work groups, not only in schools but also in sports clubs and/or physical sports rehabilitation centers, respectively.

The accurate interpretation of physical fitness values requires the comparison of the scores obtained from one person in particular with the normative valuies for the general population of the same sex and age (Ortega et al., 2011). In this context, the normative values proposed here may be used for different purposes, for example morphological dimensions (BMI and skinfolds). The interpretation of >p85 may be considered excessive fat, between 10 to p85 as normal, and <p10 as low levels of fat equal to the CDC-2000 cut-off scores (Kuczmarski et al., 2000). On the other hand, for the physical fitness tests, the percentiles less than <p15 may be interpreted as a low level or a warning sign, between p15 to p85 as adequate, and >p85 as an elevated level of fitness.

In essence, to date, no defined consensus exists about the cut-off points for dimensions used in the tests for determining physical fitness in pediatric populations. In spite of this, some population studies use scales consisting of two to five categories (Meredith, Welk & the Cooper Institute, 2007; Ortega et al., 2011; Catley & Tomkinson, 2013; De Miguel-Etayo et al., 2014).

This current study opts for three categories. To do this, we took into account the reality of the educational systems of the schools since the schools, just importantly, identified students with low levels of physical fitness as well as identifying those student athletes (Golle et al., 2015).

Moreover, the schools maintained and promoted adequate levels of physical activity (related to health) for the students throughout the school years. Furthermore, this is an objective that should be maintained in the short, medium, and long term. Thus, these objectives should not be careless and of a permanent nature. They should be developed within the curriculum programs in all of the educational systems.

In summary, taking into account the probabilistic sample selection, the use of the LMS method to generate the percentiles and the proposed battery of tests with their four dimensions, this study has a number of advantages. Based on its applicability to the Lake Itaipú region of Brazil and the selected sample size, the results from this research may be generalized to other geographical regions of Brazil, particularly those with similar demographic characteristics.

This study has a few limitations. For example, the (cross-sectional) design of the study does not allow for changes to be described in physical fitness during growth and development. Therefore, future studies should be designed longitudinally since physical fitness levels change over time. Longitudinal research is better suited to track changes over time than are cross-sectional studies (Golle et al., 2015). Furthermore, biological maturation could not be measured in this study. Controlling for this variable would have diminished the range of variability between individuals of the same chronological age during adolescence.

In conclusion, the results of this study show regional reference values for evaluating the physical fitness of children and adolescents by chronological age and sex. The findings from this research can be used for the detection and monitoring of the four dimensions of physical fitness (morphological, flexibility, muscle strength, and cardiorespiratory) as a specific tool for health in educational contexts. Nevertheless, to confirm these results, it is necessary to develop longitudinal studies.

##  Supplemental Information

10.7717/peerj.4032/supp-1 Data S1 Raw data

## Additional Information and Declarations

### Data Availability

The following information was supplied regarding data availability: Figshare DOI: 10.6084/m9.figshare.5388298.
