# Supplementary material for: Reference standards to assess physical fitness of children and adolescents of Brazil: an approach to the students of the Lake Itaipú region—Brazil
Source: PeerJ. 2017 Nov 30;5:e4032. doi: 10.7717/peerj.4032 (PMC5712463; doi:10.7717/peerj.4032)
Supplement: Data S1 [file peerj-05-4032-s001.pdf]

Age	sexo	Weight	Height	Standing long	Sit-and-reach	Push-ups
6	1	19,3	115,6	60	26	3
6	1	20,9	119,5	108	25	5
6	1	21,2	115,1	74	28	1
6	1	27,8	134,3	119	30	6
6	1	22,2	117,8	96	35	11
6	1	19,7	113,8	100	26	5
6	1	20,6	117,1	120	28	17
6	1	25	120	128	40	6
6	1	27,8	131	110	33	7
6	1	19	115,2	107	25	6
6	1	29,7	126,2	90	24	2
6	1	19,4	114,9	90	28	6
6	1	22,5	119,2	75	24	2
6	1	21,9	118,5	110	25	10
6	1	23,6	120,8	62	26	3
6	1	23,6	117,1	123	21	4
6	1	25,7	123,2	102	29	5
6	1	25	123,8	91	21	2
6	1	22,3	123,6	108	20	7
6	1	20,5	126	94	24	3
6	1	20,7	118,2	102	28	4
6	1	23,6	120	89	17	5
6	1	22,3	118,9	50	24	2
6	1	21,7	118,7	122	29	15
6	1	21,1	119	110	12	12
6	1	19,2	116,5	130	10	6
6	1	18,2	115,4	106	11	4
6	1	26,6	124,9	104	27	6
6	1	20,2	120	130	23	9
6	1	23	123,4	105	23	6
6	1	20,5	123,5	72	29	1
6	1	23,2	128,3	105	26	8
6	1	23,8	124	117	19	7
6	1	19,3	117,5	85	23	7
6	1	23	117	110	12	6
6	1	21,1	121	141	19	8
6	1	30,2	123	64	25	3
6	1	29,6	121,5	101	21	4
6	1	28,3	121	80	10	4
6	1	25	117,5	139	26	2
6	1	26,6	122,4	64	20	4
6	1	34,3	134	73	22	4
6	1	32,9	129,8	70	32	5
6	1	26	120,5	100	22	1
6	1	18,8	122,3	114	30	5
6	1	24,2	118	135	28	10
6	1	20,6	119,9	110	28	4
6	1	25,4	132,1	94	21	3
6	1	24,5	125,5	58	34	1

6	1	18,7	118	71	20	1
6	1	21	121,5	68	25	3
6	1	25,2	123,4	123	25	7
6	1	19,3	114,1	70	32	2
6	1	24,7	123,4	98	23	5
6	1	20,9	119,8	83	25	5
6	1	23,2	118,9	112	24	2
6	1	20,5	114,1	80	30	3
6	1	20,3	108,4	87	21	6
6	1	19,6	118,2	82	31	8
6	1	22,5	124,7	95	24	4
6	1	24,3	119,5	60	19	2
6	1	20,1	117,9	91	27	7
6	1	20	121,4	120	19	10
6	1	22,4	119,6	102	26	5
6	1	17,3	115,7	88	31	1
6	1	24,7	124,3	68	20	4
6	1	18,4	116,8	106	26	4
6	1	21,6	121	90	30	5
6	1	27	129,9	129	32	3
6	1	23,2	122,6	119	36	12
6	1	23,8	119,2	105	25	8
6	1	21	119,4	96	32	5
6	1	21,7	124,2	70	23	2
6	1	26,1	127,5	72	10	5
6	1	17,4	114,9	78	23	4
6	1	26,4	125,1	116	28	12
6	1	21,7	118,2	95	31	5
6	1	20,9	120,5	112	21	5
6	1	23,7	117,1	100	24	7
6	1	22,8	122,1	80	26	2
6	1	16,6	113,8	90	27	10
6	1	18	112,5	94	22	12
6	1	20,8	125	75	20	6
6	1	25,2	124,3	100	18	4
6	1	23,4	121,3	121	31	10
6	1	23,1	121,5	95	28	11
6	1	27,4	131,4	95	21	4
6	1	20,9	114,6	109	20	10
6	1	27,4	126,5	135	31	5
6	1	21,1	123	120	31	10
6	1	20,4	117,5	78	31	10
6	1	20,4	122,9	80	12	2
6	1	21,3	115,6	100	24	9
6	1	27,2	129,5	106	21	2
6	1	21,6	122,2	100	27	4
6	1	22,6	120,2	100	28	7
6	1	26,4	130,5	117	20	1
6	1	26,5	128,6	110	26	8
6	1	20,9	120,5	145	27	4

6	1	24,6	128,5	127	27	11
6	1	22,4	122,1	118	29	5
6	1	24	122,1	114	21	8
6	1	25,5	126,4	85	26	2
6	1	22,4	116	89	27	2
6	1	20,7	115,5	121	33	9
6	1	24,7	122,8	98	23	3
6	1	19,7	119,5	112	20	2
6	1	21,5	128,2	100	24	8
6	1	26,5	122,5	130	26	6
6	1	23,2	121,1	120	22	5
6	1	27,5	129	115	31	10
6	1	20,2	119,4	75	21	2
6	1	27,8	129,7	105	36	5
6	1	21,4	119,1	122	21	9
6	1	30,6	135	100	27	5
6	1	25,9	126,2	119	38	4
6	1	24,5	127	124	27	6
6	1	17,1	112,1	100	24	3
6	1	19,6	114,8	103	25	8
6	1	20,9	119,1	95	22	3
6	1	19,2	115,3	70	23	5
6	1	24,4	118,6	93	27	1
6	1	25,2	125,3	64	15	5
6	1	26,3	124	112	29	9
6	1	20,2	119,6	90	21	3
6	1	23,4	123,8	105	25	5
6	1	23,3	123,5	112	26	5
6	1	29,1	128,2	128	25	9
6	1	27,3	129,2	95	18	3
6	1	42,9	127	73	30	2
6	1	36,5	127,5	69	22	7
6	1	42,7	131	70	17	1
6	1	37,5	121,9	74	27	3
6	1	26,3	122	89	23	4
6	1	29,3	126,1	90	32	4
6	1	25,6	119,2	88	31	3
6	1	28,7	120,9	125	32	5
6	1	27,1	121,2	125	16	3
6	1	30,3	126,1	106	22	8
6	1	33,4	133,5	104	26	4
6	1	32,3	133,7	129	26	3
6	1	29,4	123,8	115	22	5
6	1	29,7	124,9	85	22	2
6	1	28,1	125,5	103	25	5
6	1	20,7	115,1	110	33	8
6	1	17,3	108,9	97	27	1
6	1	17,4	111	103	31	6
6	1	25,4	122	88	27	2
6	1	19,1	118,9	133	26	3

6	1	21	120	76	20	1
6	1	17	116,2	78	24	1
6	1	16,8	109,9	83	23	5
6	1	22	120	90	25	3
6	1	23,9	121,8	134	31	6
6	1	19,8	124,6	83	22	2
6	1	20	114	103	33	8
6	1	18,3	116,5	96	30	4
6	1	20,5	118	92	22	2
6	1	20,2	112,6	130	29	12
6	1	21	114,5	90	26	5
6	1	15,7	112,1	65	29	4
6	1	18,5	117,1	106	26	2
6	1	18,1	111,2	108	22	5
6	1	26,4	125,2	108	21	4
6	1	20,3	115,5	100	33	3
6	1	20,1	118,2	60	28	4
6	1	23	123	105	24	1
6	1	18,7	118,3	95	24	4
6	1	19,6	115,2	115	28	8
6	1	28,4	129,5	98	24	6
6	1	15,1	111,8	57	21	1
6	1	17	112,4	50	23	1
6	1	20,8	120	82	30	5
6	1	20,7	119,2	90	25	6
6	1	20,4	119,1	70	23	6
6	1	21,7	116,6	123	26	2
6	1	20,2	117,2	104	27	7
6	1	23,2	119,4	89	25	4
6	1	19,9	125,3	95	25	3
6	1	21	114,5	105	35	9
6	1	21,8	116,9	133	28	9
6	1	22,4	124	105	26	5
6	1	28,6	130,5	110	18	10
6	1	24	123,2	112	30	3
6	1	20,3	116,1	80	25	6
6	1	21,2	122,6	89	29	1
6	1	17,4	114,5	100	21	1
6	1	24,1	123,8	93	21	1
6	1	24,4	121	109	29	2
6	1	19,2	111,5	71	26	5
6	1	29,6	138,5	110	31	8
6	1	28,1	140	127	35	7
6	1	23,7	127	110	24	4
6	1	19,1	117	76	24	3
6	1	23,2	126,3	98	15	2
6	1	21	117	95	23	4
6	1	22,6	122,2	115	32	3
6	1	27,2	124,1	112	26	12
6	1	21,2	116,5	128	32	7

6	1	25,9	120,9	102	28	10
6	1	24,5	125	102	18	1
6	1	19,6	124,8	130	25	5
6	1	28,1	125,3	81	29	4
6	1	22	121,1	98	22	5
6	1	27,8	131,2	90	30	5
6	1	17,7	118,9	131	23	7
6	1	23,5	121	80	18	4
6	1	20,8	116	106	25	16
6	1	25,2	131,2	132	12	6
6	1	23,2	126,6	75	23	2
6	1	25,3	127,1	109	30	11
6	1	17,7	116,6	98	21	2
6	1	20,9	124,1	103	34	5
6	1	24,2	123,1	109	26	5
6	1	21,6	121	95	23	10
6	1	22,5	121,2	108	21	4
6	1	28,5	126,2	120	31	10
6	1	22	121,8	105	24	9
6	1	17,6	118	114	34	8
6	1	25,9	122,5	77	22	5
6	1	22,1	119,5	100	18	6
6	1	21,2	120,6	107	38	7
6	1	20,1	118,5	115	33	12
6	1	21,2	123,1	102	18	7
6	1	24,2	119	143	6	7
6	1	21,1	121	149	30	6
6	1	22,6	115	140	39	14
6	1	19,6	121,9	100	31	6
6	1	23,2	125,6	100	27	4
6	1	25,8	125,1	95	26	5
6	1	16,3	111,8	94	17	3
6	1	24,3	124	114	36	3
6	1	19,9	121	97	34	10
6	1	30,8	120	67	31	2
6	1	49,2	134,5	60	12	1
6	1	36,4	128,5	95	26	6
6	1	28,2	123,5	92	33	5
6	1	26,8	121,8	95	30	12
6	1	31,5	127	83	20	4
6	1	30,9	127,2	116	22	8
6	1	30,7	127	100	17	1
6	1	27,5	121,4	63	25	9
6	1	33,1	128,3	94	19	10
6	1	32,3	133,2	129	26	1
6	1	30,7	128,8	100	27	2
6	1	22	119,9	106	31	10
6	1	17	108,2	112	34	7
6	1	23,1	119,9	100	32	2
6	1	22,5	119,4	69	25	1

6	1	19,5	119,6	100	23	8
6	1	21,2	121,9	94	31	1
6	1	21,1	120	72	22	10
6	1	21,4	127,1	104	23	5
6	1	24,8	121,2	98	15	5
6	1	25,4	129,8	100	21	3
6	1	21,8	122,8	104	26	2
6	1	21,6	121,6	110	30	5
6	1	19,6	114,5	51	20	2
6	1	25,8	126	115	20	9
6	1	20,3	117,9	84	29	4
6	1	22,1	132,1	104	23	3
6	1	22,1	120,9	135	29	5
6	1	25,3	126,9	98	21	3
6	1	28,2	129,8	100	25	3
6	1	25,2	127,4	90	22	10
6	1	27,8	130,2	121	18	4
6	1	25,9	127,5	118	22	6
6	1	21,7	124,9	134	15	7
6	1	22,4	126,2	130	29	11
6	1	21,6	120,1	128	29	11
6	1	25,2	125,6	103	24	3
6	1	48,7	138,5	100	29	3
6	1	35,6	128	90	28	13
6	1	28,9	127	90	21	3
6	1	31	129,7	107	35	2
6	1	34,6	134,2	100	25	8
6	1	36,1	134,5	90	24	8
6	1	29	123	116	16	8
6	1	24,8	121,3	66	23	1
6	1	19,8	117,5	103	29	5
6	1	19,8	115,3	119	25	9
6	1	19,1	117,1	115	33	5
6	1	23,9	120,5	125	23	9
6	1	27,3	137,8	118	23	12
6	2	18,9	113,3	110	33	3
6	2	22,7	119	110	21	6
6	2	23	123	100	26	3
6	2	23,2	124,5	97	29	4
6	2	23,3	122,8	70	28	6
6	2	31,8	127,6	98	19	3
6	2	26,1	121	87	24	2
6	2	21,2	111,9	101	25	4
6	2	19,3	120,1	66	27	4
6	2	19,6	114	105	35	7
6	2	20	119,2	61	22	3
6	2	20,6	118,2	95	28	5
6	2	25,5	122,8	93	22	4
6	2	17,6	114,2	68	25	4
6	2	19,7	120,3	85	22	4

6	2	19,3	116,5	110	31	1
6	2	17,4	115	82	30	2
6	2	21,9	120,4	100	32	4
6	2	21	119	84	27	7
6	2	22,4	125	127	21	15
6	2	20,6	120,9	138	25	4
6	2	20	121,5	60	22	3
6	2	28,6	132,9	110	31	3
6	2	22,6	128,8	95	31	4
6	2	23,9	122,4	98	28	1
6	2	20,5	127,2	88	30	8
6	2	23,8	123,8	78	20	1
6	2	22,2	122,5	72	21	8
6	2	24	120,5	67	15	1
6	2	20	122	87	26	1
6	2	22,3	122,5	114	29	6
6	2	24,6	118	80	23	3
6	2	22,7	113	74	26	5
6	2	28,2	125	100	31	9
6	2	24,7	118,1	65	24	1
6	2	28,2	122	92	30	2
6	2	28,5	121,5	91	16	12
6	2	20,9	120,3	90	30	2
6	2	19,2	117,1	92	24	3
6	2	21,2	119,1	103	27	6
6	2	24,7	121,5	131	37	5
6	2	20,7	120,9	88	29	10
6	2	17,9	111,3	70	20	1
6	2	16,6	109	70	30	8
6	2	25,2	125,6	85	20	4
6	2	17,3	111,2	73	16	2
6	2	19,5	119,2	89	27	6
6	2	22,4	119,4	86	27	2
6	2	23,6	118,2	60	32	10
6	2	22,3	120,7	85	29	1
6	2	18,8	112,7	86	30	5
6	2	17,6	115,2	98	25	13
6	2	24	123	65	25	3
6	2	21,2	125,6	90	24	3
6	2	25	127,1	102	33	3
6	2	20,6	118,1	96	29	4
6	2	17,5	118,1	83	26	3
6	2	20,7	121,2	92	20	2
6	2	26,4	133,1	92	24	4
6	2	19,1	117,6	72	28	2
6	2	19,6	117,5	87	27	4
6	2	24,7	121,5	90	29	1
6	2	18,3	118,9	80	30	4
6	2	18,1	117,9	105	24	2
6	2	26,2	123	74	25	9

6	2	19,9	115	55	24	10
6	2	18	112	115	35	5
6	2	17,3	117,2	85	29	1
6	2	25,7	125,2	104	21	8
6	2	24,4	124,8	90	27	5
6	2	18,5	115,2	72	24	4
6	2	23	119,6	68	35	10
6	2	21,3	122,5	80	16	5
6	2	25,4	123,8	62	29	3
6	2	21,1	122,8	92	32	5
6	2	22,8	121	100	30	4
6	2	20,3	117,8	88	33	5
6	2	25,1	120,8	104	27	5
6	2	20,9	111,1	60	22	4
6	2	22,5	122,9	70	21	9
6	2	30,9	132	93	29	3
6	2	23,1	116,1	92	26	5
6	2	22,2	122	110	23	13
6	2	23,1	129	98	25	7
6	2	21,3	120,2	95	24	2
6	2	28,9	133,5	80	28	6
6	2	19,8	118,5	133	21	8
6	2	20,7	115,5	90	26	5
6	2	24,9	120,1	103	29	6
6	2	23,6	126	124	26	10
6	2	20,4	119,8	116	32	8
6	2	22	119,5	70	28	8
6	2	23,9	123	105	34	2
6	2	25,4	129,6	90	31	7
6	2	18,1	119,5	100	30	3
6	2	23,6	129,3	85	28	10
6	2	20,4	117,1	101	28	6
6	2	20,2	120	83	20	9
6	2	26	125,1	101	35	4
6	2	21,3	124,9	80	25	12
6	2	25,5	132,1	125	26	4
6	2	16,1	113,3	78	28	1
6	2	22,4	121,5	72	10	1
6	2	21,9	118,2	104	30	6
6	2	23,4	120,5	117	32	1
6	2	23	120,9	119	24	5
6	2	21,9	122,7	120	27	4
6	2	22,5	126,8	92	19	8
6	2	22,7	120	78	27	6
6	2	44,5	135,2	60	16	1
6	2	33,7	127,2	60	10	1
6	2	38,7	129,7	72	11	1
6	2	33,7	125,2	100	20	2
6	2	36,6	122,3	78	25	1
6	2	37,8	125,2	81	35	5

6	2	31,7	121,1	83	32	4
6	2	33,7	123,8	84	28	1
6	2	31,4	133,2	82	22	7
6	2	23,9	114,1	104	25	8
6	2	25,6	118,6	88	27	4
6	2	25,2	116,2	70	28	3
6	2	31,8	128,1	77	20	2
6	2	29,1	126,5	66	28	2
6	2	35	130,8	77	16	7
6	2	29,7	121	90	31	8
6	2	31,8	127	81	24	3
6	2	31,2	124,6	82	28	3
6	2	31,1	127,2	111	29	1
6	2	34,9	131,8	104	18	2
6	2	17,5	108,6	71	26	2
6	2	18,6	120,3	69	30	1
6	2	17,3	111,6	70	25	4
6	2	20,2	112	81	27	1
6	2	24,2	125,1	103	19	1
6	2	23,3	118,1	70	20	1
6	2	16	106,1	73	25	1
6	2	23,5	121,6	82	26	4
6	2	23,2	118,1	84	24	2
6	2	19,8	119,2	84	3	8
6	2	21	120,3	79	26	3
6	2	23,5	123	80	26	5
6	2	20,8	110,2	57	35	1
6	2	21,3	124,1	94	23	3
6	2	18,1	110	50	26	4
6	2	21,6	121,2	90	10	7
6	2	24,4	125,9	97	29	8
6	2	24,2	118,4	85	26	2
6	2	20,3	119,4	89	18	2
6	2	24,3	119,2	68	27	4
6	2	18,2	117,6	73	25	4
6	2	25,1	123,2	80	21	1
6	2	25,6	126,5	88	31	3
6	2	19,1	117	71	25	3
6	2	24,3	121,5	93	22	4
6	2	24,7	122,1	82	23	4
6	2	19,2	114	90	32	6
6	2	24,1	119,8	86	22	6
6	2	15,7	117	84	23	3
6	2	19,5	114,5	105	28	2
6	2	18,9	115	132	30	7
6	2	16,2	107,6	62	27	5
6	2	18,7	119,3	78	15	2
6	2	26,1	124	80	25	2
6	2	21,5	116,3	104	26	6
6	2	22	119	94	27	7

6	2	22,3	119,1	107	32	10
6	2	18,3	116,3	65	25	1
6	2	21,2	118,3	121	27	4
6	2	16,1	109,5	83	23	3
6	2	23,9	124,1	87	20	2
6	2	23,4	124,1	90	22	7
6	2	25,1	123,8	80	21	6
6	2	20,1	117	75	23	3
6	2	21,6	124	80	25	5
6	2	26,1	128,2	73	20	1
6	2	21,7	121,4	117	27	2
6	2	16,3	112	82	26	8
6	2	22,3	129	80	18	5
6	2	24	129,5	85	22	10
6	2	24,6	121,7	78	19	2
6	2	18,2	114,2	90	34	9
6	2	17,2	113	77	15	3
6	2	23,5	125	94	18	3
6	2	29,1	129,6	104	29	4
6	2	25,6	128,9	106	29	20
6	2	23,9	125,4	90	23	10
6	2	18,8	113,5	98	26	4
6	2	17,8	114,6	85	23	4
6	2	19,5	112,9	87	41	9
6	2	21,9	121,5	63	31	5
6	2	22,9	123,1	87	22	8
6	2	24,2	126,2	90	29	10
6	2	24,5	121	90	24	4
6	2	33,8	127,5	88	33	3
6	2	28,6	120,5	82	25	11
6	2	33,1	124,1	78	22	1
6	2	39	125,6	75	20	1
6	2	23,5	116	100	32	11
6	2	24,8	117,1	76	29	2
6	2	27	120	78	23	2
6	2	25,2	119,6	89	13	8
6	2	25,8	121,5	94	35	5
6	2	24,9	113	100	36	6
6	2	30,5	128,2	106	34	6
6	2	28,5	125,3	102	23	8
6	2	23,8	124,2	102	25	4
6	2	16,6	114,7	95	30	5
6	2	19,4	114,2	77	31	2
6	2	22,8	122,5	110	28	5
6	2	15,8	105,2	88	22	2
6	2	22,3	124,3	80	29	1
6	2	19,3	113,2	65	28	3
6	2	21,1	121	89	33	11
6	2	22,7	122,4	81	20	5
6	2	25	124,2	93	35	10

6	2	25,2	126,2	92	27	4
6	2	25,8	127,8	95	26	13
6	2	20	121,4	80	25	8
6	2	21,4	121	99	25	1
6	2	24,2	127	120	30	2
6	2	20,6	121,8	95	29	5
6	2	19,7	118,3	65	27	1
6	2	24,3	120,7	92	29	3
6	2	21,6	119,4	81	23	8
6	2	19,4	122,1	67	18	2
6	2	24,4	127	120	33	5
6	2	17,9	117,9	85	22	3
6	2	22,4	125,2	58	28	4
6	2	40,7	133,9	97	30	5
6	2	22,9	112,1	81	29	2
6	2	37,1	134,6	105	23	2
6	2	23,7	115,5	85	28	15
6	2	25,8	119,2	89	22	3
6	2	30	127,1	80	31	4
6	2	20,7	122,1	88	22	5
6	2	24,5	120,5	78	36	6
6	2	17,7	111,8	89	33	5
6	2	23,8	120,2	85	26	3
6	2	21,5	120,2	80	37	9
7	1	31,1	134,2	117	32	8
7	1	24	123,5	103	28	4
7	1	23,2	120,1	100	24	3
7	1	17,9	121,5	95	20	2
7	1	39,1	141,8	83	20	5
7	1	22,5	127,4	114	18	8
7	1	25	125,6	140	20	7
7	1	27,5	129,5	80	13	5
7	1	27,3	128,7	97	18	1
7	1	21,6	118	126	25	10
7	1	25,1	128,6	130	25	4
7	1	27,2	131	107	18	3
7	1	21,6	120,3	132	15	8
7	1	29,4	127,6	103	30	4
7	1	32,2	135,2	120	18	11
7	1	26,2	132,1	130	29	8
7	1	19,1	118,1	119	26	3
7	1	25,2	122,5	112	29	11
7	1	29	128	125	25	5
7	1	29,7	129,2	90	12	6
7	1	26,4	134,2	141	30	7
7	1	29	136,8	83	18	5
7	1	22,6	130,4	139	21	7
7	1	23,2	127	96	21	7
7	1	26,6	133,2	130	16	9
7	1	24,2	130,7	121	30	6

7	1	25,8	125,9	140	19	10
7	1	25,4	130,5	115	28	11
7	1	26,5	125,1	105	18	5
7	1	28,4	135,5	125	29	6
7	1	23,8	115,5	93	37	12
7	1	38,4	128,5	133	36	7
7	1	45,2	141,6	99	21	7
7	1	42,1	137,2	80	14	2
7	1	35,4	135,5	92	26	3
7	1	29,7	127,5	140	25	5
7	1	34,5	134,8	99	27	1
7	1	29,7	124,6	91	12	2
7	1	36,8	135,5	111	27	5
7	1	28	114	101	21	5
7	1	25,4	131,8	110	23	3
7	1	26,8	136,9	100	26	3
7	1	28,6	134,2	95	24	8
7	1	23,6	130,5	113	16	1
7	1	29,5	129	84	32	10
7	1	19,9	118,6	126	15	4
7	1	33	135	80	5	5
7	1	24,3	122,1	99	26	12
7	1	26,1	130	116	18	9
7	1	22,4	118,9	104	29	9
7	1	22,8	120,8	144	26	10
7	1	21,4	121,8	120	27	8
7	1	24,3	127,2	117	21	5
7	1	21,8	124	93	18	7
7	1	23,6	124,5	110	22	10
7	1	23,9	125,4	110	22	20
7	1	23,7	126,5	90	24	8
7	1	24,1	120	106	28	6
7	1	19,7	118,2	105	24	7
7	1	26,4	129,3	130	28	8
7	1	18,7	121,2	115	15	6
7	1	27,3	127,3	141	6	6
7	1	21,7	121	92	10	1
7	1	24,9	128,9	138	38	3
7	1	26,2	132,4	128	23	4
7	1	22,6	128,5	106	28	9
7	1	23,9	126,2	100	24	4
7	1	28,3	134,1	119	25	10
7	1	29,7	129,3	110	25	6
7	1	23,3	121,5	119	33	18
7	1	19,4	123,1	113	21	16
7	1	19,7	124	107	33	2
7	1	20,8	122,1	104	31	9
7	1	20,7	120	91	21	3
7	1	21,2	123,5	130	35	3
7	1	22,1	123,1	110	28	6

7	1	26	127,2	145	18	8
7	1	27,8	130,6	112	23	12
7	1	23,2	118	143	30	10
7	1	30,4	138,9	125	21	3
7	1	26,9	131,4	115	26	20
7	1	21,7	125	122	25	12
7	1	24,9	129,2	123	25	4
7	1	24,7	128,1	130	31	8
7	1	29,6	136,2	110	19	10
7	1	27,5	131,2	120	21	9
7	1	28,4	130,2	124	24	14
7	1	22,8	124,8	125	15	8
7	1	26	124,5	130	41	16
7	1	25,8	125,5	117	16	11
7	1	22,9	124,2	130	22	10
7	1	29,1	132,8	105	25	7
7	1	32,1	138,3	115	23	8
7	1	24,1	127,5	120	4	6
7	1	31,6	136,5	120	20	7
7	1	23,5	122,5	120	26	12
7	1	22	131,8	78	14	5
7	1	24,2	129,8	100	32	5
7	1	30,5	134,6	114	23	3
7	1	24,1	125,5	140	32	20
7	1	23,4	122,2	108	23	4
7	1	22,8	128,1	110	24	1
7	1	25,2	124,5	100	28	8
7	1	24,7	126,5	147	35	27
7	1	26,7	135,5	100	16	5
7	1	39,1	129,5	89	16	10
7	1	50,1	146,2	88	20	4
7	1	44,7	143,8	90	28	3
7	1	43,7	135,7	100	32	8
7	1	26,1	119,4	82	28	2
7	1	32,1	132,4	109	24	6
7	1	33,6	131,9	100	22	10
7	1	34,3	129,9	106	24	5
7	1	34,5	134,8	103	23	2
7	1	33,6	134,2	67	12	2
7	1	38,9	141,5	84	11	7
7	1	21,6	119,2	108	23	2
7	1	28	133,6	99	23	14
7	1	24,9	126	91	21	13
7	1	24,4	121,9	128	30	10
7	1	22,5	132,1	85	19	5
7	1	25,3	132	92	20	10
7	1	25	122,1	93	21	4
7	1	25,5	130,5	120	18	3
7	1	22,3	122,2	120	18	7
7	1	21,8	122,5	120	29	8

7	1	18,4	112,1	78	22	6
7	1	20,9	125,8	95	20	5
7	1	24,4	127,4	140	37	9
7	1	27,8	125,1	120	28	7
7	1	24,2	121	105	25	2
7	1	26,2	123,9	113	31	9
7	1	22,7	118,3	95	27	4
7	1	24,4	122	104	24	10
7	1	23,4	124,5	84	10	2
7	1	23,3	133,1	127	29	13
7	1	28,1	138,3	80	24	5
7	1	25,5	128,5	119	23	3
7	1	25,2	133,8	140	21	6
7	1	30,7	133,2	135	23	6
7	1	28	128	115	33	11
7	1	18,6	120,4	110	32	4
7	1	28,7	132	133	20	2
7	1	23,4	128	85	26	15
7	1	31,4	137,5	88	23	6
7	1	28,2	131,5	106	22	3
7	1	32	133,6	102	23	5
7	1	22,8	124,5	101	30	11
7	1	20,9	115,5	95	30	12
7	1	29	136	110	11	7
7	1	34,1	145,1	85	8	4
7	1	23,5	132,6	104	16	12
7	1	24,8	126,1	132	25	8
7	1	26,4	127,1	110	31	5
7	1	28	134,2	125	17	12
7	1	31	141	106	25	1
7	1	26,3	130,1	112	29	12
7	1	21,8	128,9	125	22	7
7	1	24,2	133,1	142	28	6
7	1	23,4	123,5	133	33	1
7	1	21,6	126,1	116	17	7
7	1	25,8	131,1	114	29	12
7	1	31,7	139	150	17	13
7	1	29,2	128,2	120	22	12
7	1	22,7	124	124	35	16
7	1	21,8	118,5	130	30	8
7	1	27,4	124,2	142	27	13
7	1	25,3	126,2	104	22	6
7	1	19,7	121,3	110	17	7
7	1	17,1	116	131	29	9
7	1	35,9	124,2	83	34	13
7	1	33	124,8	105	31	9
7	1	50,4	144	90	12	5
7	1	46,2	137,8	105	17	5
7	1	37,3	130,6	94	28	1
7	1	27,6	123,1	131	28	5

7	1	30,2	125,9	116	21	7
7	1	33,2	133,5	144	17	6
7	1	33,2	134,8	100	24	8
7	1	35,7	130,2	84	17	1
7	1	32,1	124,9	104	31	6
7	1	34,2	133,6	102	18	7
7	1	34	131,1	101	23	2
7	1	23,9	124	110	26	10
7	1	20,9	118,2	110	28	5
7	1	25,7	133,6	101	18	7
7	1	22	125,2	98	19	6
7	1	22,9	122,4	90	19	2
7	1	29,6	138	89	21	8
7	1	30,1	133,7	110	24	8
7	1	23,4	120	130	25	12
7	1	24,1	126,1	78	23	10
7	1	20,7	122,2	107	20	5
7	1	21,9	124,2	119	28	11
7	1	26,8	127,8	74	25	2
7	1	24,7	127,1	74	15	2
7	1	21,2	124,9	90	24	6
7	1	24,7	121,5	104	30	18
7	1	26,9	129,7	120	23	9
7	1	25,7	128,3	90	26	3
7	1	26,7	128,3	106	24	2
7	1	21,8	118,9	113	27	6
7	1	22,8	117,6	122	29	8
7	1	20,3	123,2	122	27	8
7	1	21,4	126	107	33	7
7	1	31	135,9	109	29	6
7	1	20,2	123,2	138	28	6
7	1	20,2	121,5	111	22	12
7	1	22,9	127	125	24	19
7	1	25,9	132,3	153	28	13
7	1	25,2	129,9	110	26	3
7	1	22,7	131,5	132	27	13
7	1	46,8	134,3	122	30	10
7	1	30,5	127,6	90	18	6
7	1	21	117	114	33	9
7	1	29,2	133,5	157	24	8
7	1	20,2	118,2	118	23	15
7	1	26	134,5	100	27	8
7	1	20,6	120,4	120	22	10
7	1	26,2	128,5	140	28	10
7	1	20,6	116,4	98	24	1
7	1	23,5	126,1	97	29	12
7	2	31,3	131,9	119	28	10
7	2	27,3	124,8	101	27	10
7	2	26,1	130,7	106	31	10
7	2	30,7	129	90	32	6

7	2	24,2	121,2	77	31	10
7	2	19,4	123,2	89	30	6
7	2	23,9	123,9	115	29	10
7	2	23,2	127,1	85	26	3
7	2	18,8	110	108	21	4
7	2	23,5	126	95	30	2
7	2	27,4	128,4	79	33	14
7	2	22,4	126	70	19	3
7	2	26,9	124,3	114	34	10
7	2	29,3	129,4	98	37	6
7	2	24,9	131	136	32	8
7	2	22,3	124	68	26	4
7	2	21,7	122,6	111	32	14
7	2	21,4	123,3	94	24	8
7	2	22	124,3	119	35	5
7	2	50,8	145	80	15	3
7	2	32,9	132,1	80	21	3
7	2	27,7	123,9	74	18	1
7	2	38,8	136	90	32	10
7	2	16,2	117,9	88	13	3
7	2	20,7	120,6	59	22	1
7	2	25	125,7	92	29	12
7	2	22,5	126	91	24	4
7	2	24,6	122,8	82	31	4
7	2	27,2	130,4	80	20	1
7	2	26,3	131,2	86	27	4
7	2	24,4	125,6	107	33	15
7	2	20,8	123,8	95	23	2
7	2	21,8	121,1	107	23	7
7	2	27,4	135,1	102	20	5
7	2	22,7	128,1	124	22	8
7	2	20,1	117,7	80	28	10
7	2	25,8	125,2	111	21	6
7	2	21,1	119,2	100	28	5
7	2	26,5	130,8	91	29	7
7	2	24,3	129	89	30	6
7	2	18	117	58	33	13
7	2	18,7	118,5	113	20	4
7	2	20,7	120,1	103	22	7
7	2	24,8	126,1	110	23	7
7	2	26	126,5	100	34	12
7	2	28,6	128,1	82	28	4
7	2	28,2	127,1	80	22	4
7	2	21,4	115,8	90	32	5
7	2	26	124,7	64	26	5
7	2	23,8	130,1	76	7	5
7	2	29,8	130,9	118	27	1
7	2	28,4	124,9	128	36	15
7	2	21,3	117,4	90	30	5
7	2	25,4	122,8	110	20	3

7	2	24,2	121,5	90	24	4
7	2	27,2	126	106	19	10
7	2	21,2	126,5	85	26	5
7	2	29,3	128	125	34	9
7	2	32,8	138	100	35	5
7	2	28,1	134,8	90	21	6
7	2	25	129,3	80	29	7
7	2	20,9	124,9	89	10	7
7	2	23,4	120,5	102	23	4
7	2	21,7	119,5	103	17	16
7	2	28,6	136,5	95	28	5
7	2	30,4	131,2	115	32	5
7	2	32,5	134,2	140	32	20
7	2	27,7	128,2	80	33	3
7	2	19,8	123,8	98	30	5
7	2	27,3	124,1	75	19	3
7	2	21,8	121,2	97	35	5
7	2	24,6	124	72	25	1
7	2	22,7	122,7	90	24	10
7	2	25,1	128,2	116	18	6
7	2	22,9	122,6	90	22	7
7	2	24,8	127,1	145	34	22
7	2	25,2	123,5	123	28	8
7	2	25,3	129,1	98	11	5
7	2	29,9	118,3	75	32	10
7	2	47	135,4	62	31	2
7	2	52,9	140,1	90	24	6
7	2	34	122,7	70	31	2
7	2	51,2	137	65	17	2
7	2	41,2	137	102	30	5
7	2	45,7	131,2	84	13	5
7	2	44,5	140,3	80	19	7
7	2	48,3	145,3	75	18	4
7	2	52,4	140,9	90	27	7
7	2	33,4	131,5	78	20	2
7	2	30,6	124,6	81	21	3
7	2	31,9	123,5	83	30	6
7	2	35,1	136,2	91	31	1
7	2	33	125,4	98	23	7
7	2	32,8	131,5	80	14	20
7	2	30,4	120	70	27	10
7	2	40,1	140,9	100	24	3
7	2	31,3	129,8	68	30	1
7	2	20,9	119,9	108	24	3
7	2	21,5	124,1	89	30	5
7	2	21,1	117,2	101	19	2
7	2	27,2	127,2	71	30	3
7	2	24,9	121,5	90	21	7
7	2	23,7	129,2	90	30	13
7	2	20,8	126,9	102	30	4

7	2	26,2	121	94	28	3
7	2	22,2	114,2	58	21	3
7	2	24	119,7	90	28	5
7	2	27,6	128,9	69	20	5
7	2	27,2	127,9	108	11	8
7	2	18,9	116,8	85	31	5
7	2	23	123,4	85	22	4
7	2	19,2	117,6	87	22	7
7	2	20,9	117,3	92	14	9
7	2	19,2	118	88	26	8
7	2	23	129,1	100	25	3
7	2	21,5	125,1	79	23	1
7	2	31	135	105	17	11
7	2	26,7	132,5	95	30	8
7	2	20	124,8	120	27	11
7	2	25,2	125,6	98	25	5
7	2	22,5	122	103	20	19
7	2	21,3	117,8	90	21	6
7	2	22,7	125,1	81	25	3
7	2	27,5	128,2	97	26	5
7	2	24	123,2	84	25	6
7	2	31,1	132,1	92	25	7
7	2	22,7	119	115	19	9
7	2	22	127	109	24	6
7	2	28,1	125,2	103	34	10
7	2	23,2	127	73	27	10
7	2	28,4	130	131	31	10
7	2	19,3	116	80	26	9
7	2	26,5	128,2	80	28	1
7	2	22,4	119	87	25	5
7	2	23,7	133,5	106	24	2
7	2	24,9	126,5	140	31	7
7	2	26,3	125,6	101	40	1
7	2	23,7	123,6	101	31	20
7	2	27,3	126	110	21	10
7	2	23,7	118,7	90	33	10
7	2	21,5	124,3	102	18	7
7	2	30	130,2	90	31	8
7	2	28,2	135	124	32	5
7	2	26,8	130,2	94	27	9
7	2	26,4	131,3	146	8	5
7	2	25,7	127,5	103	27	9
7	2	26,3	127,2	97	36	8
7	2	23,6	125,4	118	23	10
7	2	22,1	118	114	35	10
7	2	24,9	123,1	85	25	5
7	2	24,5	129	83	26	10
7	2	29	137	132	46	5
7	2	26,8	128,2	98	28	7
7	2	25,3	122	75	27	15

7	2	26,2	125,3	112	23	13
7	2	29,8	119	108	32	10
7	2	37,5	130,9	91	19	4
7	2	47,6	138,7	74	4	2
7	2	54,8	141,1	70	25	8
7	2	32,2	128,6	90	32	3
7	2	30,5	124,6	102	26	4
7	2	30,7	128,2	120	30	7
7	2	30	126	89	26	1
7	2	29,4	125,2	93	24	16
7	2	31,2	125,2	95	26	4
7	2	30,3	124	107	29	6
7	2	36,9	137	115	19	2
7	2	30,2	127,1	99	26	4
7	2	34,5	131,8	100	33	8
7	2	39	136,4	104	23	2
7	2	22,8	121,8	78	27	5
7	2	18	111,5	75	23	13
7	2	16,9	111,9	92	24	6
7	2	17,7	112,2	72	24	3
7	2	21,3	117,4	84	33	8
7	2	20,6	113,5	123	31	8
7	2	25,6	124	80	32	1
7	2	23,3	126	125	35	3
7	2	23,4	123,3	75	23	1
7	2	21,3	125	70	34	3
7	2	28,9	125,5	98	28	8
7	2	23,2	124,2	107	30	4
7	2	31,3	131,7	95	27	6
7	2	24,2	130,6	129	25	13
7	2	27,7	131,3	129	25	10
7	2	25,8	132,1	100	24	7
7	2	20,4	121,5	100	28	3
7	2	19,6	123,2	105	18	4
7	2	48,1	138,1	88	14	2
7	2	29,6	124,5	91	26	8
7	2	29,4	126,2	86	27	1
7	2	31	122	80	31	10
7	2	31,5	130,5	135	30	11
7	2	37,5	139,2	83	32	1
7	2	28,4	124,5	83	24	16
7	2	18,9	112,6	72	28	3
7	2	19	114,1	100	22	3
7	2	25,3	128,5	100	32	10
7	2	23,9	127,5	108	23	6
7	2	22,1	128,1	94	31	10
7	2	26,8	130	90	36	6
7	2	20,3	120	123	26	13
7	2	44,8	136,8	100	32	5
8	1	22,1	126,1	108	17	4

8	1	30,1	131,5	118	22	5
8	1	24,4	129,5	117	23	11
8	1	33,8	148,2	138	24	15
8	1	29,2	135,6	116	12	5
8	1	27	132,1	132	19	12
8	1	48,1	134,6	68	25	1
8	1	54,2	146	108	24	9
8	1	31	126,5	113	22	2
8	1	38,2	135,5	111	16	13
8	1	21,7	123,8	123	24	14
8	1	31,8	131,6	125	20	5
8	1	24,6	129	126	23	11
8	1	23,9	127	146	28	5
8	1	31	135	105	24	8
8	1	24,2	128,5	125	15	7
8	1	33,1	137	100	28	4
8	1	30,3	142,7	160	33	14
8	1	34,4	141,2	129	18	10
8	1	25	139,5	120	19	12
8	1	29	133,3	122	28	7
8	1	30,2	142,5	101	18	5
8	1	36,7	151,1	134	23	4
8	1	29,8	137,5	105	10	2
8	1	33,3	133,8	86	25	4
8	1	31,8	129,6	125	34	15
8	1	38,6	144	100	31	4
8	1	34	142,1	128	18	8
8	1	27,3	133,5	133	23	12
8	1	33,8	136,3	117	28	6
8	1	31,3	132	148	30	13
8	1	45,9	135,2	130	32	2
8	1	53,2	143,5	118	9	7
8	1	56	152,8	107	28	2
8	1	31,5	127,8	119	25	7
8	1	37,3	134,1	120	12	7
8	1	45,1	147,5	142	19	3
8	1	38,7	140,8	144	23	7
8	1	39,2	137,5	104	19	6
8	1	43,9	142,5	108	21	2
8	1	39,8	136,5	141	30	5
8	1	46,6	144,5	116	30	3
8	1	42,2	142,1	120	20	3
8	1	31,6	131,3	120	25	9
8	1	30,7	138,3	127	18	14
8	1	22,6	132,2	75	17	4
8	1	25,4	128,2	136	22	20
8	1	19,7	122,1	105	19	9
8	1	21,3	118,5	81	15	6
8	1	27,8	134,3	142	27	10
8	1	22,2	128	108	30	1

8	1	30,8	135,8	143	22	7
8	1	31	135	112	19	7
8	1	25,2	127,1	110	8	8
8	1	21,8	129,4	134	26	3
8	1	24,1	127,8	120	30	3
8	1	24,7	130,8	138	33	8
8	1	35,4	139,5	116	20	1
8	1	27,2	128,9	135	27	24
8	1	36,5	142,2	127	22	15
8	1	29	127,1	120	26	10
8	1	24,8	125	143	28	12
8	1	28,7	127	126	9	15
8	1	26	130,5	138	27	7
8	1	30,5	134,9	122	22	20
8	1	25,2	129,9	91	22	4
8	1	23,2	121,1	142	35	21
8	1	36	140,8	100	21	4
8	1	24,2	127	134	21	20
8	1	26,2	132,1	123	25	12
8	1	27,3	134,9	105	25	5
8	1	33,2	135,8	132	35	15
8	1	24,8	126,8	110	21	10
8	1	26,4	131,2	155	33	14
8	1	30,1	135,2	132	15	11
8	1	30,6	128	116	34	18
8	1	24,7	134,2	115	20	6
8	1	28,6	135,2	149	23	7
8	1	35	137	128	23	15
8	1	25,2	124	143	40	8
8	1	26,8	136,1	120	28	7
8	1	25	127	142	26	5
8	1	34,5	135,8	121	20	5
8	1	35,5	138	128	31	4
8	1	30,3	141,2	102	24	15
8	1	24,3	126	131	25	24
8	1	27,3	134,3	152	11	9
8	1	36,9	147	73	18	6
8	1	22,5	125,5	100	21	7
8	1	34,2	138,5	102	34	14
8	1	26,2	124,8	137	21	10
8	1	32,6	131	104	13	7
8	1	27,8	131,8	145	30	12
8	1	26,1	128,9	130	23	3
8	1	28,5	133,5	120	20	8
8	1	30,2	133,6	128	20	12
8	1	28,7	137,2	164	21	8
8	1	47,1	135	96	16	1
8	1	52,9	139,6	116	18	5
8	1	45,5	133	133	27	30
8	1	38,4	142,1	108	16	4

8	1	42,7	140,2	132	18	7
8	1	37,6	140	95	26	1
8	1	37,2	134,4	92	21	3
8	1	39,3	134,3	122	18	6
8	1	35,1	133,1	96	16	4
8	1	40,2	140,6	142	29	20
8	1	37,4	138,5	121	19	4
8	1	42,8	139	124	41	1
8	1	41,3	140,8	146	30	6
8	1	39,9	141,8	150	23	10
8	1	42	145	135	18	6
8	1	37,6	139,4	138	24	12
8	1	25,2	129	105	29	8
8	1	37,7	142,5	169	12	4
8	1	27,5	126,8	123	12	24
8	1	20,1	120	105	29	11
8	1	18,5	124	110	31	9
8	1	38,7	145,9	120	20	4
8	1	23,2	127	128	21	3
8	1	22,2	121,8	121	24	10
8	1	28,8	132,6	130	29	12
8	1	27,2	130,2	117	22	4
8	1	25,7	136,3	112	11	6
8	1	20,9	126,8	100	29	3
8	1	31,5	140	101	13	6
8	1	26,9	135,5	141	21	11
8	1	27,4	131,5	110	22	4
8	1	22,5	130,4	102	12	5
8	1	26,9	130,3	117	29	12
8	1	25,2	125	83	21	15
8	1	26,5	129,8	130	37	15
8	1	37,5	143,2	101	21	2
8	1	32,5	132,1	135	24	2
8	1	26,5	134,9	131	25	20
8	1	29,5	135,4	154	30	17
8	1	30,4	137,5	119	28	7
8	1	29	135,3	122	31	6
8	1	25,6	130	152	23	12
8	1	29,8	140,5	146	27	10
8	1	35	138	131	26	6
8	1	26,4	133	140	29	8
8	1	24,4	129,1	130	30	20
8	1	26,4	131,5	140	18	7
8	1	23,5	118	124	30	2
8	1	28,3	136,2	121	19	8
8	1	36,5	142,8	153	26	8
8	1	33,2	141,2	120	25	8
8	1	27,7	129	123	22	1
8	1	32,6	139,9	117	26	5
8	1	29	133,2	110	14	9

8	1	28,4	132	120	27	10
8	1	31,7	137	120	26	10
8	1	24,3	129,3	144	26	10
8	1	30,7	133,1	134	24	2
8	1	20,4	118,5	132	25	12
8	1	23,4	128,2	90	25	12
8	1	26,7	131,5	78	26	13
8	1	23,7	124,9	115	31	13
8	1	40	131,5	111	17	2
8	1	50,8	145,4	90	24	8
8	1	50,3	144,2	120	20	8
8	1	43,7	133,9	110	20	2
8	1	32,4	127,5	59	2	2
8	1	36,9	140,1	110	11	4
8	1	35,4	133,4	130	25	13
8	1	37,1	136,2	105	17	3
8	1	41	136,6	99	30	1
8	1	35,9	131,5	115	27	3
8	1	38,2	136,7	113	20	6
8	1	44,8	144,6	114	16	3
8	1	35,8	132,5	129	31	14
8	1	43	148,5	158	28	6
8	1	29,1	130,2	119	30	8
8	1	29,3	131,7	105	19	12
8	1	25,7	127	130	22	12
8	1	19,8	117	110	26	7
8	1	27,1	131,5	135	25	3
8	1	23,3	123,1	90	33	5
8	1	31,6	138,3	106	15	8
8	1	23,7	132,1	94	23	3
8	1	27,7	132	120	27	2
8	1	27,6	137,5	102	26	5
8	1	32	139,6	116	16	12
8	1	24,4	124	105	25	20
8	1	30,3	135,6	106	21	8
8	1	25,2	134,6	114	24	6
8	1	25,7	130	136	22	10
8	1	23,6	132	154	21	10
8	1	28,8	132,3	120	24	4
8	1	27,2	134,2	110	27	4
8	1	27	131,5	150	29	30
8	1	28,2	131,1	95	32	4
8	1	24,9	131,9	160	24	10
8	1	31	138,4	131	34	4
8	1	25,6	126,3	125	30	6
8	1	24,8	129,4	142	31	14
8	1	28,5	130,5	131	28	12
8	1	28,2	135	135	16	8
8	1	26,8	131,1	110	24	6
8	1	27,1	128	126	25	10

8	1	28,3	135,9	83	30	5
8	1	42	135,1	122	28	10
8	1	35,1	130,6	115	22	7
8	1	33,2	129,8	138	29	31
8	1	38,7	133,2	125	22	6
8	1	38,9	144,2	110	22	8
8	1	30,5	133,7	108	20	3
8	1	25,3	133	161	25	20
8	1	36,2	131	80	21	2
8	2	24,2	132,1	78	28	1
8	2	21,2	127,2	101	34	6
8	2	26	131,5	103	20	8
8	2	35,7	136,2	115	27	8
8	2	30,1	127	125	28	10
8	2	31,1	133	139	24	8
8	2	23,2	122,5	80	19	2
8	2	22,7	131,1	130	28	7
8	2	27,3	135,2	110	24	6
8	2	27,9	128,3	85	13	3
8	2	27,8	135	126	40	6
8	2	31,8	137	98	18	7
8	2	25,7	132,7	140	29	12
8	2	29,2	133,9	120	29	13
8	2	29,3	135,3	108	27	5
8	2	28,1	143,9	105	19	8
8	2	28,7	133,5	102	25	5
8	2	28	138,7	60	32	15
8	2	22,4	131,1	134	29	9
8	2	41	132,5	90	21	10
8	2	38,6	129	85	34	5
8	2	33,5	133,2	110	17	4
8	2	37,4	137,6	76	24	2
8	2	39,1	137,6	126	16	6
8	2	35	128,9	118	30	24
8	2	23,7	125,9	96	20	5
8	2	26,2	139,8	115	30	10
8	2	27,1	134,8	116	29	8
8	2	30,7	132,5	111	33	4
8	2	21,8	128,2	105	27	2
8	2	24	123,1	97	32	10
8	2	22,3	122,1	102	21	5
8	2	21	127,3	88	24	6
8	2	20,4	139,6	120	20	1
8	2	27,2	142,2	114	27	4
8	2	24,1	125,1	117	25	6
8	2	23,6	121,5	93	27	2
8	2	31,4	131,5	104	25	4
8	2	28,2	123,1	90	29	8
8	2	30,8	129,5	158	28	11
8	2	29,5	132	132	27	1

8	2	31,9	132	102	23	4
8	2	24,4	133,1	69	22	6
8	2	29,2	132,2	86	14	5
8	2	27,3	137,6	133	21	8
8	2	29,2	130,6	90	29	7
8	2	27,8	129,8	118	31	11
8	2	25,9	130,1	91	19	2
8	2	27,3	129,3	78	20	5
8	2	28,3	128,1	80	24	5
8	2	26,5	134	78	28	2
8	2	35,7	137,3	101	30	12
8	2	31,3	143	139	28	21
8	2	30,3	132,5	83	15	3
8	2	26,1	134,4	129	23	24
8	2	35,9	138,9	132	24	8
8	2	30,9	137,4	131	23	10
8	2	20,4	126,1	120	32	23
8	2	27,5	129,8	82	22	8
8	2	19,1	118,1	90	27	10
8	2	29,4	134,1	138	33	12
8	2	20,5	128,6	116	28	4
8	2	27,3	140,6	110	20	10
8	2	27	134,9	112	13	5
8	2	26,2	125,3	100	28	7
8	2	23,8	127,3	116	26	10
8	2	33,4	136,9	144	18	15
8	2	26,2	138,6	104	26	8
8	2	23	122,1	103	23	4
8	2	39,1	143,5	105	16	4
8	2	24,5	118	111	36	12
8	2	31,1	131,2	102	31	14
8	2	43,1	134	80	28	13
8	2	40,7	132	100	25	4
8	2	36,3	126,9	105	16	3
8	2	43,7	135,6	78	24	7
8	2	57,5	136	85	35	4
8	2	52,3	148,2	94	35	6
8	2	33,5	131,5	118	21	1
8	2	36,4	136,5	90	26	3
8	2	41,8	148,4	108	19	4
8	2	34,7	133,2	127	30	12
8	2	43,6	143,1	126	29	7
8	2	37,5	136	135	35	8
8	2	31,8	127,5	88	18	4
8	2	36,5	134,8	112	25	6
8	2	34	129,8	100	36	12
8	2	36,8	138	123	31	16
8	2	42,3	138	105	17	6
8	2	33,8	127	100	33	4
8	2	33	132	90	12	2

8	2	29,5	134,1	113	22	1
8	2	27,2	132,1	102	24	7
8	2	33,7	136	135	25	9
8	2	29,4	140	59	21	2
8	2	31,7	143	81	23	4
8	2	26,7	128,9	68	30	2
8	2	26,8	137	122	23	7
8	2	25,4	130	119	22	10
8	2	27,8	124	70	30	7
8	2	24,1	124,8	119	29	8
8	2	31,6	131,5	131	42	15
8	2	28,7	137,2	109	24	9
8	2	26,9	125,4	114	36	16
8	2	27,1	134,3	133	30	12
8	2	29,5	130,9	70	9	4
8	2	27,6	131,5	125	25	1
8	2	21,2	126	79	23	4
8	2	34,6	139,6	111	32	8
8	2	29,9	136,2	115	25	12
8	2	26,5	128	117	36	9
8	2	26,2	130,8	102	25	12
8	2	21,6	130	110	16	6
8	2	30,6	139,4	93	20	10
8	2	32,3	142,5	122	22	4
8	2	24,6	135	100	25	5
8	2	23,8	126,6	115	29	20
8	2	24,8	134,1	110	22	3
8	2	33,3	137,2	116	20	5
8	2	29,4	139	98	34	4
8	2	18,7	129,2	132	21	5
8	2	27,1	129,5	102	28	5
8	2	32,2	139,1	72	30	6
8	2	23,6	123,6	83	29	4
8	2	26,1	134,5	128	28	20
8	2	28,7	133,1	129	29	7
8	2	22,3	128,3	122	29	7
8	2	26,5	128,6	100	25	1
8	2	28,3	130,6	121	29	10
8	2	35,1	142,6	80	4	1
8	2	25,7	128,5	99	9	2
8	2	28,9	129	126	33	13
8	2	39,9	132,5	60	30	5
8	2	46,6	137,5	60	26	1
8	2	59,5	150,5	93	23	4
8	2	45,9	138	90	25	10
8	2	29,7	120,3	90	26	13
8	2	37,1	130,5	114	35	13
8	2	44,4	141,5	132	19	4
8	2	40	135,5	110	29	2
8	2	39,7	141	87	20	14

8	2	39,2	135,1	123	32	12
8	2	42,1	141	98	25	10
8	2	32,6	134,2	128	30	4
8	2	26,1	131	109	22	14
8	2	24,3	133	115	31	8
8	2	21,9	119,1	120	29	18
8	2	19,4	122	93	24	4
8	2	28,6	129	99	23	8
8	2	25,7	130,6	138	33	11
8	2	28,3	130,5	80	15	8
8	2	32,1	130,8	104	23	7
8	2	21,8	129,3	80	25	1
8	2	22,3	129,6	123	25	18
8	2	35,7	139,4	73	8	1
8	2	28,6	133,2	80	25	10
8	2	22,7	124,9	137	27	13
8	2	25,5	126,6	122	19	7
8	2	24,5	126,5	107	26	7
8	2	29,4	136,1	128	20	5
8	2	20,7	120,3	90	10	6
8	2	41,1	154,4	120	28	7
8	2	28,1	135,4	119	30	5
8	2	46,4	137	96	19	1
8	2	60,5	143,5	78	29	5
8	2	31,8	127,5	110	19	5
8	2	37,8	137	74	24	2
8	2	29,4	139,4	147	29	12
8	2	34,8	145,2	70	21	7
8	2	24,7	122,6	130	27	5
9	1	37,6	145,3	136	12	6
9	1	25,8	132	150	18	10
9	1	25,4	133	140	18	10
9	1	28,1	131,2	129	26	20
9	1	30,8	133,4	140	22	12
9	1	42,7	141,3	128	25	9
9	1	27,8	137	115	12	16
9	1	27,4	134,6	142	28	12
9	1	30,9	138,7	157	24	16
9	1	20,7	120,8	125	29	4
9	1	41,5	147,1	108	13	3
9	1	28,3	134	165	21	23
9	1	31,6	141,7	124	26	12
9	1	24,2	131	130	19	10
9	1	30,5	144,2	107	23	1
9	1	41,5	149,5	100	26	2
9	1	37,3	144	142	16	10
9	1	36,5	141,5	115	20	10
9	1	29,3	135,5	98	13	7
9	1	32,3	136	123	12	13
9	1	29,6	147,1	125	22	14

9	1	37,8	142,1	155	25	15
9	1	32,4	138,9	140	23	7
9	1	34,3	140,5	150	23	7
9	1	52,6	149,2	88	13	3
9	1	52,8	147,2	90	14	2
9	1	50,7	146,2	118	14	6
9	1	57,3	145,9	75	26	1
9	1	41,2	143,7	116	23	1
9	1	43,2	144	125	19	11
9	1	45,2	142	141	25	15
9	1	47,3	145,5	105	17	3
9	1	39,4	140,5	121	33	10
9	1	52,6	150	101	20	6
9	1	37,2	140,9	115	27	10
9	1	26,6	128,2	105	30	7
9	1	21,9	130,8	120	12	4
9	1	30,6	136,6	106	9	5
9	1	33,6	139,9	145	19	10
9	1	33,8	143,2	145	25	26
9	1	34,2	144,3	157	16	4
9	1	31,2	132,1	137	32	9
9	1	44,2	158,6	153	24	12
9	1	28,2	136,4	99	32	7
9	1	30,8	128,7	122	22	8
9	1	38,9	148,6	135	18	10
9	1	34,7	138	141	30	2
9	1	27,2	130,5	103	15	6
9	1	32	137,6	126	23	8
9	1	32,1	135	143	35	9
9	1	25,1	130	121	16	19
9	1	32,9	138	150	26	7
9	1	34,8	135,5	98	23	3
9	1	35,5	140	125	23	9
9	1	30,4	135,4	128	12	7
9	1	32,6	138,7	150	21	1
9	1	25,5	130,3	139	31	12
9	1	30,4	131,9	148	31	18
9	1	28,8	138,1	152	19	6
9	1	35,5	145	135	20	7
9	1	29,5	144,2	138	23	9
9	1	26,6	139	135	26	5
9	1	32,9	136,2	121	22	10
9	1	41,7	150,2	91	1	3
9	1	28	139,3	147	27	24
9	1	25,7	132,1	130	21	18
9	1	39,4	145	150	19	7
9	1	36,3	139	152	26	9
9	1	39,3	142,2	139	24	16
9	1	26,2	132,1	140	25	15
9	1	36,7	147,8	140	16	15

9	1	28,1	138,6	138	29	6
9	1	32,8	141,8	129	28	10
9	1	24	132,4	125	16	12
9	1	29,5	144	120	10	12
9	1	27,4	143	138	13	12
9	1	28,3	136,2	110	16	9
9	1	33,1	144,5	135	21	14
9	1	27,6	139,2	150	27	23
9	1	35,2	143,8	140	15	6
9	1	34,3	132,5	142	30	16
9	1	37,9	141,4	174	24	18
9	1	35,3	144,2	158	23	8
9	1	25,1	134,3	160	36	8
9	1	37,6	143,1	145	22	5
9	1	25,2	131,6	128	19	12
9	1	29,5	141,3	142	25	9
9	1	30,1	146,1	119	26	7
9	1	29,2	142,9	114	13	3
9	1	26,2	128,9	138	29	7
9	1	35,5	140,6	143	33	25
9	1	26,4	133	142	25	21
9	1	37,8	144,3	105	32	8
9	1	26,3	128,5	80	20	6
9	1	29,8	140,1	93	17	7
9	1	28,9	141,5	110	5	4
9	1	19,2	125,8	93	21	7
9	1	27,3	132,9	148	19	4
9	1	32,3	145,2	143	15	9
9	1	35,5	147,9	140	14	3
9	1	36,1	142,6	135	34	24
9	1	29,1	129	123	19	9
9	1	32,6	138,5	130	20	14
9	1	30,3	141	150	11	8
9	1	28,7	132,2	119	22	10
9	1	40,4	147,3	112	13	6
9	1	41,2	132	96	19	8
9	1	46,2	133,1	102	28	10
9	1	49,7	144	92	34	4
9	1	50,2	139,8	120	31	20
9	1	54,1	147,8	113	22	3
9	1	56,7	149,5	114	20	7
9	1	46,4	136,6	137	26	15
9	1	72,5	156,3	100	20	4
9	1	60,2	144,8	74	24	1
9	1	46,4	149,5	122	14	5
9	1	40,7	143,5	144	17	5
9	1	43,2	144	130	29	12
9	1	43	140	137	25	4
9	1	46,6	148	125	11	8
9	1	38,8	133,5	133	27	22

9	1	45,7	143,5	126	18	10
9	1	37,5	137	91	20	10
9	1	45,6	145,1	107	14	3
9	1	39,2	137,2	115	16	5
9	1	52,4	156,1	140	24	5
9	1	28,7	136,4	90	18	10
9	1	29,2	139,1	136	22	13
9	1	26,5	136,1	128	21	8
9	1	36,3	136,7	117	27	10
9	1	29,9	139,4	124	20	18
9	1	26,9	133,1	100	17	5
9	1	26,1	129,5	153	32	13
9	1	27,1	135,6	136	30	7
9	1	42,7	148,8	110	16	7
9	1	26,4	124,7	112	23	9
9	1	32,5	131,1	128	27	4
9	1	35,5	147,2	167	33	25
9	1	33,1	141,2	139	26	5
9	1	32,2	138,9	138	25	10
9	1	36	142	130	27	9
9	1	33,9	134,6	124	29	20
9	1	36,4	141,2	110	23	18
9	1	33,1	144,1	120	32	7
9	1	27,8	133,5	139	34	25
9	1	21,1	126,1	104	25	11
9	1	35,1	138,1	130	11	2
9	1	41,6	150,3	162	30	21
9	1	38,8	142	149	23	10
9	1	31,6	139,5	132	27	14
9	1	24,3	135,3	134	18	7
9	1	32,6	141	121	22	4
9	1	31,3	130	140	31	19
9	1	41,6	149,5	137	22	10
9	1	29,4	131,7	83	32	1
9	1	27,9	136,9	148	27	8
9	1	24,8	129,5	120	27	10
9	1	38,3	140,2	105	24	3
9	1	26,7	131,9	136	23	8
9	1	33,6	139,9	120	16	10
9	1	30,7	136,5	107	22	13
9	1	38,8	155,4	160	24	10
9	1	29	139,2	129	18	11
9	1	29,9	138,7	143	22	5
9	1	35,2	140,5	132	18	5
9	1	30,2	131,4	150	24	20
9	1	31,6	134,8	140	35	3
9	1	28,9	137,8	157	36	42
9	1	31,9	142,3	140	26	21
9	1	27,6	135,8	118	30	10
9	1	34,1	143,5	130	9	8

9	1	26,9	136,8	160	28	5
9	1	41,6	150	165	30	21
9	1	26,5	137,1	110	17	10
9	1	38,3	147,9	116	24	7
9	1	26,2	134,7	123	27	4
9	1	32,9	141,1	120	15	8
9	1	34,1	139,7	162	26	20
9	1	30,6	148	152	30	4
9	1	26	126,9	134	30	10
9	1	41,2	143,1	110	13	15
9	1	33,9	134	158	18	28
9	1	29,7	130,6	137	22	12
9	1	34,6	138,2	120	26	2
9	1	23,8	127,2	135	29	12
9	1	34,3	147	172	29	8
9	1	43,3	135,5	115	22	3
9	1	59,5	154,2	145	16	5
9	1	66,5	144	90	18	3
9	1	60,6	150	112	20	6
9	1	60,1	150	90	2	3
9	1	67	153	110	10	1
9	1	46,7	138,1	112	23	4
9	1	40,9	141,5	118	33	15
9	1	46,7	144,3	71	12	5
9	1	40,5	139,5	130	19	12
9	1	47,5	152,3	138	26	11
9	1	44,6	140,4	110	22	7
9	1	42	143,6	105	1	8
9	1	41,8	144,2	104	10	18
9	1	41,8	136,2	101	27	21
9	1	19,9	128,2	136	21	7
9	1	27	133	135	13	3
9	1	29,9	134,9	110	28	5
9	1	28,8	134,8	137	18	10
9	1	35	150	101	11	14
9	1	28,6	132,5	160	32	15
9	1	28,6	134	158	35	13
9	1	30,1	146,1	110	30	4
9	1	23,4	125,3	140	23	10
9	1	31	141	70	12	1
9	1	28,7	143,1	135	23	13
9	1	26,9	129,4	154	24	10
9	1	36,5	141	147	31	12
9	1	30,9	141,1	140	33	12
9	1	29,5	133	143	23	10
9	1	25,3	133,8	121	19	7
9	1	68,4	140	106	29	2
9	1	39,3	136,2	80	17	1
9	1	45,8	145,9	102	18	9
9	1	25,4	134,2	145	24	15

9	1	21,7	121,1	140	19	23
9	1	34	133,7	120	25	7
9	1	25,5	132,3	159	20	8
9	1	61,2	147,5	102	14	9
9	1	70,6	151,7	100	23	1
9	1	47	146,6	115	28	7
9	2	35,8	141,8	139	32	6
9	2	42,2	149,9	83	12	2
9	2	32,3	148,5	120	23	4
9	2	31	135,3	133	33	9
9	2	35,8	151,5	102	26	8
9	2	30	135,3	118	33	9
9	2	35,7	146,2	112	16	8
9	2	28,5	133,2	84	17	4
9	2	38,2	144,2	106	7	8
9	2	41	139,5	108	22	9
9	2	48,3	151,5	122	9	5
9	2	31,2	136,4	112	19	7
9	2	21	120	84	30	12
9	2	24,3	128	138	24	5
9	2	29	135,5	110	18	5
9	2	33,5	138,4	155	26	1
9	2	33,2	143,5	126	19	13
9	2	35,4	143,3	102	26	7
9	2	31,6	143	106	29	8
9	2	35,1	135,9	113	24	15
9	2	35,5	146	94	20	10
9	2	27,6	137,6	101	18	9
9	2	34,7	146,2	83	9	7
9	2	29,3	140	165	24	10
9	2	34,9	145	118	24	8
9	2	25,8	137	110	18	5
9	2	28,4	136,2	123	28	20
9	2	31,2	136,2	112	19	4
9	2	31	134,6	120	29	12
9	2	31,2	136,2	96	30	5
9	2	31,7	133,1	89	15	4
9	2	27,8	138,6	79	32	9
9	2	37,4	145,6	135	32	11
9	2	31,3	142,2	143	25	14
9	2	37,3	144,2	110	23	20
9	2	33,7	145,1	86	6	4
9	2	23,1	126,2	130	32	7
9	2	31,8	141	110	23	6
9	2	34,4	136,9	112	26	9
9	2	30,2	138,2	148	20	6
9	2	57,4	153	93	24	7
9	2	58	148,1	100	20	8
9	2	36,1	133,8	93	25	10
9	2	42,4	142,9	125	16	2

9	2	44,4	141,5	101	22	2
9	2	43,4	145,5	125	31	18
9	2	39,8	134	80	28	10
9	2	28	144,2	136	16	17
9	2	20,2	126,1	121	31	5
9	2	25,1	126	140	29	11
9	2	24,6	130	100	28	13
9	2	34,9	139,5	86	27	4
9	2	26	136,2	119	19	6
9	2	25,1	130,2	105	28	20
9	2	30,5	134,2	123	20	8
9	2	34,8	138,2	98	34	6
9	2	23,3	128,7	121	20	9
9	2	34,8	145,7	128	15	5
9	2	33,9	141,7	100	25	3
9	2	33,3	147,2	118	24	4
9	2	25,6	127,8	113	26	10
9	2	31,8	144,8	120	35	8
9	2	33,7	149	128	22	2
9	2	31,2	142,1	133	33	4
9	2	31	126,8	125	28	34
9	2	27,5	136,9	127	33	5
9	2	24,8	132	125	23	12
9	2	27,7	129,6	122	17	6
9	2	32,4	138	133	26	3
9	2	28,3	140	137	25	7
9	2	28,6	140,2	110	23	2
9	2	27,5	141,2	120	22	6
9	2	36,1	140	129	17	2
9	2	22,7	130,6	141	30	6
9	2	28,7	139	105	29	9
9	2	27,9	134,5	95	36	9
9	2	30,3	139,6	111	32	4
9	2	32	136,3	126	30	12
9	2	37,5	140	124	27	8
9	2	22,8	127,6	98	22	10
9	2	38,8	140,5	72	12	6
9	2	30,4	143,2	100	17	15
9	2	26,6	136,9	130	25	1
9	2	29,6	138,5	116	19	13
9	2	27,7	137,1	143	28	10
9	2	35	138	137	11	15
9	2	35,2	143,9	117	19	9
9	2	31,5	143,3	88	16	10
9	2	28,6	131,2	90	22	5
9	2	29,3	136,5	116	32	4
9	2	44	153,4	134	26	8
9	2	34	141,6	133	11	14
9	2	49,7	142,8	125	28	20
9	2	46,6	137,8	105	25	5

9	2	65	149,7	112	23	1
9	2	44,8	142,8	78	22	2
9	2	34,2	129,4	140	31	4
9	2	32,9	129,1	110	31	1
9	2	39,2	139,5	136	18	10
9	2	38,5	130,9	110	26	3
9	2	40,3	139,5	125	22	11
9	2	43,7	142,5	115	34	9
9	2	49,2	150,1	120	13	16
9	2	50,8	159,6	132	20	15
9	2	42,2	141	76	29	10
9	2	43,7	142,8	90	29	9
9	2	36,7	135	126	32	16
9	2	50,5	145,9	128	37	17
9	2	42,7	165,2	65	16	4
9	2	26,3	138,9	113	17	5
9	2	25,8	134,1	92	29	2
9	2	30,3	144	110	17	6
9	2	30,1	137,5	100	23	6
9	2	34,5	136,8	103	30	6
9	2	22,9	130,3	127	25	9
9	2	29	136,1	128	34	12
9	2	33,7	145,5	103	19	4
9	2	42,3	153,5	83	25	7
9	2	35,2	143	112	21	9
9	2	28,3	138,9	150	28	15
9	2	35,1	142,3	90	17	7
9	2	25,1	132	142	26	13
9	2	29,4	145,6	118	17	17
9	2	32,5	143,2	109	19	3
9	2	33,3	140,7	107	18	6
9	2	23,4	132,1	45	19	8
9	2	33,9	137,9	117	27	22
9	2	33,8	135,1	112	31	25
9	2	25,6	126	105	30	13
9	2	31,1	137	89	16	16
9	2	35,2	149	96	23	7
9	2	28	130	126	21	8
9	2	27,3	138,3	101	22	7
9	2	26,6	131	100	30	3
9	2	24,4	127,6	118	22	7
9	2	31,6	143	120	29	13
9	2	30,3	135,5	65	20	1
9	2	28,7	136,4	90	11	3
9	2	39,8	144,2	113	26	4
9	2	26,2	133,2	142	25	13
9	2	30,2	137,2	130	36	3
9	2	31,5	131,6	120	34	5
9	2	30,5	139	91	30	10
9	2	25,2	144,2	80	30	8

9	2	33,9	144,9	136	23	6
9	2	36	141,9	145	29	8
9	2	38,2	144,9	106	28	10
9	2	33,7	131,4	135	31	16
9	2	26,7	138,9	118	18	10
9	2	32,6	134,8	89	18	3
9	2	22,2	136,8	138	23	6
9	2	24,6	135	119	29	9
9	2	28,1	132,6	110	30	10
9	2	33,5	139,1	138	32	23
9	2	38,7	148,4	110	18	1
9	2	31,2	134,6	104	41	23
9	2	29,5	139,1	126	15	7
9	2	41,1	144,2	115	24	18
9	2	62,2	150	94	33	6
9	2	51,5	142,2	80	29	2
9	2	42,4	137,5	135	26	7
9	2	41,2	144,9	128	25	10
9	2	39,4	141	124	21	3
9	2	38,3	140,1	123	33	5
9	2	48,2	146,6	107	23	3
9	2	41,7	140,5	91	35	14
9	2	48,2	143,2	108	28	3
9	2	44,6	148	140	26	10
9	2	24,4	127,5	122	27	4
9	2	42,4	150,2	159	38	8
9	2	26,4	139,1	112	23	21
9	2	24,5	133,9	135	29	13
9	2	30,5	137,1	100	23	4
9	2	29,9	134,3	127	34	20
9	2	32,9	132,8	112	30	19
9	2	32,9	143,9	125	24	6
9	2	26,8	132,4	117	29	7
9	2	32,4	138,4	108	31	18
9	2	33,6	143,1	135	15	13
9	2	34,5	147,6	117	20	7
9	2	35,1	142,3	107	20	8
9	2	32,2	137	82	22	5
9	2	33,2	133	103	13	6
9	2	29,7	135,5	137	30	15
9	2	38	155,2	104	3	1
9	2	27,7	130,2	135	31	20
9	2	25,2	132,6	133	41	8
9	2	34,3	143,5	183	22	12
9	2	26,2	132,3	122	36	20
9	2	31	133	131	26	14
9	2	28,2	140,7	120	21	10
9	2	27,6	136	119	25	9
9	2	33,8	144	102	25	16
9	2	29,7	138,3	98	20	6

9	2	27,2	136,6	113	27	6
9	2	32,3	147,1	100	18	13
9	2	37,2	137,8	105	34	10
9	2	31,2	138	100	30	3
9	2	24,1	127,2	131	15	3
9	2	39,8	152,5	102	22	4
9	2	19,7	125,2	104	30	4
9	2	31,8	138,5	112	23	6
9	2	36,5	139,2	106	27	9
9	2	24,3	133,9	126	26	8
9	2	37,8	140,1	111	29	6
9	2	26	130,2	118	27	17
9	2	36,5	135,9	118	22	9
9	2	42,5	136,2	148	38	16
9	2	45,8	140,7	99	27	7
9	2	45,7	145	89	13	4
9	2	41,3	144,3	81	25	8
9	2	40,5	139,2	116	21	5
9	2	33,9	140	94	21	3
9	2	33,1	138	109	7	7
9	2	35,8	158,2	142	35	10
9	2	38,9	160,8	99	21	4
9	2	56,2	144,5	104	32	2
9	2	48,5	148,2	113	27	5
10	1	38,2	148,2	130	17	10
10	1	42,3	148,2	136	23	2
10	1	30,7	141,6	128	23	8
10	1	29,8	138	152	31	2
10	1	28,2	132,8	118	20	8
10	1	34,2	141,3	135	21	12
10	1	49,2	155,1	105	26	7
10	1	63,9	160	103	13	3
10	1	45,4	143,9	112	25	8
10	1	47,3	144	141	32	11
10	1	39	148,8	150	20	4
10	1	39,4	148	117	7	4
10	1	33,8	143,5	128	25	11
10	1	34,2	144,8	142	22	6
10	1	42,2	150,3	90	12	4
10	1	44,4	152,2	124	26	10
10	1	39	153,9	146	20	15
10	1	32,5	144	168	32	21
10	1	36,5	142,3	140	18	20
10	1	36	146,1	150	24	11
10	1	31,8	144,1	150	30	15
10	1	40,7	147,3	130	26	8
10	1	28,5	139,2	110	18	5
10	1	43,2	146,8	121	21	4
10	1	31,4	148,1	150	18	9
10	1	29,1	133,8	132	28	15

10	1	36,4	148,5	131	14	8
10	1	39,3	149,5	138	20	18
10	1	29,3	137,5	159	18	19
10	1	35,5	154	125	35	18
10	1	32,2	138,6	119	17	14
10	1	37,6	140,8	112	25	8
10	1	35,1	146,1	147	17	5
10	1	43,9	148,2	130	11	12
10	1	35,7	146,4	131	19	15
10	1	29,9	139,9	130	17	8
10	1	33,2	150,1	119	17	10
10	1	32,2	138,9	140	22	12
10	1	56,5	148,2	109	30	8
10	1	80,1	144,8	120	27	8
10	1	63,2	153,1	120	19	7
10	1	73	155,6	85	21	3
10	1	55,3	151	120	11	5
10	1	48,2	143,8	80	28	5
10	1	47,4	144	121	8	5
10	1	49,7	149,2	126	30	9
10	1	43,1	142,1	125	18	11
10	1	46,9	149,8	134	29	4
10	1	46	145,6	120	1	7
10	1	50,6	145,2	110	6	4
10	1	27,3	127,9	93	12	10
10	1	26,7	129	130	25	5
10	1	33,2	138,4	146	25	10
10	1	32,5	140,6	95	25	2
10	1	31,6	142,1	158	31	10
10	1	24,1	128,1	160	34	18
10	1	35,2	145,2	138	26	7
10	1	31,3	132,6	152	31	15
10	1	35,1	138,2	135	18	13
10	1	34,5	141,2	135	39	12
10	1	33,8	146	143	29	12
10	1	37,6	146	100	25	7
10	1	30,7	142,7	123	29	6
10	1	28,7	140,2	150	7	12
10	1	36,4	142,1	124	21	8
10	1	27,8	134,7	149	16	10
10	1	33	143,1	127	18	12
10	1	33,1	145,4	140,5	29	20
10	1	27,3	135,8	139	27	12
10	1	44,1	158	160	25	14
10	1	30	133	140	25	10
10	1	33,7	136,8	130	36	12
10	1	28,6	138,7	133	23	12
10	1	29,2	136,2	138	19	12
10	1	27,3	137,1	125	22	11
10	1	42,1	147,2	102	25	8

10	1	37,7	146,1	152	30	13
10	1	29,3	144,9	90	16	3
10	1	34,5	145	125	18	12
10	1	31,7	151,3	152	35	9
10	1	27	135,3	125	31	17
10	1	38	147	127	37	13
10	1	31,7	132,3	146	30	17
10	1	33,2	142,6	183	25	20
10	1	30,5	141	155	16	12
10	1	37,5	148,2	120	10	5
10	1	41,8	156,1	180	23	10
10	1	34,2	152,1	122	3	3
10	1	30,9	137	150	35	30
10	1	38,3	152,9	128	13	1
10	1	39,7	142,5	70	28	3
10	1	35,2	146,5	131	23	4
10	1	31,9	138,1	172	32	20
10	1	29,3	142,3	125	23	18
10	1	30,4	132,5	157	17	10
10	1	30,6	130,5	173	27	19
10	1	31,9	145,3	128	15	2
10	1	45,3	149,5	140	34	2
10	1	34,8	135,2	138	24	11
10	1	30,8	140,9	120	29	4
10	1	27,3	134,1	145	21	12
10	1	40,7	153,3	156	16	3
10	1	39,6	153,2	148	29	4
10	1	29,6	137,2	110	18	12
10	1	33,3	147,3	155	24	2
10	1	29,2	138,9	153	23	25
10	1	33,6	141,6	120	20	4
10	1	46,3	153,5	160	22	14
10	1	31	142,5	123	28	1
10	1	32,8	137,5	152	28	6
10	1	33,9	150,1	140	30	31
10	1	30,9	141,8	190	18	23
10	1	36,2	152,5	152	24	18
10	1	32,3	144	128	24	2
10	1	37,3	146,8	160	25	12
10	1	34,4	135,2	106	28	3
10	1	61,3	156,8	93	17	8
10	1	62,7	144	98	11	1
10	1	49,9	139,1	133	24	16
10	1	57,2	147,6	133	13	7
10	1	39,7	139,5	149	18	1
10	1	47,5	153	140	24	15
10	1	46,4	141	130	13	3
10	1	55,9	154,5	112	22	1
10	1	49,2	151,1	136	17	6
10	1	53,3	152,5	145	12	2

10	1	49,3	146,8	120	28	6
10	1	40,2	133,5	112	26	17
10	1	40	138,5	132	16	10
10	1	45,2	145,9	132	21	3
10	1	45,1	142,6	172	26	2
10	1	40,1	138,5	93	20	2
10	1	56,4	154	120	22	1
10	1	45,5	134,9	149	24	15
10	1	47	147,1	120	28	8
10	1	23,7	127,2	108	27	9
10	1	24,2	135	135	12	1
10	1	29,8	140,5	150	22	14
10	1	39,8	164,1	150	18	19
10	1	31,6	140,9	106	20	12
10	1	39,1	147,2	157	18	14
10	1	42,1	147	114	26	4
10	1	25,5	130,6	146	26	25
10	1	35,4	144,6	143	14	12
10	1	32,9	141,5	148	33	10
10	1	36,8	138	128	13	4
10	1	34,1	145,9	161	27	8
10	1	28	135,4	171	29	30
10	1	33,2	137,2	136	31	3
10	1	29,4	133,2	135	16	15
10	1	37,1	147,5	145	28	16
10	1	27,9	133,2	120	24	17
10	1	28,4	133	137	20	16
10	1	34,2	140,2	76	25	30
10	1	24,4	133,2	130	28	10
10	1	41,9	147,3	154	33	6
10	1	24,9	130,5	123	12	8
10	1	34,7	140,1	162	30	26
10	1	34,6	143,5	125	16	10
10	1	32,8	140,6	106	14	3
10	1	30,7	138,1	128	31	12
10	1	31,3	140,3	121	38	12
10	1	29,6	134,2	112	32	6
10	1	28,1	144	127	11	2
10	1	36,2	144,9	140	29	11
10	1	34,1	145,8	150	22	2
10	1	31	132,9	154	25	12
10	1	33,3	152,1	138	23	6
10	1	33,2	141,8	130	16	20
10	1	35,1	136,5	102	31	4
10	1	38,3	153,5	122	20	5
10	1	39,1	149,6	120	17	20
10	1	39,9	149	135	15	12
10	1	33,8	143,8	101	21	11
10	1	27,7	135,5	153	36	10
10	1	34,2	141,6	136	27	23

10	1	26,3	143	167	26	6
10	1	29	143	142	25	12
10	1	33,8	147,3	169	34	20
10	1	26,4	137,9	100	16	8
10	1	34,2	141	180	33	24
10	1	39,6	146,6	132	18	18
10	1	39,2	152,1	125	22	15
10	1	36,3	147	137	18	20
10	1	35,2	136,5	153	24	7
10	1	24,5	132,6	140	15	21
10	1	38,2	148,2	132	17	9
10	1	39,2	141,6	147	20	10
10	1	32	144,2	156	22	18
10	1	32,8	143,8	115	12	9
10	1	55,2	149,7	119	29	11
10	1	72,2	158	98	18	1
10	1	61,1	151	118	23	8
10	1	53,4	142,1	73	25	4
10	1	65,6	152,2	118	28	5
10	1	44,7	146,1	138	25	7
10	1	50,2	148,2	120	29	3
10	1	53,1	152,1	119	25	3
10	1	40,5	139,9	133	23	14
10	1	50,8	156,2	82	14	5
10	1	51,9	151,1	83	22	5
10	1	57,4	152	120	14	7
10	1	43,5	140,8	140	35	3
10	1	51,6	150,1	130	30	5
10	1	42,1	142,1	175	28	25
10	1	56,1	154	126	18	10
10	1	63,7	160,6	140	27	7
10	1	49,8	144,3	152	19	7
10	1	42	145	105	25	5
10	1	31	131,7	109	25	25
10	1	54,6	175,2	120	18	15
10	1	41,5	150,3	128	25	10
10	1	32,7	144,5	118	30	10
10	1	27,4	139,5	173	25	8
10	1	31,4	134	132	16	20
10	1	38,8	150,4	130	28	6
10	1	40,6	146,1	134	26	19
10	1	30,6	137,8	150	20	10
10	1	34,6	146,2	155	23	4
10	1	37,7	138,1	130	20	3
10	1	34,8	143	140	23	35
10	1	26,3	137,7	124	10	9
10	1	29,2	139,1	148	26	17
10	1	48,9	161	122	25	3
10	1	40,5	144,6	127	26	3
10	1	36,7	140,2	148	25	5

10	1	68,5	155,6	120	25	1
10	1	67,5	152,5	100	28	3
10	1	47,8	139,9	101	26	10
10	1	48	149,4	104	20	2
10	1	36,8	143,5	105	22	6
10	1	43,9	150,8	137	25	8
10	1	26,4	125,7	109	25	8
10	1	30,5	135,6	113	27	6
10	1	31,7	145,9	130	23	10
10	1	46,3	147,1	129	18	12
10	2	30,3	135,1	85	14	6
10	2	42,5	146,5	101	22	3
10	2	35,8	156,1	140	26	6
10	2	22,9	134,1	145	41	6
10	2	32,2	144,5	149	32	20
10	2	25,2	129	132	23	26
10	2	31,1	138,6	106	21	6
10	2	39	154,8	150	35	15
10	2	51,4	162,9	100	33	10
10	2	38,2	147,2	100	7	10
10	2	56,9	150,5	125	32	6
10	2	47,9	151,5	107	15	1
10	2	54,4	156	146	34	8
10	2	48,4	145,5	104	26	2
10	2	41,3	155,2	131	31	8
10	2	25,3	134,1	108	26	7
10	2	42,6	149,3	110	11	7
10	2	39,3	147	108	34	11
10	2	32,1	140,2	103	19	2
10	2	30,3	141,9	65	20	2
10	2	34,4	142,5	129	19	1
10	2	34,9	147,7	115	25	9
10	2	44,6	149,8	153	32	12
10	2	38,5	150,8	112	24	10
10	2	27,2	150,8	106	21	10
10	2	28,3	142,2	102	14	15
10	2	31,5	147,4	130	28	8
10	2	31,8	141	131	15	10
10	2	29,7	141,5	127	18	7
10	2	26	143	120	20	4
10	2	43,8	159,5	122	22	2
10	2	35,2	144	112	36	26
10	2	35,4	137	94	32	2
10	2	34,7	145,5	89	22	8
10	2	35,1	141,1	103	16	10
10	2	30,2	141	93	10	6
10	2	48,6	156,2	114	24	10
10	2	37	139,2	130	16	4
10	2	36,8	140,7	92	20	5
10	2	37,5	146,5	121	15	13

10	2	36,1	149,5	127	35	8
10	2	27,6	138	117	22	10
10	2	35,1	149	130	18	6
10	2	36,7	159,3	152	25	13
10	2	44,5	154,2	98	25	3
10	2	23,2	131,2	98	26	6
10	2	56,1	144,1	86	8	1
10	2	42,4	144,5	110	33	9
10	2	46,3	145,1	105	27	7
10	2	47,7	152,5	151	24	20
10	2	46,9	148,4	120	29	11
10	2	48,2	142	68	18	2
10	2	47,2	145	87	28	2
10	2	42	140	123	22	4
10	2	48,1	150,3	114	23	13
10	2	37,2	137,7	104	22	6
10	2	32,8	136,6	101	20	10
10	2	33,4	145,1	152	34	13
10	2	32,3	142,4	120	31	12
10	2	26,8	136,1	100	20	7
10	2	23,8	128,2	100	23	3
10	2	35,2	143,2	140	28	12
10	2	28,4	132,5	90	17	14
10	2	38,5	145	136	27	19
10	2	37,8	147,8	112	28	7
10	2	29,3	137,4	102	29	13
10	2	48	154,2	130	44	11
10	2	38,2	140,5	124	20	4
10	2	37,2	146,2	103	29	6
10	2	31,9	138,2	118	18	4
10	2	32,2	135,2	104	21	7
10	2	29,8	139	110	30	8
10	2	32,2	147,7	124	28	6
10	2	34,2	151,2	140	6	5
10	2	28,7	133,2	125	20	20
10	2	41,1	148,2	106	18	6
10	2	32,5	143,2	107	16	5
10	2	31,2	141,2	98	12	5
10	2	37	145	135	30	25
10	2	30,5	140,2	105	15	9
10	2	42,9	148,2	110	32	3
10	2	35,2	147,5	116	27	5
10	2	33,2	136,1	101	23	7
10	2	37,6	150,2	140	23	10
10	2	35,2	146,1	108	25	10
10	2	33,4	145	144	29	7
10	2	52,1	163,6	145	28	18
10	2	32,4	142,5	156	40	26
10	2	41,8	144,1	54	19	9
10	2	46,8	157,6	121	35	4

10	2	29,4	131,5	105	26	22
10	2	42,7	145	115	29	5
10	2	40,4	147,2	105	22	8
10	2	42,9	145	112	28	9
10	2	35,5	142,3	130	23	7
10	2	28,8	139,3	69	14	9
10	2	38,5	158	115	21	10
10	2	46,8	154,2	125	24	7
10	2	30,1	143,3	100	21	13
10	2	23,5	128	99	1	9
10	2	41,6	145,3	108	31	1
10	2	31,2	137,8	119	20	34
10	2	35,5	134,8	132	22	14
10	2	28,6	132,3	134	35	7
10	2	38,7	144,1	122	27	19
10	2	28,3	139,6	104	26	9
10	2	35,6	148,6	136	23	4
10	2	30,6	144	52	26	20
10	2	35,5	144,3	132	17	15
10	2	25,5	135,4	118	21	25
10	2	30,8	151,2	116	12	10
10	2	39	152,1	136	26	11
10	2	38,2	142,1	108	22	6
10	2	30,7	151,5	138	20	3
10	2	34,8	145	118	18	9
10	2	44,9	147,9	119	18	8
10	2	46,6	151,6	99	30	15
10	2	48,7	158,6	103	12	2
10	2	40,5	150,4	95	17	5
10	2	32,9	142,5	150	32	7
10	2	36,7	147,5	120	29	8
10	2	40,5	142,2	150	33	16
10	2	26,7	142,3	116	15	9
10	2	35	146,3	104	19	6
10	2	39,8	146,3	106	18	4
10	2	29,2	144,2	85	18	2
10	2	37,9	149,8	105	20	4
10	2	35	144,5	140	25	17
10	2	29,8	135	131	24	6
10	2	48,8	140	106	22	7
10	2	82	159,2	85	29	2
10	2	65,1	152	118	6	3
10	2	59,4	142,2	87	18	1
10	2	53,1	144,3	102	29	6
10	2	65,2	155,1	103	23	2
10	2	65,3	148,5	78	18	1
10	2	72,2	157,5	118	24	7
10	2	45,5	140,1	85	23	10
10	2	39	133,9	90	30	5
10	2	55	152	124	37	8

10	2	46,5	149	109	30	9
10	2	54	152,1	90	19	1
10	2	51,4	148,3	105	29	7
10	2	54	158	144	28	10
10	2	53	156,3	146	41	13
10	2	48,8	146,8	122	30	5
10	2	55,1	154,8	133	36	15
10	2	51,2	152	127	35	17
10	2	61,7	159	110	31	5
10	2	35,8	130	81	18	3
10	2	33,1	148	118	28	6
10	2	27,5	133,2	112	28	10
10	2	35,1	134,1	105	29	12
10	2	30	141	126	19	5
10	2	40	151,2	76	18	2
10	2	42	145	135	27	6
10	2	26,5	141,5	138	17	6
10	2	33,3	140,7	106	15	9
10	2	42,8	149	100	18	8
10	2	34,8	143,2	110	20	12
10	2	35,8	143,2	120	30	17
10	2	29,6	139,5	118	8	7
10	2	26,4	128,7	88	27	8
10	2	29,9	141,1	127	19	6
10	2	41,9	145,8	110	18	6
10	2	34,4	136,8	104	25	8
10	2	40,9	155	70	28	7
10	2	31,7	140	125	11	5
10	2	38,2	137,8	114	17	11
10	2	31,8	145,1	107	23	12
10	2	33,3	141,5	90	7	7
10	2	31,4	140,5	120	26	12
10	2	28	137	67	28	10
10	2	28,5	136,7	104	20	7
10	2	44,4	149,2	125	33	1
10	2	40,2	147	124	29	3
10	2	24,9	132,1	126	20	42
10	2	26,7	130,5	70	22	4
10	2	32,1	141,6	125	36	5
10	2	38,2	137,5	129	31	10
10	2	29,6	137,8	115	26	15
10	2	31,6	148	121	21	8
10	2	40,9	155,1	105	28	34
10	2	37,9	149,8	99	27	8
10	2	40,2	157,2	120	27	8
10	2	30,5	138,5	115	27	4
10	2	34,5	147,9	98	8	13
10	2	35,2	142,5	129	29	18
10	2	40,4	148	130	31	11
10	2	39,6	152,7	110	28	6

10	2	29,9	151,8	130	11	8
10	2	27,7	135,2	123	20	10
10	2	45,6	161	95	29	5
10	2	32	140,9	118	21	8
10	2	25,9	135,5	150	42	23
10	2	35,6	146,5	147	27	10
10	2	36,7	141	121	23	8
10	2	44,3	152,4	102	27	17
10	2	31,5	132,2	100	23	6
10	2	39,9	151	100	20	9
10	2	34,1	138,8	118	23	9
10	2	27,4	136,9	126	23	24
10	2	40,4	144,1	104	28	12
10	2	33,8	134,2	104	28	10
10	2	33,3	147,2	120	22	5
10	2	39,4	149,5	154	34	6
10	2	35,1	141,2	134	22	10
10	2	29,8	144,6	118	23	12
10	2	39	147,7	93	26	3
10	2	42	143,5	84	15	3
10	2	64,1	156,1	136	33	4
10	2	70,1	154,8	117	33	20
10	2	59,3	152,5	98	28	7
10	2	56,1	165,2	130	7	12
10	2	42,6	142,5	121	22	6
10	2	48,3	151,7	128	11	8
10	2	50	144,7	83	20	6
10	2	47,1	150	130	26	6
10	2	41	135,4	108	21	10
10	2	49	151,8	155	33	12
10	2	44,3	144,4	89	32	10
10	2	52,8	148,2	123	34	6
10	2	40,3	136,1	95	18	3
10	2	63,5	169,6	105	21	7
10	2	33,6	146,7	102	24	16
10	2	39,8	140,1	119	38	8
10	2	26,4	135,5	115	19	10
10	2	42,5	146,8	90	21	20
10	2	34,5	148,8	98	19	1
10	2	27,8	133,8	120	18	3
10	2	28,2	134	117	28	10
10	2	39	142,8	122	25	6
10	2	43,7	155,6	102	22	4
10	2	41,2	150,1	139	23	24
10	2	32,6	141,1	100	30	22
10	2	30,9	131	115	29	4
10	2	33,6	145,9	122	23	3
10	2	38,6	151,8	109	25	10
10	2	29	134,2	151	32	8
10	2	35,7	154,7	121	27	8

10	2	36,3	144,1	132	24	8
10	2	38,1	151,2	133	27	7
10	2	41,9	150,2	141	29	12
10	2	40,6	140,2	110	23	13
10	2	42,8	146	103	30	3
10	2	30,1	137,6	170	25	1
10	2	33,4	142,1	118	9	9
10	2	31,2	131,4	138	23	10
10	2	36,5	145,2	112	28	5
10	2	29,3	142,1	102	12	5
10	2	26	131,2	110	25	10
10	2	30,8	137,5	170	28	17
10	2	33,4	142	116	25	9
10	2	38,3	143,1	127	27	24
10	2	35	146,5	140	23	3
10	2	27,5	134,5	103	21	10
10	2	32,6	148,1	95	19	5
10	2	29	131	113	27	25
10	2	28,5	134,8	129	23	14
10	2	57,7	146	110	22	7
10	2	113,6	172,1	55	12	7
10	2	39,7	139,2	95	25	6
10	2	45,4	147,3	115	22	5
10	2	48,1	142,1	108	29	5
10	2	50,3	153,2	154	13	3
10	2	54,4	156,2	111	28	10
10	2	40,3	132,6	104	21	12
10	2	43,2	142,4	105	24	10
10	2	56,1	150,2	120	40	10
10	2	45	141,8	96	24	1
10	2	29,6	140,5	145	14	8
10	2	37,2	152,1	92	27	12
10	2	30,3	151,2	100	22	4
10	2	23,8	126,6	125	35	16
10	2	36,4	145,3	110	27	12
10	2	46,6	148,9	90	27	2
11	1	37,7	150,5	101	4	8
11	1	33,1	145,1	150	13	14
11	1	40,4	154,2	140	13	7
11	1	35,6	146,9	121	20	3
11	1	35,2	145,1	166	28	14
11	1	28,8	142,5	151	25	15
11	1	76,8	147	97	16	3
11	1	47,9	149,2	119	20	8
11	1	65,6	159	124	22	2
11	1	43,1	153,9	90	30	2
11	1	33,8	139,5	140	29	12
11	1	42,2	146,2	133	32	3
11	1	34,7	136,1	162	28	9
11	1	32,8	150	138	12	12

11	1	42,4	148,2	155	21	5
11	1	40,7	155,9	150	29	9
11	1	37,5	146,9	137	26	10
11	1	38,2	147,3	122	30	7
11	1	32,9	145	160	23	2
11	1	41,1	156,1	165	30	2
11	1	32,5	146,6	135	23	17
11	1	33,8	140,7	167	33	8
11	1	46,1	158,5	95	24	15
11	1	47,6	160,5	156	29	1
11	1	42,3	147,3	90	23	4
11	1	45,9	157,5	144	19	9
11	1	50,2	168,4	182	21	4
11	1	43	147	176	24	7
11	1	48,3	151	144	22	4
11	1	41,8	157	145	15	13
11	1	41,5	144,7	160	31	30
11	1	34,8	147,5	160	29	17
11	1	71,7	158,6	83	31	2
11	1	50,1	139,9	113	19	3
11	1	63,2	150,8	115	17	5
11	1	55,7	153,1	135	17	10
11	1	55,3	152,3	119	31	2
11	1	54,7	156,6	108	20	6
11	1	51,9	153,1	125	23	12
11	1	30,3	143,2	87	27	1
11	1	41,1	145,5	132	25	18
11	1	35,5	140,8	136	21	15
11	1	35,6	139,5	136	32	11
11	1	44,8	152,2	120	24	8
11	1	34,2	143,6	190	36	23
11	1	37,7	147,3	133	24	3
11	1	32,6	147,5	134	18	6
11	1	36,1	142,2	173	28	12
11	1	28,2	134,3	95	20	8
11	1	35,1	139,8	171	23	23
11	1	32,4	141,1	152	23	25
11	1	33,8	141	158	32	20
11	1	41,7	152,6	141	18	9
11	1	43,3	162,9	180	23	9
11	1	27,8	140,1	128	15	5
11	1	34,4	141	150	23	8
11	1	43,7	151,5	124	19	3
11	1	45,8	153,2	124	25	3
11	1	35,5	142,5	160	20	12
11	1	32	140,9	130	32	15
11	1	30,4	141,8	151	21	5
11	1	40,1	154,1	116	26	6
11	1	30,7	146,9	130	15	12
11	1	30,8	143,5	130	25	8

11	1	27,1	141,6	123	24	3
11	1	42,8	157,8	163	32	17
11	1	38,8	155,5	168	27	32
11	1	56,5	167,1	175	28	27
11	1	34,4	141	168	23	18
11	1	35,7	145,2	131	11	18
11	1	43,6	151,5	165	25	22
11	1	44,8	153,1	125	18	7
11	1	43,1	146,5	146	11	2
11	1	46,7	153,2	101	16	8
11	1	38,1	138,3	157	27	9
11	1	41	152,5	162	31	12
11	1	43,1	145,6	132	28	17
11	1	38,4	147,2	136	25	4
11	1	45,4	152,5	115	16	3
11	1	43,2	163,4	150	27	9
11	1	35,6	145,5	122	12	6
11	1	43,8	151,1	150	21	5
11	1	29,8	142,9	137	31	9
11	1	39,3	151,4	163	36	20
11	1	47,5	159,5	161	21	1
11	1	32,1	146,1	146	21	25
11	1	48,8	166	143	15	1
11	1	44,1	146,1	99	17	3
11	1	41,1	159,2	146	35	5
11	1	32,8	144,2	168	23	11
11	1	29,9	142,5	130	29	6
11	1	30,6	140,3	153	32	10
11	1	30,2	152,8	142	17	22
11	1	38,6	156,5	92	16	13
11	1	60,9	148,3	98	24	1
11	1	57	145,2	109	26	7
11	1	44	144,1	100	16	5
11	1	49,8	144,2	139	22	1
11	1	54	153,5	150	30	4
11	1	50,1	154,2	90	7	4
11	1	54,1	151,2	148	12	7
11	1	59	152,5	133	31	2
11	1	56,3	156	123	19	9
11	1	53,8	151,8	147	31	1
11	1	54,6	150,1	135	26	6
11	1	53,9	147,6	143	28	5
11	1	50,1	146,5	94	7	1
11	1	59,2	161,3	130	20	5
11	1	64,3	160,5	130	28	3
11	1	52,1	155,9	130	31	2
11	1	51,7	151,3	131	30	2
11	1	63,4	158	112	24	6
11	1	52,6	146,2	138	22	2
11	1	61	158,5	93	18	4

11	1	49,1	146,8	127	19	10
11	1	60,3	159,3	142	28	15
11	1	37,2	142,3	160	18	15
11	1	31	145,3	132	20	2
11	1	27,3	145,2	140	18	7
11	1	28,2	143,1	177	21	10
11	1	46,2	149,3	122	22	1
11	1	34,2	143,8	115	25	12
11	1	31,7	137,5	120	32	5
11	1	29,8	136,1	116	23	5
11	1	34,4	140	138	25	7
11	1	30,1	140,5	172	22	7
11	1	38,3	150,1	173	29	8
11	1	38,5	154,2	132	25	8
11	1	24,3	128	160	28	15
11	1	35,3	145,1	140	13	4
11	1	27,4	142,8	135	20	5
11	1	35,1	147	130	22	5
11	1	38,2	149,4	90	27	1
11	1	36,9	147,1	150	24	5
11	1	30,3	145,1	119	25	8
11	1	31,2	142,5	136	27	11
11	1	35,6	142,7	125	28	13
11	1	33,4	144,6	142	28	21
11	1	29,9	142,1	134	25	10
11	1	37,6	142	135	27	7
11	1	48,8	155,5	168	16	15
11	1	34,9	147,5	179	29	9
11	1	32,7	138	139	30	10
11	1	30,9	132,2	172	34	32
11	1	42,5	151,5	143	30	20
11	1	35,9	139,4	170	22	15
11	1	50,8	159,2	152	26	10
11	1	38,1	153,2	122	25	15
11	1	44	153,2	158	31	11
11	1	38,1	154,6	140	28	12
11	1	40,3	152,5	150	28	23
11	1	45,6	154,5	171	20	24
11	1	33,5	142,8	153	27	29
11	1	35,4	148,7	143	23	5
11	1	38,7	151,3	158	17	19
11	1	35,9	145,6	150	22	16
11	1	31,9	138,3	130	30	6
11	1	27,1	140,8	141,2	20	10
11	1	32,2	140,5	160	21	6
11	1	26,2	135	130	22	5
11	1	48,6	161,6	172	28	11
11	1	45,6	148,3	146	32	2
11	1	43,4	157,5	160	17	12
11	1	34,5	142	162,2	22	6

11	1	49,1	160,5	189	31	10
11	1	31,6	139	139	21	10
11	1	30,4	144,2	140	21	10
11	1	39,3	160,1	150	18	5
11	1	35,8	142,6	128	17	19
11	1	34,1	144,3	136	24	18
11	1	41,2	146,4	109	20	1
11	1	36,6	142,4	138	22	28
11	1	34,6	151,5	160	21	9
11	1	40,8	158,3	149	30	9
11	1	68,2	159,2	118	13	1
11	1	68,5	155,3	118	14	3
11	1	77,2	168,3	107	28	1
11	1	80,8	159	100	29	8
11	1	59,1	142	125	27	15
11	1	61,4	160,5	149	30	12
11	1	48,8	148,6	133	19	2
11	1	51,6	150,7	117	18	10
11	1	52,6	146,2	91	17	1
11	1	59,7	152,7	137	26	12
11	1	60,1	158,2	149	21	16
11	1	66,1	167	133	12	4
11	1	55,7	153,3	131	14	10
11	1	48,7	140,3	105	23	12
11	1	57,7	154,4	140	27	15
11	1	38,7	134,3	120	27	10
11	1	53,6	145,2	112	21	2
11	1	56,9	149,2	128	15	8
11	1	58,7	155,8	127	25	1
11	1	26,5	137,1	130	15	12
11	1	29,3	142,3	148	31	12
11	1	37,2	140	142	21	9
11	1	41,2	152,8	157	8	10
11	1	29,7	137	161	34	12
11	1	34,4	145,3	166	30	15
11	1	35,8	143,5	150	27	20
11	1	36,6	150	130	18	2
11	1	35,1	147,1	126	33	7
11	1	33,6	142,8	130	21	5
11	1	42,9	160	140	28	1
11	1	32,3	131	140	30	16
11	1	34,8	146,3	131	19	8
11	1	29,4	135,8	131	24	20
11	1	49,2	155,3	100	19	20
11	1	35,1	148	140	16	20
11	1	46,5	151,8	162	20	20
11	1	33,1	149,2	84	27	6
11	1	31,5	140,7	137	23	8
11	1	26,9	137,1	145	22	8
11	1	28,5	139,8	138	22	6

11	1	43,2	159,2	143	24	20
11	1	32,9	142,7	108	13	3
11	1	38,1	144,3	106	30	5
11	1	36,3	149,5	156	33	13
11	1	42,5	156,6	150	26	25
11	1	47,7	157,3	98	17	4
11	1	32	142,1	148	23	4
11	1	41	155,4	120	28	1
11	1	45,6	150,8	132	22	10
11	1	50,2	160,1	146	26	15
11	1	41,5	143,1	121	20	9
11	1	42,8	159,2	173	35	10
11	1	36,5	145,5	149	23	10
11	1	39	140,2	169	32	15
11	1	68,9	161,3	106	19	1
11	1	58,1	148	130	27	12
11	1	67,7	153,2	118	17	6
11	1	46,7	146,1	147	23	16
11	1	54,6	152,9	125	22	1
11	1	63,7	160,5	119	30	6
11	1	41	132,3	130	31	12
11	1	56,1	149,1	143	23	5
11	1	54,6	156,8	120	25	10
11	1	33,7	144,2	122	22	2
11	1	34,4	139	127	20	9
11	1	41,6	152,8	162	12	15
11	1	45,4	152	157	24	3
11	1	38,3	153	119	13	7
11	1	60,4	147,1	110	28	5
11	1	63	176,2	218	31	20
11	2	36,1	149,2	118	19	8
11	2	35,2	151,1	117	23	5
11	2	50	156	117	27	14
11	2	28,8	141,5	110	19	8
11	2	36,9	154,2	125	15	21
11	2	35,6	148,1	106	28	10
11	2	46,7	162,1	132	21	14
11	2	42,2	146	124	21	7
11	2	42,2	143,2	136	11	4
11	2	35,8	150,5	119	21	5
11	2	35,1	147,8	180	34	4
11	2	43,1	152,8	75	26	6
11	2	30,7	148	114	27	5
11	2	46,2	151,1	110	31	16
11	2	43,6	149,4	140	19	20
11	2	35,2	153,8	134	20	10
11	2	37,7	160,3	121	21	20
11	2	39,4	148,9	138	32	19
11	2	43	158	130	23	15
11	2	34,8	139	141	31	17

11	2	31,6	146,2	117	30	12
11	2	39,9	160	103	19	10
11	2	48,6	152	104	39	2
11	2	38	147,6	120	23	6
11	2	55,3	177,8	81	15	5
11	2	45,2	155	114	17	2
11	2	42,2	154	112	24	8
11	2	44,6	154,9	120	42	31
11	2	31,1	153,8	170	26	16
11	2	41,1	147,7	41	19	9
11	2	45,2	155,3	141	28	10
11	2	35,9	154,2	138	24	10
11	2	36,2	150	110	27	1
11	2	49,8	155	136	28	5
11	2	28,8	140,8	145	23	7
11	2	31,5	144,5	131	19	20
11	2	33,4	145,2	110	29	12
11	2	44,1	163,9	131	18	18
11	2	34,9	145,5	120	23	8
11	2	33,2	154	95	22	18
11	2	40	145,9	119	24	12
11	2	47,6	151,5	140	35	20
11	2	36,1	149,8	136	23	12
11	2	37,6	150,8	145	20	5
11	2	64,8	152,3	108	30	1
11	2	48,7	147,5	105	27	12
11	2	58,1	159,1	136	32	3
11	2	51	150	99	31	2
11	2	65,2	173,2	98	16	5
11	2	63,7	167,1	94	17	11
11	2	63	154	140	33	10
11	2	58,1	150,1	82	12	1
11	2	54	154	142	34	7
11	2	56,2	152,1	96	17	5
11	2	30,2	142,1	129	30	6
11	2	36,7	154	90	31	4
11	2	37,7	148	116	13	2
11	2	35	148,5	120	33	8
11	2	44,2	154	112	18	4
11	2	39,5	147,1	125	23	12
11	2	29,6	139,2	90	8	7
11	2	38,8	154	129	28	7
11	2	31,2	141,5	147	28	9
11	2	37,3	156,2	83	16	3
11	2	36,1	134	106	27	10
11	2	40,2	143,3	70	32	20
11	2	34,7	146	105	30	7
11	2	43,2	145,5	132	25	18
11	2	32,7	143,2	132	32	30
11	2	39,1	150,3	131	22	10

11	2	30,4	146	153	21	20
11	2	27,4	135,8	139	23	20
11	2	43,2	148,8	156	27	32
11	2	38,6	158,5	137	23	16
11	2	38,1	152	129	23	6
11	2	38,8	159	110	24	17
11	2	42,6	151	109	28	4
11	2	43,1	154,3	126	31	8
11	2	44,7	148	130	31	13
11	2	39,4	151,2	151	22	5
11	2	36,9	153,5	85	24	6
11	2	42,1	153,4	114	9	10
11	2	38,4	157	137	27	25
11	2	33,1	142,2	98	23	7
11	2	46	153,5	135	32	27
11	2	37,9	156,3	150	32	20
11	2	35,2	147	123	36	7
11	2	44,7	147	129	31	15
11	2	47,6	159	125	27	13
11	2	30,1	146	100	27	6
11	2	46,2	150,4	94	25	29
11	2	52,7	163,7	127	33	16
11	2	33,2	151,2	122	29	6
11	2	34,5	150	120	24	9
11	2	30,2	142,5	153	23	20
11	2	50,4	155,1	145	28	7
11	2	42,3	156,1	115	34	6
11	2	42,9	152,4	137	14	6
11	2	43,7	152,5	144	23	6
11	2	34,1	148,2	130	30	9
11	2	37,4	141,5	140	21	8
11	2	44,6	163,1	134	24	7
11	2	46,2	151	140	21	7
11	2	45,6	163	138	33	10
11	2	33,6	142,4	158	34	33
11	2	59,9	166,8	98	18	3
11	2	34,3	142,5	123	20	12
11	2	48,2	155	120	40	10
11	2	26,8	139,2	140	26	20
11	2	31,4	143	138	24	13
11	2	47,7	156,9	130	20	5
11	2	45,8	157,5	120	22	20
11	2	46	155,4	118	32	4
11	2	52,8	156,5	160	28	7
11	2	39,1	151	131	22	14
11	2	41,1	159	114	30	2
11	2	36,5	143,9	124	14	15
11	2	44	168,2	125	17	5
11	2	37,7	152	116	25	22
11	2	54,3	166	93	21	3

11	2	52,9	163,4	152	26	7
11	2	44	153,5	77	28	13
11	2	51,9	165,3	112	8	17
11	2	38,1	146	147	42	24
11	2	34,3	149,1	134	15	19
11	2	40	156	110	27	10
11	2	43,1	147,6	117	29	5
11	2	65	150	95	37	11
11	2	65,8	151	109	36	9
11	2	79,1	168,8	56	25	8
11	2	79,6	153,5	110	35	15
11	2	85,2	153,7	70	44	4
11	2	61,9	152,3	100	32	15
11	2	83,3	164	107	24	18
11	2	55,6	149,2	98	13	1
11	2	48,5	142,2	95	33	4
11	2	48,9	147	147	35	8
11	2	54,5	151	100	18	13
11	2	58	153,2	109	19	10
11	2	51,5	145	137	29	11
11	2	52,9	154,1	141	33	3
11	2	56,3	152	133	28	4
11	2	51	151,1	112	11	5
11	2	49,4	148,2	72	10	10
11	2	60,6	153,8	87	17	5
11	2	76,6	169,5	106	11	8
11	2	59,4	160,8	97	31	5
11	2	57,3	155,4	105	22	8
11	2	60,8	156,5	160	32	10
11	2	63,1	154,1	95	12	5
11	2	55,2	146	114	33	5
11	2	50,9	149,1	87	18	1
11	2	56,2	151,8	82	18	6
11	2	55	150,2	104	22	12
11	2	26,6	136	116	30	5
11	2	36,3	149,8	110	32	24
11	2	40,6	146,1	132	16	14
11	2	41,6	156	131	28	6
11	2	38,5	148	130	23	18
11	2	31,7	135,4	121	34	18
11	2	33,8	150,9	70	27	9
11	2	36,3	148,5	121	37	12
11	2	30,2	141,2	165	36	11
11	2	40,3	151,2	125	23	12
11	2	39,9	166	81	13	21
11	2	33,1	134,2	105	30	12
11	2	41,1	155,1	134	26	12
11	2	30,1	136,6	102	34	28
11	2	40,2	142	139	19	26
11	2	31,7	150	120	26	2

11	2	41,7	155,6	136	15	1
11	2	36,2	149,2	118	26	6
11	2	32	146,1	117	26	21
11	2	34,1	143	111	40	5
11	2	43,9	144,5	110	18	6
11	2	39,2	155,8	138	19	3
11	2	39,7	150,2	130	30	23
11	2	49,2	153	149	45	20
11	2	38	147,2	138	28	11
11	2	35	146,1	130	32	7
11	2	41,1	154,5	120	14	1
11	2	32,6	136	160	37	24
11	2	48,7	153,5	152	34	13
11	2	47,1	156,5	127	33	13
11	2	41,2	140,3	109	29	13
11	2	48,2	158	159	40	13
11	2	40,4	147,5	125	23	5
11	2	41,1	157	102	18	11
11	2	34,6	151,3	103	28	15
11	2	40,4	156,8	155	20	20
11	2	41,7	154,2	70	13	5
11	2	38	147,3	112	23	10
11	2	34,9	144,5	111	27	15
11	2	37	141	140	25	10
11	2	32,6	144,5	145	33	16
11	2	36,1	149,5	132	11	8
11	2	41,8	151	118	15	9
11	2	32,5	136,1	126	31	12
11	2	36,4	150,7	110	38	5
11	2	49,1	158,5	135	18	13
11	2	48,7	156	123	33	8
11	2	45,5	160,1	135	36	13
11	2	35,2	150	142	29	22
11	2	44,8	154,5	165	43	20
11	2	41,6	145,6	141	21	21
11	2	30,1	143,1	140	22	14
11	2	57,4	163,5	108	19	5
11	2	42	153,5	143	32	8
11	2	29,8	141,6	150	17	2
11	2	44,5	151,2	125,5	20	3
11	2	43,6	167,2	141	28	17
11	2	44,6	146	107	29	8
11	2	49,8	157,1	102	21	7
11	2	32,7	142,8	126	18	19
11	2	30,2	142,8	101	20	9
11	2	29,1	145,2	126	30	8
11	2	39,4	152	116	23	8
11	2	46	151,5	144	33	16
11	2	42,2	150,2	90	38	15
11	2	31,9	138,2	124	31	7

11	2	34,8	149,2	114	23	10
11	2	39,6	157,8	145	28	11
11	2	42	155,9	141	29	10
11	2	61,1	171,3	126	18	10
11	2	46	153,5	110	23	9
11	2	44,3	144,6	129	18	8
11	2	44,8	157,7	118	30	14
11	2	42,2	156,4	137	30,5	21
11	2	44,2	151	131	32	21
11	2	30,3	146,1	71	12	2
11	2	45,8	155,2	105	22	12
11	2	30	142,2	107	28	13
11	2	44,5	156,4	131	40	17
11	2	43,9	145,2	105	24	6
11	2	44	150,8	90	23	7
11	2	43,7	159,6	100	31	19
11	2	39,4	155,3	135	39	10
11	2	66,9	155,3	128	24	7
11	2	59,7	145,8	90	40	1
11	2	63,2	149,3	59	21	3
11	2	79,2	162,3	95	20	6
11	2	62,6	152	141	28	3
11	2	64,5	151,5	111	24	18
11	2	84,9	163,4	88	39	1
11	2	54,2	149,3	84	21	1
11	2	43,2	142,2	82	18	20
11	2	51,3	151,5	163	46	11
11	2	48,1	149	86	26	10
11	2	53,2	152,5	153	29	16
11	2	45,3	140,3	70	25	7
11	2	48	149	118	31	3
11	2	47,6	148,8	102	20	6
11	2	49,4	142,8	90	27	10
11	2	49,6	150	112	31	20
11	2	52	145	57	18	1
11	2	47,9	145	106	31	1
11	2	45,1	143,2	113	25	6
11	2	50,6	152,3	109	23	16
11	2	60,8	156,4	101	16	9
11	2	52,8	149	130	35	7
11	2	55,9	160,2	105	22	8
11	2	52,2	150,3	131	23	10
11	2	54,2	153,6	125	23	10
11	2	57,7	156,5	102	30	12
11	2	59,2	160,8	98	23	1
11	2	53,2	153,8	101	25	16
11	2	36	148,7	153	30	16
11	2	37,5	153	127	22	11
11	2	48,3	152,1	125	35	3
11	2	32,6	142,1	130	27	15

11	2	35,5	148,6	123	28	22
11	2	32,7	138	134	17	7
11	2	45,4	150,2	125	27	12
11	2	39,2	146,2	105	16	3
11	2	33,8	149,5	150	21	10
11	2	34,8	153,3	122	18	12
11	2	42,8	155,5	82	28	11
11	2	48,8	162,1	113	17	1
11	2	40,2	153,5	112	17	5
11	2	42,7	146	106	23	10
11	2	37,3	145,5	156	41	8
11	2	31,9	140,2	99	28	21
11	2	36,4	156,1	96	17	9
11	2	41	155,5	115	13	8
11	2	43,9	153,3	145	37	9
11	2	39,3	152,3	77	23	1
11	2	47,6	148,3	124	25	11
11	2	49,8	153,6	104	23	7
11	2	32,8	156,3	133	28	12
11	2	44,3	163,7	129	34	9
11	2	41	162	110	34	6
11	2	31,2	147	150	27	6
11	2	40,6	150,3	160	29	18
11	2	37,7	141,6	140	25	30
11	2	45,5	146,8	110	33	19
11	2	43,1	153,8	141	25	4
11	2	36,8	144,2	127	23	34
11	2	49,5	154	155	30	10
11	2	52,2	164,3	144	29	10
11	2	48,8	150,3	92	22	3
11	2	40,3	154,5	140	29	13
11	2	36,2	145	127	26	25
11	2	42,1	152,8	129	28	20
11	2	31,4	145,7	75	13	1
11	2	35,5	150,4	130	26	1
11	2	31,9	138,3	108	19	7
11	2	39,3	155,2	89	15	17
11	2	83,6	157,2	110	33	8
11	2	61,6	164,8	106	17	16
11	2	54,2	153,8	109	23	12
11	2	59,8	154	92	14	3
11	2	50,9	151,9	105	10	8
11	2	45,5	144,9	107	32	11
11	2	59,8	158,5	128	35	4
11	2	53,5	151,6	84	14	1
11	2	44,3	142,2	90	28	14
11	2	56,1	154,2	108	29	8
11	2	50,4	143,5	109	17	4
11	2	55,8	152	120	26	10
11	2	37,5	144,1	123	17	9

11	2	34,3	136,5	129	26	1
11	2	37,7	149	153	24	15
11	2	42,7	154,2	105	24	20
12	1	42,2	152	178	27	10
12	1	36,2	158,4	146	16	10
12	1	52,8	168	171	29	30
12	1	40,2	154,9	148	21	16
12	1	41,6	146	105	18	2
12	1	50,4	160,6	194	28	30
12	1	50,5	163,2	170	26	8
12	1	44,2	159,9	141	6	15
12	1	70,2	157,3	143	18	12
12	1	72,6	155	138	20	12
12	1	57,2	157,5	148	25	1
12	1	52,3	151,7	146	28	12
12	1	57,1	160,7	123	22	6
12	1	34,1	148,5	114	28	19
12	1	41,4	156	120	15	7
12	1	35,6	148	155	24	7
12	1	50,4	161,2	130	21	8
12	1	54,8	166,3	175,1	28	21
12	1	49,5	161,2	141	14	30
12	1	40,3	149,8	162	21	5
12	1	41,9	150,5	130	23	10
12	1	47,9	163	165	20	10
12	1	37	147,3	125	11	10
12	1	48,6	153,2	178	19	17
12	1	34,3	136,2	135	20	7
12	1	25,3	131,5	142	25	10
12	1	53,1	158	135	6	3
12	1	49,4	160	141	31	6
12	1	58,9	168,5	134	36	7
12	1	33,4	147,6	141	28	12
12	1	49,2	151	140	26	4
12	1	36,8	140,2	136	21	12
12	1	48,7	150,7	153	20	4
12	1	48,5	156,5	135	12	8
12	1	54,9	165,5	141	24	10
12	1	39,7	140,8	156	20	21
12	1	40,7	154,2	169	20	6
12	1	41,2	146	163	21	21
12	1	58,1	165,2	160	23	21
12	1	44,3	150,9	128	14	13
12	1	47,7	151	153	32	10
12	1	49,8	158,2	143	21	18
12	1	38,3	148,1	137	33	13
12	1	65,6	151,7	122	23	10
12	1	65,8	161,1	103	23	10
12	1	55,4	152,5	127	5	2
12	1	61	154,5	127	8	8

12	1	51,8	150,2	121	23	7
12	1	54	152,8	132	28	8
12	1	61,3	162,3	129	9	1
12	1	58,4	162,3	177	27	7
12	1	53,9	148,8	147	28	8
12	1	62	159,5	156	25	10
12	1	34,7	147,7	150	20	14
12	1	42,3	157,9	153	31	10
12	1	40,4	157	148	28	20
12	1	42,2	151,5	115	34	10
12	1	40,2	156,4	170	29	6
12	1	43,9	151,1	154	38	24
12	1	39,5	150,2	174	31	8
12	1	33	141	137	22	8
12	1	38,2	154,5	160	23	6
12	1	34,4	148	181	25	20
12	1	50,1	168,7	173	20	6
12	1	46,2	158,3	125	23	8
12	1	31,4	144	182	29	17
12	1	42	160	150	26	12
12	1	46,1	161	133	16	1
12	1	45,5	165,2	205	30	32
12	1	51,3	158,6	108	15	3
12	1	31,9	141	128	29	15
12	1	43,8	167,3	140	23	2
12	1	41,6	153,1	164	13	5
12	1	42,6	153,2	156	20	3
12	1	31	144,8	159	24	13
12	1	46,8	162,4	134	21	1
12	1	43,2	150,3	140	20	32
12	1	37,9	146	139	18	18
12	1	44,6	149,2	130	9	17
12	1	33,9	143,2	152	16	10
12	1	50,5	159,2	159	23	19
12	1	39,5	150,5	163	11	10
12	1	40,2	156,1	143	23	11
12	1	42,8	151,5	169	24	15
12	1	45,4	156,7	136	7	3
12	1	45,3	160,7	150	17	10
12	1	28,6	135	121	28	11
12	1	30,7	136,5	145	24	6
12	1	35	142	161	20	17
12	1	41,5	150,7	167	25	25
12	1	32,5	148,6	161	30	20
12	1	51,6	161	152	23	13
12	1	45,1	152	97	15	3
12	1	38,5	140,2	132	28	20
12	1	30	135,2	150	27	4
12	1	40,8	152	163	20	15
12	1	42,2	163,8	165	16	19

12	1	38,9	154,9	160	27	9
12	1	52,4	164	159	22	7
12	1	50,1	158,2	160	25	14
12	1	42,6	153	166	17	8
12	1	44,4	156,8	152	28	19
12	1	58,1	169,2	139	23	18
12	1	44,8	149,1	145	20	18
12	1	47,4	164,6	161	28	12
12	1	42,4	158	164	28	11
12	1	50,7	160,3	146	24	2
12	1	42,2	147	160	24	14
12	1	39,3	148,7	140	18	12
12	1	44,6	161,4	174	22	8
12	1	41,6	162,2	160	25	16
12	1	32,7	152,6	156	11	9
12	1	55,2	160,2	148	10	23
12	1	48,2	161,9	148	13	1
12	1	40,5	147	151	36	9
12	1	42	154,2	167	29	10
12	1	61,7	168,1	140	6	2
12	1	60,3	171,5	191	33	15
12	1	39,8	150,3	175	23	9
12	1	56	170	140	20	9
12	1	42,4	154,3	139	12	6
12	1	37,3	145,8	174	15	12
12	1	51,6	162,5	170	22	21
12	1	35,3	155,2	138	23	12
12	1	67,9	176,1	171	18	21
12	1	41,8	158,2	141	34	23
12	1	40,1	148,1	149	25	17
12	1	44,5	147	172	23	2
12	1	40,3	153	156	27	9
12	1	39	156	120	6	12
12	1	32,5	155	164	9	8
12	1	45,6	159,2	193	39	5
12	1	49,2	158,4	210	24	16
12	1	31,5	149	157	23	11
12	1	47,5	158	161	28	1
12	1	57,9	147,3	117	23	7
12	1	90,3	179,9	112	19	1
12	1	78,4	171	132	13	2
12	1	54	155	130	21	2
12	1	63,1	156,8	114	24	1
12	1	61,8	164	125	12	10
12	1	56,9	150,7	132	16	11
12	1	67,4	161,2	107	17	1
12	1	48,3	146,5	130	28	1
12	1	57,1	153	150	30	8
12	1	51,5	154	105	13	2
12	1	60,6	152,8	115	21	7

12	1	57,7	152,3	119	30	1
12	1	60,2	160	121	20	5
12	1	41,3	138,4	140	19	9
12	1	55,6	144,2	139	20	2
12	1	53,8	149,5	106	30	2
12	1	65,8	165,6	127	32	1
12	1	63,5	163	175	27	9
12	1	52,3	152	136	24	1
12	1	60,7	160	150	31	15
12	1	55,8	157,1	125	23	18
12	1	55,2	154,5	139	23	15
12	1	47	147,1	150	20	8
12	1	59,3	163,2	156	35	11
12	1	42,8	154,2	171	14	9
12	1	38,4	147,8	154	14	24
12	1	40,8	144,5	163	30	4
12	1	30,2	147,5	178	29	15
12	1	44,7	161,5	140	25	15
12	1	39,5	141,8	127	11	8
12	1	45,8	146,5	125	17	3
12	1	32,3	139,9	162	25	20
12	1	30,7	140,3	147	30	22
12	1	40,2	158,1	129	11	7
12	1	34,1	160,2	150	16	15
12	1	42,8	160,5	161	13	3
12	1	31,2	148	159	10	5
12	1	52,6	162,8	142	35	1
12	1	38,7	157,3	145	28	15
12	1	38,4	146,1	118	19	16
12	1	46,8	154,6	119	21	7
12	1	28,4	137,5	132	24	12
12	1	37,1	139,6	150	31	10
12	1	38,6	144	135	15	16
12	1	40,5	154,7	152	30	9
12	1	49,5	160,5	98	18	3
12	1	44,6	155,2	146	21	12
12	1	48	155,9	107	20	1
12	1	47,4	155,2	158	32	11
12	1	45,7	150,7	146	14	4
12	1	41,3	155,4	157	23	10
12	1	48,9	160,8	148	22	15
12	1	41,5	149	135	13	13
12	1	46,3	159,2	165	17	14
12	1	33,6	147,7	132	17	1
12	1	40,6	146,2	140	22	4
12	1	32,5	148,2	166	28	14
12	1	55,3	167,3	142	7	14
12	1	57,6	177,3	159	34	18
12	1	44	147,8	152	21	18
12	1	58,6	164,7	119	10	3

12	1	31,6	148,3	134	25	8
12	1	48,3	153,6	168	27	2
12	1	48,5	158	133	16	10
12	1	45,3	144,5	144	21	8
12	1	45,1	154,9	139	28	10
12	1	30,6	143,2	136	25	8
12	1	48	158,6	176	23	11
12	1	43,5	158,7	167	35	23
12	1	40,2	154	165	21	12
12	1	35,1	149,9	130	13	3
12	1	46,1	153	172	24	15
12	1	30,8	155,8	184	25	9
12	1	44,1	158,9	143	26	5
12	1	42,1	153,2	169	32	17
12	1	43,2	157	148	25	15
12	1	48,4	163	110	27	7
12	1	34,7	142,4	147	32	14
12	1	40,4	158	150	27	16
12	1	34,3	149,5	154	26	14
12	1	53,1	165,3	96	21	9
12	1	40,2	166,5	139	30	1
12	1	54,7	161,5	158	35	10
12	1	34,8	151,8	136	22	3
12	1	73,2	162,5	150	30	3
12	1	81,2	165,3	76	38	1
12	1	69,5	154,3	112	28	5
12	1	74,5	161,5	147	33	8
12	1	77,2	158,2	112	24	3
12	1	84,4	164,6	103	20	2
12	1	91,6	180,1	93	32	1
12	1	51,8	147,1	115	25	5
12	1	63,1	162,9	198	33	1
12	1	49,7	151,5	103	26	3
12	1	54,6	145	92	27	3
12	1	48,4	140	148	20	14
12	1	48,3	144,6	136	22	8
12	1	54,3	152,3	170	31	1
12	1	64	161,3	138	22	11
12	1	52,1	152,9	145	26	13
12	1	81,5	178,3	145	23	28
12	1	54,2	155,3	129	20	3
12	1	47,8	139	120	10	13
12	1	58,3	153,5	107	19	4
12	1	74	178,1	144	2	4
12	1	60,5	161,8	150	15	5
12	1	67,2	175	153	26	12
12	1	57,3	159,2	128	31	1
12	1	51	146,3	103	25	1
12	1	53,3	147,7	127	8	2
12	1	41,1	148,5	145	32	16

12	1	33,7	135,6	128	26	12
12	1	43,2	146,1	88	28	7
12	1	28	154,2	141	19	38
12	1	31,5	142	157	21	1
12	1	31,2	150,2	127	22	8
12	1	33,3	141,2	144	25	12
12	1	41,2	151,2	148	20	12
12	1	28,3	138	124	24	21
12	1	35,4	145,7	184	16	14
12	1	57,2	172,8	143	29	7
12	1	37,5	145,6	154	14	13
12	1	43,2	146,3	163	21	21
12	1	34,1	144	127	17	14
12	1	42	148,1	93	25	3
12	1	48,1	156	157	14	5
12	1	39,6	145,9	128	27	8
12	1	38,3	146,3	138	22	19
12	1	50,3	158	113	19	9
12	1	46,2	158,6	130	14	3
12	1	51,4	170,3	157	25	5
12	1	33,1	145,5	112	24	4
12	1	33,3	143	135,9	29	19
12	1	40,2	148,5	145	26	32
12	1	42,7	146,8	156	25	15
12	1	36,4	154,2	135	21	10
12	1	37,3	151,5	142	10	1
12	1	45,4	159	184	29	19
12	1	50,2	163,1	162	28	8
12	1	49	158	119,1	5	1
12	1	47,6	161,2	153	25	13
12	1	41,2	157	170	30	8
12	1	51,5	167,2	148	20	7
12	1	45,2	155,2	180	25	21
12	1	41,3	154,6	155	18	6
12	1	61,4	151,9	120	20	1
12	1	74,8	163,2	166	25	4
12	1	56,8	160,1	143	31	10
12	1	61,5	166,5	158	23	10
12	1	61,2	153,9	124	30	3
12	1	63,3	159,1	107	21	3
12	1	72,3	175	192	28	22
12	1	56,3	156,5	128	20	1
12	1	31,2	135,5	153	23	16
12	1	47,2	154,9	120	11	6
12	1	38,3	144,9	150,3	26	21
12	1	33,4	142,1	125	23	5
12	1	33,3	138,1	161	26	10
12	1	56,2	164,5	151,5	33	20
12	1	62,8	150,2	148	26	8
12	1	70,8	163,9	108	25	2

12	2	44,5	157	95	31	20
12	2	44,3	163,2	93	28	15
12	2	40,6	153	122	33	24
12	2	71,4	162	127	38	2
12	2	32,9	143	110	31	9
12	2	57,7	169,8	148	22	18
12	2	50,7	170,8	140	25	6
12	2	43,5	157,4	107	29	10
12	2	38,3	151,1	118	21	7
12	2	38,8	157	154	25	7
12	2	34,4	140	114	23	10
12	2	47,5	155,7	129	28	20
12	2	36,1	145,8	134	28	31
12	2	44,2	156	154	32	4
12	2	36,9	151,5	129	33	14
12	2	49,5	154	117	27	22
12	2	53,9	164	137	28	13
12	2	51,3	161,4	110	35	10
12	2	47	150,2	103	35	5
12	2	35,6	154,8	104	17	21
12	2	41,3	147,5	123	27	16
12	2	44,5	158,5	123	25	12
12	2	47,1	159,2	123	28	12
12	2	52,5	167	127	29	10
12	2	33,8	148	128	26	5
12	2	43,8	158	123	21	16
12	2	52,1	160,1	125	29	12
12	2	35,7	149,5	143	24	9
12	2	35,2	156,9	102	29	20
12	2	52	162,8	125	32	12
12	2	49,8	164	102	25	2
12	2	31,2	145,6	112	16	11
12	2	41,1	159,9	140	31	8
12	2	46,7	164,5	119	21	8
12	2	45,8	158	140	34	6
12	2	59,9	163,2	121	20	12
12	2	80	154	50	23	4
12	2	59,8	159,5	120	16	5
12	2	59,8	152,6	110	30	4
12	2	64,2	163,8	87	17	1
12	2	59,6	154,5	117	27	7
12	2	56	150,8	115	27	27
12	2	61,2	157,2	99	35	7
12	2	63,4	164	125	37	11
12	2	49,8	161,9	120	34	18
12	2	35,7	150,3	132	26	11
12	2	44,7	142,1	126	25	5
12	2	43	156	100	25	20
12	2	52,8	158,3	143	32	14
12	2	47,1	163	131	32	12

12	2	45,1	156,5	141	36	11
12	2	42,1	155,6	125	9	11
12	2	53,6	158	90	23	8
12	2	40,5	157	123	33	5
12	2	39,3	152,6	116	23	20
12	2	49,2	157	125	18	10
12	2	36,5	141,7	127	18	9
12	2	42,3	151,3	153	28	3
12	2	39,8	150,2	101	37	8
12	2	33,7	148,9	130	24	22
12	2	42,2	155	155	27	4
12	2	53,1	159	110	25	9
12	2	52,1	160,1	105	25	12
12	2	46,9	162,1	104	30	1
12	2	51,3	161	170	35	8
12	2	35,2	149,5	130	32	22
12	2	42,5	155,5	163	30	22
12	2	31,6	142	102	15	7
12	2	48,2	166,3	111	22	7
12	2	45,8	153	116	11	7
12	2	45,1	157,3	125	21	4
12	2	44,1	159,4	103	33	3
12	2	43,2	162,5	134	30	14
12	2	45,2	151,5	77	16	9
12	2	49,5	155,8	127	21	31
12	2	44,2	151,2	85	14	3
12	2	39,4	151	156	18	12
12	2	34,1	140,2	129	21	5
12	2	38,1	146,2	152	27	12
12	2	46,1	154,5	142	30	13
12	2	45,2	156,1	129	21	8
12	2	49,2	156	106	31	2
12	2	47,3	151,3	130	32	11
12	2	35,8	153	161	23	30
12	2	57,1	164,2	75	21	2
12	2	50,7	152	113	21	16
12	2	30,6	147,3	131	28	16
12	2	42,7	150,3	103	31	8
12	2	40	158	132	21	12
12	2	37,3	152,1	102	27	1
12	2	61,5	166,1	115	22	1
12	2	47,3	158,1	157	21	15
12	2	42,6	152,1	109	26	11
12	2	39,8	158,6	119	28	22
12	2	52,3	166,7	139	34	13
12	2	45,3	150,1	153	35	12
12	2	50,4	161,5	110	15	1
12	2	35,2	148,5	120	30	25
12	2	40,5	160,2	85	19	11
12	2	44,3	142	119	36	19

12	2	49,9	156,4	127	26	8
12	2	45,3	166	149	29	13
12	2	42,1	150	122	27	16
12	2	40,8	142,1	120	29	2
12	2	42,3	160,8	110	14	1
12	2	38,9	152,3	121	26	9
12	2	50	158,5	186	31	3
12	2	43,2	156,2	165	40	18
12	2	39,2	163,1	119	23	4
12	2	48	163,5	144	23	25
12	2	32,3	145	104	31	1
12	2	40,7	150,3	105	27	10
12	2	53	156	108	23	11
12	2	40	152	144	32	8
12	2	41	167,2	119	18	7
12	2	46,3	160	138	24	13
12	2	46,2	165	128	14	2
12	2	39,8	153,5	132	17	23
12	2	58,8	167,3	122	34	3
12	2	38,8	154	136	20	7
12	2	43,3	153	167	23	2
12	2	37,2	152,7	123	31	8
12	2	50,8	162,8	126	27	2
12	2	46,6	155,1	93	13	10
12	2	78	157	89	40	4
12	2	72,4	158,1	99	18	3
12	2	51,2	150	113	5	12
12	2	66,5	160	103	25	5
12	2	63,3	155,3	107	18	6
12	2	66,5	162,1	90	29	2
12	2	57,3	151,5	121	50	8
12	2	56,1	154,8	130	22	12
12	2	56,1	157	108	26	11
12	2	63,9	161,2	136	23	5
12	2	53,9	152,3	120	18	17
12	2	55,5	155	160	31	8
12	2	66,6	164,1	136	40	33
12	2	60,2	157,5	100	20	6
12	2	60,4	156,1	112	22	6
12	2	56,1	149,5	103	32	17
12	2	62,1	159	119	27	11
12	2	69,8	159,2	119	22	1
12	2	62,8	167,5	125	21	6
12	2	69,9	165,1	95	16	7
12	2	38,6	150,9	141	32	16
12	2	25,9	141,5	127	16	23
12	2	52,3	160,8	126	21	5
12	2	50,2	164	119	31	6
12	2	39,2	157,7	125	21	8
12	2	57,2	170,1	85	20	9

12	2	41,7	152,4	131	16	10
12	2	53,7	165,1	96	17	1
12	2	32,7	151	139	29	5
12	2	37,7	142,6	135	36	13
12	2	40,8	162,3	130	27	4
12	2	49,2	156,9	130	41	10
12	2	30,7	145,9	140	20	15
12	2	36,1	141,6	143	33	10
12	2	34,9	140,5	111	22	15
12	2	30,2	149,1	94	31	1
12	2	42,4	156,2	119	8	6
12	2	46,2	165,5	102	19	8
12	2	38,5	151,5	123	21	5
12	2	46,5	163,2	105	23	3
12	2	44,3	147,9	105	15	12
12	2	48,3	156,7	115	25	2
12	2	48,2	152,8	97	14	11
12	2	38,5	149	140	19	7
12	2	60,3	170,5	138	34	7
12	2	31,2	144,5	130	27	10
12	2	37,9	151	156	28	12
12	2	38,9	146,5	150	25	2
12	2	43,4	140,5	58	19	1
12	2	47	154	105	31	18
12	2	53	154,3	108	22	10
12	2	39,1	150,3	130	16	15
12	2	32,8	140	151	31	16
12	2	39,2	150,3	107	19	15
12	2	51,6	164,6	105	25	5
12	2	43,6	156,3	142	37	15
12	2	40,4	149,4	127	21	1
12	2	51,7	159,2	110	26	3
12	2	41,3	159,3	135	23	9
12	2	42,6	148,8	108	24	2
12	2	37,9	147,9	130	18	5
12	2	38,2	156,8	138	22	15
12	2	48,9	157,4	120	26	3
12	2	53,3	166,6	134	30	11
12	2	44,4	152,1	153	33	5
12	2	44,7	150,9	101	22	8
12	2	51	161,2	132	31	18
12	2	38,5	160	100	35	23
12	2	45,6	158,2	103	23	2
12	2	43,3	164,6	135	19	12
12	2	40,1	157,6	120	28	15
12	2	39,5	152,2	120	35	25
12	2	48,2	162,3	116	30	10
12	2	39,3	160	140	28	13
12	2	55,6	160,2	135	32	10
12	2	38,9	152,1	132	29	8

12	2	46,4	160,1	97	19	8
12	2	41,5	150	130	18	10
12	2	53,2	155,5	63	23	5
12	2	48,5	158,5	77	21	9
12	2	42,1	158,6	138	26	15
12	2	35,7	148,8	89	26	2
12	2	32,9	143,4	132	35	20
12	2	62,5	166,5	146	27	10
12	2	48	153,2	112	25	8
12	2	53,5	155,2	142	34	31
12	2	32,9	149,3	140	11	7
12	2	43,1	154,3	122	18	7
12	2	35,9	151	123	34	8
12	2	47,1	148,7	130	26	7
12	2	49,9	165	135	27	12
12	2	33,4	146,7	90	21	7
12	2	44	159	136	35	10
12	2	42,3	155,1	120	30	1
12	2	40,6	148,9	104	35	3
12	2	35,3	158,3	128	30	16
12	2	44,9	154,5	100	25	5
12	2	35,5	145,4	170	25	2
12	2	43,2	157	126	31	23
12	2	52,6	162,5	94,5	25	8
12	2	40,2	152	170	33	13
12	2	42,6	156	115,7	30	3
12	2	42,5	156	120	27	5
12	2	31,7	152,1	132	23	10
12	2	45,7	173	122	13	4
12	2	76,4	163	121	29	4
12	2	78,9	157,5	104	13	5
12	2	76,2	166	82	6	10
12	2	65,1	149,5	108	21	6
12	2	57,5	149,5	96	31	10
12	2	58,3	160,2	105	29	6
12	2	52,5	153,6	110	30	15
12	2	57,8	153	144	26	25
12	2	63,6	159,5	142	41	30
12	2	54,5	151,3	129	35	12
12	2	55,5	149,5	140	27	10
12	2	63,8	158,3	105	18	14
12	2	60,3	158	123	35	7
12	2	57,2	155,6	116	26	7
12	2	56,1	155	144	36	30
12	2	54,2	149	100,8	29	17
12	2	58,9	155,3	106	21	4
12	2	38	151,9	117	23	20
12	2	53,6	161	110	46	2
12	2	40,5	154,5	167	36	17
12	2	43,2	143,6	129	32	5

12	2	40,6	152,3	120	26	6
12	2	46,7	155,2	127	29	30
12	2	43	152,2	161	30	7
12	2	39,4	151,6	123	25	5
12	2	55,4	159,5	123	35	7
12	2	36,2	143	125	32	5
12	2	35,1	150,8	139	37	10
12	2	49,3	154,6	107	27	10
12	2	38,5	136,1	131	21	3
12	2	31,8	142,1	103	30	10
12	2	45	152,3	105	16	4
12	2	36	146	88	22	7
12	2	25,6	146,3	130	9	4
12	2	46,5	158	130	31	7
12	2	38,5	150	128	20	10
12	2	36,8	143	172	27	30
12	2	32,1	146,8	107	9	2
12	2	46,3	148,5	130	26	20
12	2	45,2	164,5	138	21	12
12	2	36,4	149,4	121	28	5
12	2	44	155,5	110	29	3
12	2	43,4	165,1	120	27	4
12	2	59,7	169,8	107	9	2
12	2	42,9	148,3	130	30	23
12	2	47,7	158,9	120	37	10
12	2	46,9	156,3	110	20	4
12	2	44,3	163,2	121	19	5
12	2	47,6	163	93,3	34	6
12	2	40,1	145,6	120	26	34
12	2	44,9	157,9	123	23	12
12	2	42,8	154	97	17	6
12	2	50,5	162,7	131	23	11
12	2	49,8	159,8	89	31	16
12	2	57,2	165,2	137	24	2
12	2	46,4	155,2	81	21	5
12	2	47,1	163,2	123	25	8
12	2	49,7	156,3	123	31	1
12	2	39,8	146,3	107	31	7
12	2	41,4	156,8	115	16	2
12	2	42,5	157,8	122	23,9	3
12	2	29,7	140,4	110	23	1
12	2	43	155,4	115	27	2
12	2	46	163,2	141	23	3
12	2	50	153,8	138	23	7
12	2	51,4	157	118	16	8
12	2	86,1	163,7	75	21	1
12	2	63,3	157,1	115	26	1
12	2	58,5	147,4	166	36	4
12	2	60,6	159	146	23	8
12	2	63,8	158	158	39	28

12	2	68,4	157,8	130	29	22
12	2	67,2	159,2	113	22	6
12	2	40,3	152,8	110	28	27
12	2	36,3	153,9	118	26	18
12	2	35,1	155	177	31	15
12	2	51,5	152,9	130	19	12
12	2	51,2	154	128	34	15
12	2	39,1	144,7	148	19	8
12	2	55,9	149,3	103	31	2
12	2	53,7	151,4	142	24	18
12	2	54,8	147,3	99	25	4
13	1	58,3	164	167	36	20
13	1	53,5	161,8	179	32	15
13	1	48	166,9	141	32	22
13	1	53,5	157,8	130	13	7
13	1	50,1	172,6	181	17	13
13	1	43,8	158,2	151	22	19
13	1	80,7	189,2	179	26	18
13	1	55,7	176,7	143,5	17	6
13	1	58	176	211	27	21
13	1	53,8	161	150	14	3
13	1	55,2	165,4	158	23	4
13	1	69,4	157,3	145	18	6
13	1	95,3	167,6	100	25	3
13	1	73	164,9	148	21	12
13	1	44,2	157,9	173	10	20
13	1	47,9	160	133	20	5
13	1	40,5	168,5	160	7	7
13	1	47,2	160	165	27	18
13	1	46,9	161,8	150	21	20
13	1	51,6	161,2	120	18	30
13	1	50,9	156,3	174	19	22
13	1	41,8	157,8	200	23	15
13	1	43,2	151,5	145	28	18
13	1	66,1	178,2	175	28	17
13	1	51	164,9	184	25	14
13	1	43,8	171,8	135	18	21
13	1	62,8	169,8	153	25	12
13	1	55,6	158,3	142	10	3
13	1	58,6	165	153	22	18
13	1	48	164	183	32	5
13	1	55,1	171,5	179	33	10
13	1	58,4	176,2	163	16	16
13	1	56,4	165	145	23	12
13	1	49,2	166,2	186	22	18
13	1	56,4	171	172	27	8
13	1	70,9	180,3	140	10	22
13	1	50	163	170	26	10
13	1	75,5	183,9	195	25	13
13	1	53,4	170,2	143	31	23

13	1	49,1	171	180	34	19
13	1	56,8	170,5	168	36	30
13	1	45,9	166	181	21	6
13	1	76,3	158,8	148	33	14
13	1	77,8	163,9	127	10	3
13	1	86,2	173,5	130	38	5
13	1	65,1	155,5	159	32	24
13	1	64,2	166,5	145	30	8
13	1	71	164,2	114	15	2
13	1	54,8	156,8	100	26	11
13	1	57,3	158,8	120	16	9
13	1	59,2	156,8	146	19	2
13	1	53,4	160,1	130	28	10
13	1	46,2	152,3	147	31	7
13	1	38,4	157	169	22	9
13	1	58,3	165,3	123	19	7
13	1	35,4	152,3	180	31	14
13	1	46,5	161,5	145	22	10
13	1	41,1	164,5	158	23	18
13	1	34,1	147,8	160	25	12
13	1	48	163,1	124	17	1
13	1	51,5	156,3	115	26	16
13	1	53,6	168,6	174	24	10
13	1	49	153,1	142	20	19
13	1	60,2	164,6	177	10	10
13	1	28,9	144,4	145	28	19
13	1	60,8	167,4	119	25	13
13	1	43,7	151,4	139	22	8
13	1	42,9	166	153	25	18
13	1	55,9	174,3	140	7	3
13	1	55,3	168,7	199	30	15
13	1	54	167,1	159	30	5
13	1	54	167,1	159	30	5
13	1	37,4	156,1	157	15	12
13	1	43,2	148,6	166	25	12
13	1	46,4	151,2	155	25	4
13	1	60,3	172,7	210	33	31
13	1	64,2	178,3	180	32	5
13	1	57,2	164,1	160	12	2
13	1	60,3	176,3	160	23	6
13	1	41,5	154,2	139	31	6
13	1	51,5	172,2	160	19	13
13	1	53,3	156,7	128	27	4
13	1	37,1	150,2	122	20	16
13	1	46,9	152,9	179	34	10
13	1	36,5	149,4	150	22	12
13	1	34,1	148,5	177	30	7
13	1	49,2	163,5	160	7	11
13	1	58,6	176,7	158	25	14
13	1	57,2	164,5	150	18	9

13	1	59	172,3	149	18	8
13	1	43,3	161	168	23	14
13	1	48,1	168,5	199	30	17
13	1	52,6	163,8	142	25	2
13	1	39,4	154,3	176	18	25
13	1	48,2	146,7	131	22	19
13	1	47,9	158	140	20	15
13	1	53,2	167	201	23	14
13	1	59,5	178,5	177	26	20
13	1	50,1	165,1	157	26	14
13	1	37	165,2	122	21	6
13	1	42,5	162,1	185	28	20
13	1	48,2	175,1	142	13	4
13	1	43,2	161,5	159,7	30	5
13	1	52,2	163,5	190	24	7
13	1	45,1	161,2	183	18	9
13	1	48,8	163,5	187	37	30
13	1	46,5	163,8	110	12	20
13	1	45,6	153,5	158	34	30
13	1	45,7	159	151	13	10
13	1	43,1	156,9	181	28	18
13	1	55,4	170	149	31	1
13	1	36,7	154	147	31	10
13	1	62,7	175,6	160	28	20
13	1	52,8	164,6	193	40	12
13	1	52,2	170,3	173	32	9
13	1	40	151,8	153	33	17
13	1	40	162	102	26	15
13	1	54,4	168,1	120	16	2
13	1	62,6	171,3	178	17	5
13	1	50,2	170,6	167	19	16
13	1	58	166,5	200	38	24
13	1	40,3	152,1	160	15	12
13	1	54,1	164	149	18	6
13	1	39,1	149,1	151	25	10
13	1	56,8	175,2	158	26	1
13	1	43,8	170,1	147	11	6
13	1	55,1	158,7	163	38	3
13	1	62,8	185,1	194	27	17
13	1	46,9	153,6	170	24	14
13	1	42,2	168,2	198	38	9
13	1	60,4	163,8	157	27	9
13	1	47,3	161,2	200	25	11
13	1	54,6	163,5	159	18	18
13	1	38,3	154,2	188	23	20
13	1	45,1	151	128	16	5
13	1	53,4	166,6	150	29	15
13	1	50,9	163,5	180	22	12
13	1	52,6	166,5	159	18	10
13	1	58,5	168,7	208	39	25

13	1	48,2	163,5	148	9	11
13	1	65,4	171,5	172	33	20
13	1	42,3	165,8	180	14	25
13	1	39,4	155	123	28	5
13	1	61,4	168,9	151	34	30
13	1	58,6	165,2	186	30	19
13	1	44,2	163,8	186	18	15
13	1	56,8	163	178	28	10
13	1	46,2	155,2	134	16	9
13	1	60,2	177	182	27	15
13	1	46,6	156,8	163	23	16
13	1	99,8	170,3	105	4	6
13	1	73,4	158	89	14	3
13	1	97	172,8	139	24	10
13	1	81,5	166,8	115	13	5
13	1	96,8	180,2	135	17	12
13	1	74,2	167,5	130	24	1
13	1	69,2	162,8	121	23	8
13	1	64,5	168	190	26	13
13	1	53	153,2	150	22	12
13	1	71,2	173,5	151	26	12
13	1	72,2	177	206	1	11
13	1	65,2	158,2	140	29	1
13	1	60,2	161,5	198	34	10
13	1	72,1	169,5	184	29	5
13	1	69,3	165,8	125	31	12
13	1	65,8	171,5	210	40	30
13	1	57,3	157,5	169	25	15
13	1	70	166,4	117	17	1
13	1	70	172,1	177	26	19
13	1	68,5	158,9	132	13	1
13	1	69,1	160,2	118	14	4
13	1	69,1	169,4	126	18	20
13	1	65,9	167,8	130	27	2
13	1	73,4	168,1	132	9	6
13	1	72,8	167	135	29	1
13	1	70,7	174,5	135	30	10
13	1	74,3	165,2	140	20	5
13	1	73,2	165,8	145	23	1
13	1	65,1	169,6	185	23	25
13	1	57,8	168,4	184	23	17
13	1	44,1	159,2	172	31	18
13	1	40,5	155	127	23	1
13	1	56,6	165,7	122	15	1
13	1	42,3	150,3	109	34	13
13	1	50,5	166,8	134	6	1
13	1	44,4	153,5	160	25	24
13	1	47,3	160,9	170	25	10
13	1	43,7	152,5	181	30	31
13	1	34,2	148,7	164	22	7

13	1	39	160,5	156	18	10
13	1	44,3	147,3	155	22	16
13	1	59,1	169	190	25	30
13	1	48,3	167,3	142	30	20
13	1	42,8	149,4	134	23	8
13	1	46,8	159,8	135	19	12
13	1	42,6	154,1	135	24	12
13	1	49	172,8	209	13	19
13	1	57,7	169,4	186	32	12
13	1	55	162,2	223	33	27
13	1	49,9	162,5	178	37	18
13	1	33,6	147	133	29	3
13	1	41,6	145,5	177	33	26
13	1	53,7	163,5	172	36	16
13	1	48	161	139,3	24	18
13	1	48,1	156,8	139	23	17
13	1	41,4	150,8	170	30	13
13	1	42,8	153,1	148,8	31	17
13	1	43,9	153	164	26	8
13	1	57,9	179	204	21	16
13	1	59,2	165,3	189	31	24
13	1	38	146,4	154	27	9
13	1	55,1	172,1	155	3	16
13	1	41,5	161,8	154	37	15
13	1	51,7	155,3	202	38	25
13	1	62,1	169	130	19	2
13	1	47,5	169,2	187	26	10
13	1	45	169,8	199	33	13
13	1	59,4	175	176	34	15
13	1	57,1	160,1	130	25	3
13	1	52,8	164,2	152	25	13
13	1	56,1	163,3	131	22	3
13	1	56,5	172	170	31	21
13	1	57,9	168,6	186	35	20
13	1	61,9	166,2	87	30	2
13	1	39,5	157	140	19	12
13	1	53	170,6	180	17	20
13	1	50,2	156,3	144	13	6
13	1	46,4	168	153	22	1
13	1	41,2	159,2	149	18	10
13	1	44,1	148,4	186	18	19
13	1	36,3	150,3	135	25	19
13	1	37	142,3	145	28	10
13	1	64,6	173,3	168	33	15
13	1	51	158,7	152	17	10
13	1	48,2	161	180	27	9
13	1	37,3	148,7	161	35	9
13	1	31,8	150	169	26	10
13	1	47	151	172	24	15
13	1	58,4	164,9	138	35	7

13	1	55,9	167,5	152	25	20
13	1	46,8	167,5	203	47	22
13	1	39,3	145,3	126	34	7
13	1	53	165,4	145	6	13
13	1	53,1	169,1	193	37	19
13	1	47,7	163,2	195	25	22
13	1	49,6	157	150	21	8
13	1	47,1	146,6	144	30	1
13	1	54,3	167,1	181	25	20
13	1	42,8	154,2	168	28	14
13	1	35,2	146,1	138	23	18
13	1	51	151,9	153	28	11
13	1	59,8	176,2	201	35	11
13	1	54,9	159,5	146,5	21	6
13	1	55,6	171,6	181	33	10
13	1	42	144,3	160	18	21
13	1	46	164,5	142	36	20
13	1	42,8	162,1	183	20	7
13	1	48,8	167,4	164	19	13
13	1	59,7	172,6	175	15	16
13	1	47,3	161	140	21	2
13	1	59,1	178	130	27	10
13	1	43,7	150	166	26	20
13	1	50,5	168,1	163	20	30
13	1	51	159,2	126	31	8
13	1	72,8	161	143	22	10
13	1	79,8	170,2	178	17	10
13	1	99,2	170,7	113	25	1
13	1	91,1	177	153	23	2
13	1	78,3	166,8	125	13	1
13	1	64,8	165,5	140	32	3
13	1	47,9	139,9	130	16	16
13	1	74,7	168,5	129	25	9
13	1	47,5	139,1	132	23	4
13	1	64,8	161	175	32	12
13	1	70,5	168,6	145	20	8
13	1	63,6	167,9	144	7	15
13	1	63,9	164,3	135	17	3
13	1	45,4	142	156	28	14
13	1	55,6	154,5	155	7	11
13	1	59,3	160,4	168	20	15
13	1	61,5	161	140	12	2
13	1	63	166,4	215	40	30
13	1	76,5	171	143	27	5
13	1	67,5	172,4	185	41	19
13	1	80,7	183,1	110	27	10
13	1	81,2	173	183	30	30
13	1	59,6	156,1	133,4	28	30
13	1	64,5	167	145	25	4
13	1	43,1	153,4	131	30	5

13	1	34,7	135	165	29	29
13	1	39,1	143,5	150	20	21
13	1	41,7	158	164	15	18
13	1	34,2	145	166	36	7
13	1	30	136	150	33	7
13	1	46	153,8	150	28	19
13	1	63,1	170,5	194	15	3
13	1	36,3	148,5	157,8	29	10
13	1	38,3	149,3	150	23	10
13	1	47	146,7	139	30	4
13	1	44,5	150,8	134	33	40
13	1	63,2	180	170	31	1
13	1	39,7	154,8	133	12	11
13	1	55,1	177,5	172	10	9
13	1	39,2	150,5	163	40	16
13	1	42,5	156,3	167	36	15
13	1	51,4	153,6	200	35	20
13	1	40,7	153,3	133	16	1
13	1	39,1	152,5	186	21	10
13	1	48,6	165,1	159	25	14
13	1	49,5	166,4	120	20	5
13	1	49	162	152	34	6
13	1	45,8	153,2	123	18	3
13	1	38,2	157,8	135	26	11
13	1	55,6	163,2	181	31	15
13	1	45	151	141	28	4
13	1	54,1	167,6	105,1	32	2
13	1	56,6	160	134,4	32	7
13	1	47	164,3	133,5	28	20
13	1	54	167,2	168	21	27
13	1	40	155,2	152	31	8
13	1	34,4	140	122	15	17
13	1	54,6	168,4	134	13	10
13	1	45,3	156,2	150	28	4
13	1	48,8	168,2	140	17	12
13	1	41,1	146,3	147	27	5
13	1	47,4	154,5	141	29	10
13	1	49	160	162	24	34
13	1	49,8	167,2	169	23	21
13	1	44,8	167,2	154	23	15
13	1	51,8	165,2	174	21	6
13	1	69,3	157	134	29	8
13	1	100,5	166,1	135	20	1
13	1	83,1	172,5	123	15	2
13	1	108,4	176,3	152	34	2
13	1	58,5	155,2	123	23	14
13	1	74,1	171,1	160	27	8
13	1	58,9	160,8	135	27	17
13	1	68,2	174,3	172	29	1
13	1	70,5	174	123	13	9

13	1	74,4	170	180	43	8
13	1	61,3	163	151	25	10
13	1	65,9	167	140	30	12
13	1	58,3	160,4	152	10	1
13	1	41,2	149	139	25,5	4
13	1	48,1	161,1	112	16	9
13	1	47,1	155,2	149	19	9
13	1	41,9	149,2	147	16	13
13	1	47,1	167,5	164	24	18
13	1	43,8	156,6	118	27	5
13	1	64,8	173,8	195	28	25
13	1	36,7	155,1	172	25	20
13	1	53,4	152,4	128	17	4
13	1	58,7	151,9	144	34	26
13	1	36,6	136	149	28	16
13	2	54,4	164,5	105	36	6
13	2	60,6	165,5	140	37	12
13	2	48	155	119	36	6
13	2	51,4	164,2	125	24	17
13	2	55,1	159,4	163	23	27
13	2	54,7	155	120	26	10
13	2	52,2	159,3	110	20	13
13	2	37,2	157	112	25	8
13	2	60,3	159,5	96	29	12
13	2	48,8	160,3	99	32	2
13	2	43,7	158,9	103	28	10
13	2	40,4	153	137	21	8
13	2	37,1	151,5	130	21	7
13	2	51,5	153	105	18	2
13	2	51	162	137	30	10
13	2	32,4	144,8	140	39	23
13	2	49,8	156,7	140	35	12
13	2	41,3	154,2	156	30	9
13	2	38,8	151	120	18	20
13	2	50,6	165,8	142	31	25
13	2	49,3	164,1	102	12	2
13	2	58,1	170	156	29	30
13	2	43,1	160,8	135	29	9
13	2	56	168,3	120	30	2
13	2	47,3	163	148	36	2
13	2	45	148,7	146	10	1
13	2	53,7	154,3	108	27	10
13	2	53,3	162,1	84	31	28
13	2	43,7	154,5	124	27	10
13	2	45,3	151,5	132	31	12
13	2	37,7	154,9	133	11	19
13	2	47,5	164,5	115	25	12
13	2	49,7	164,2	114	25	19
13	2	86,3	171	113	24	11
13	2	76,2	157,6	115	25	5

13	2	63,5	161,2	135	32	5
13	2	65,7	165	108	25	6
13	2	68,6	157	131	31	15
13	2	62,8	161	150	38	33
13	2	56,8	152,2	122	31	15
13	2	75,6	164,8	71	19	5
13	2	46	155,6	118	25	8
13	2	35,2	153,2	119	12	5
13	2	50,1	163,3	104	32	10
13	2	40,3	159,2	156	30	4
13	2	35	153,8	125	28	26
13	2	51,4	160	121,1	17	20
13	2	45,4	165,5	118	21	5
13	2	37,8	152,5	170	42	30
13	2	42,1	153,5	81	10	10
13	2	44,1	161	101	27	10
13	2	39,2	148,1	96	20	8
13	2	41,9	157,6	132	28	7
13	2	46,4	158,1	120	24	10
13	2	38,8	145,5	100	23	5
13	2	38,6	156,5	162	31	11
13	2	38,3	155,2	137	32	15
13	2	47,8	159,9	122	28	15
13	2	46,6	168	127	29	6
13	2	44,1	156,1	114	21	3
13	2	35,4	150,1	77	20	6
13	2	44,6	151	165	26	13
13	2	50	169,3	110	30	20
13	2	46,7	162,5	130	32	2
13	2	61,1	166,5	119	18	7
13	2	50,8	159	62	30	5
13	2	59,1	172,5	78	30	15
13	2	46,7	160	124	29	5
13	2	38,9	159	117	15	6
13	2	43,3	152,3	110	36	1
13	2	43,6	146,2	103	22	6
13	2	43,6	161,1	103	26	9
13	2	45,1	160	146	22	8
13	2	51,2	160,7	119	13	10
13	2	53	162	100	20	1
13	2	53	164,3	124	30	13
13	2	46,7	159	131	29	13
13	2	39,6	148,3	139	20	13
13	2	50,2	161,3	135	18	29
13	2	46,9	153,5	119	20	3
13	2	42	156	122	25	4
13	2	41,1	157,5	143	30	8
13	2	52,1	160,7	112	25	3
13	2	46	163	115	26	1
13	2	44,4	149	110	25	15

13	2	50,2	152	130	41	17
13	2	53,2	161,5	100	35	16
13	2	43,8	154,2	115	19	8
13	2	52,5	157,4	108	23	12
13	2	41,7	157	123	32	17
13	2	54,6	166	145	13	5
13	2	43,2	157	135	40	10
13	2	62	163,5	147	24	29
13	2	46,8	161,2	108	21	10
13	2	50,3	154,3	104	31	6
13	2	44,7	162,8	75	34	3
13	2	40,3	154,1	143	35	8
13	2	46,2	160,1	144	25	10
13	2	33,9	151	114	25	19
13	2	48,5	164,5	118	21	10
13	2	49,3	159,8	132	28	12
13	2	51	157,6	110	25	8
13	2	42,9	156	125	28	16
13	2	51,3	167	125	27	10
13	2	52,8	153,1	120	25	8
13	2	42,8	156,5	128	25	6
13	2	51,8	166,2	122	28	15
13	2	44,2	153,4	84	18	10
13	2	44,8	163	107	22	1
13	2	51,5	153,5	130	33	12
13	2	39,2	150,6	101	26	5
13	2	38,5	160,2	121	21	30
13	2	59,8	160,2	103	26	2
13	2	51,8	161,8	92	14	7
13	2	46,8	155,4	121	26	10
13	2	41,9	163,5	134	23	10
13	2	42,8	162,9	130	20	15
13	2	45,3	155	108	27	6
13	2	58,6	165,6	84	18	10
13	2	60,8	162	114	31	8
13	2	54,6	160,8	124	33	41
13	2	48,2	159,5	120	1	1
13	2	57,2	166,2	110	29	8
13	2	58,9	162	108	16	18
13	2	53,5	168,2	102	13	9
13	2	59	167,1	120	30	6
13	2	48,5	161,2	105	26	12
13	2	52,3	167,8	123	17	11
13	2	53,8	172	107	26	1
13	2	52,2	161	115	26	12
13	2	31,5	155	167	23	9
13	2	38,1	140	137	29	29
13	2	49,4	154	170	33	39
13	2	52,5	161,1	113	25	9
13	2	55,2	169	114	11	3

13	2	42,4	158	139	24	13
13	2	50,8	161,2	89	26	8
13	2	46,2	153,3	133	34	14
13	2	53,5	167,2	137	34	40
13	2	60,5	165,2	145	49	16
13	2	57,2	167	53	21	6
13	2	50,5	160,2	123	25	11
13	2	49,4	163,2	105	18	4
13	2	81	163	123	32	14
13	2	88,1	165,5	130	29	6
13	2	69,6	154,5	105	22	3
13	2	77,5	158	101	37	2
13	2	75,5	160	109	31	20
13	2	83,8	165	105	17	10
13	2	66,3	167,1	82	19	11
13	2	57,4	152	123	27	15
13	2	65,4	159	99	25	11
13	2	62,1	163,8	82	32	5
13	2	56,7	156	123	26	6
13	2	65,1	161	134	30	6
13	2	71,2	163,1	120	29	7
13	2	61,6	161,2	156	25	20
13	2	54,5	149	118	31	13
13	2	78,2	172,4	150	34	10
13	2	71,1	164,3	101	32	24
13	2	71,9	162,8	83	19	14
13	2	73,6	164,1	140	27	3
13	2	71,4	166,1	86	34	11
13	2	60,3	163,3	110	25	7
13	2	43,6	152,4	161	32	17
13	2	48,2	158,5	121	29	12
13	2	45,8	144,1	112	23	8
13	2	44,3	155,8	106	28	8
13	2	37,2	155,4	107	27	7
13	2	42,8	156,3	118	29	7
13	2	43,7	158,1	150	35	12
13	2	50,3	148	145	30	22
13	2	41,1	149,2	131	27	4
13	2	45,6	157,2	120	29	10
13	2	52,7	156,5	130	28	4
13	2	61,7	167,2	72	26	8
13	2	48,2	153,4	180	28	13
13	2	52	159,3	74	19	4
13	2	58	167,5	138	32	8
13	2	42,1	160	108	19	2
13	2	42,9	151,3	113	27	12
13	2	58,7	169,2	111	5	4
13	2	47,2	154,2	136	25	5
13	2	49,5	153	116	32	11
13	2	39,9	152,8	130	15	10

13	2	53,6	162,3	127	20	8
13	2	43,7	165,7	122	25	18
13	2	59	164,5	127	20	2
13	2	42	155	131	24	9
13	2	47,3	158,9	105	24	5
13	2	50,8	154	142	38	20
13	2	42	157,8	115	13	2
13	2	47,5	164	118	23	8
13	2	48,1	158	77	31	9
13	2	53,6	161,2	123	12	3
13	2	39,5	158,9	143	26	12
13	2	46,2	156,2	127	40	12
13	2	54,4	160,8	70	29	6
13	2	53,9	166,1	69	13	10
13	2	44,8	150	152	33	27
13	2	36,9	149,5	138	31	14
13	2	40,8	150,2	138	19	11
13	2	54	158,5	124	35	8
13	2	47,9	151,3	121	25	12
13	2	52	159	163	32	17
13	2	56,5	164	135	33	23
13	2	36,5	150,5	140	24	1
13	2	46,3	156,5	133	32	11
13	2	40,5	156,8	129	35	14
13	2	50,9	160	120	22	10
13	2	29,3	144,8	130	35	13
13	2	48,3	166	126	36	33
13	2	54,5	161,5	118	19	8
13	2	41,2	157	109	35	9
13	2	29,5	142	133	37	1
13	2	50	156,2	131	31	2
13	2	43,9	147,9	89	26	14
13	2	43,1	163	120	22	14
13	2	46,1	158,2	109	21	8
13	2	54	160	114	30	1
13	2	36,7	157	120	25	4
13	2	55,3	159,5	158	15	13
13	2	50,2	166	177	26	30
13	2	42	161,6	140	24	10
13	2	40,8	153,6	148	22	12
13	2	52,6	160	123	22	16
13	2	45,2	165,2	100	22	5
13	2	46,7	153	122	17	27
13	2	42,4	160,8	134	15	17
13	2	57,8	160	108	28	9
13	2	38,4	158,5	82	15	5
13	2	54,3	155	126	27	10
13	2	45,6	161,5	160	16	7
13	2	49,2	156,5	110	20	4
13	2	42,4	157,6	142	22	21

13	2	64,7	168,4	105	32	8
13	2	57	166,7	168	40	18
13	2	35,4	157,2	150	18	14
13	2	44,7	155,4	147	20	20
13	2	66,2	173,4	121	34	1
13	2	50,7	165,2	107	23	6
13	2	44,3	165,7	130	27	9
13	2	46,3	148,5	133	30	28
13	2	55,2	168	110	14	16
13	2	48,6	154,6	121	23	6
13	2	57	159,4	95	32	1
13	2	40,7	152	169	31	36
13	2	55,1	160,5	160	26	2
13	2	43,1	150,1	96	29	8
13	2	50,9	155	150	33	30
13	2	54	162,1	106	29	9
13	2	37,2	156	140	23	2
13	2	45,3	154	173	42	25
13	2	49,2	150,1	140	22	2
13	2	52,7	160	162	38	10
13	2	52,1	157,5	81	18	13
13	2	57,9	172,3	159	29	9
13	2	86	165,2	106	23	10
13	2	71,2	153,9	93	32	4
13	2	127,1	169	81	5	1
13	2	65,1	147,5	93	18	2
13	2	60,2	157,5	95	20	1
13	2	63,2	157,6	107	26	7
13	2	60,1	160,3	144	30	10
13	2	64,4	164,8	147	32	12
13	2	57,2	153,1	81	10	4
13	2	78,6	175,4	133	33	10
13	2	60,6	157,5	137	31	5
13	2	69,8	169,1	107	16	3
13	2	72,8	163,3	115	24	9
13	2	70	157,1	99	21	9
13	2	63,1	163,1	95	23	1
13	2	61,7	159,3	110	29	30
13	2	61	153,4	170	34	23
13	2	58,5	156,2	97	19	8
13	2	67,8	154,2	100	24	9
13	2	65	160	80	40	20
13	2	62,5	150,4	105	31	5
13	2	66,4	159	123	39	7
13	2	71,8	169	104	15	3
13	2	69,7	165,5	90	19	1
13	2	63,2	158,2	115	24	10
13	2	59,4	156,8	114	34	1
13	2	54,2	158,5	117	30	14
13	2	52,5	165,9	130	22	10

13	2	43,1	153,2	108	25	3
13	2	43,1	157,7	127	22	8
13	2	45	151,5	96,8	21	3
13	2	50,5	159,5	107	27	18
13	2	39,3	153,3	118	18	7
13	2	55,5	161,1	140	36	7
13	2	39,5	156,2	177	36,5	6
13	2	42,2	160,4	166	32	6
13	2	50,6	159,5	158,9	24	25
13	2	49,3	162,1	143	37	20
13	2	50	148,2	134	32	12
13	2	46,3	159	116,1	23	2
13	2	48,2	152,5	112	29	12
13	2	35,2	145,8	146	26	9
13	2	44,2	165,2	132	21	12
13	2	47	166,2	128	24	11
13	2	47,3	152,8	119	25	9
13	2	43,1	160,2	150	29	31
13	2	49,6	154	151,2	32	16
13	2	49,3	156	95	31	10
13	2	49,3	158	130	27	15
13	2	59,3	164,2	105	21	5
13	2	42,9	158,7	162	31	13
13	2	51,3	157,1	118	28	1
13	2	47,8	161,2	130	3	15
13	2	53,5	158,3	110	23	5
13	2	54	156,5	116	25	5
13	2	54,4	163,4	148	14	2
13	2	46,1	154,9	94	17	5
13	2	42,1	154,3	101	25	2
13	2	49,8	159,4	108	28	30
13	2	43,6	158,4	150	26	12
13	2	50,2	164,5	128	22	8
13	2	49	160,4	134	14	15
13	2	51,2	159	124	32	20
13	2	78	165,2	94	33	3
13	2	75,5	159	115	25	6
13	2	86	158,2	96	39	5
13	2	60,9	161,6	96	14	10
13	2	65	164	120,5	34	15
13	2	72,5	170,4	102	28	10
13	2	60,9	159,2	103	22	7
13	2	63,1	158,6	108	31	12
13	2	60,3	155,3	125	28	11
13	2	48,1	165,5	105	21	5
13	2	54,4	162,8	133	28	15
13	2	49,3	152	129	27	2
13	2	39,8	147,6	120	22	26
13	2	36	151,9	157	27	41
13	2	59	163	108	27	7

13	2	44,7	148	80	15	2
13	2	53,5	163	112	29	12
14	1	54	176,4	142	13	9
14	1	60,7	173	163	21	12
14	1	49,5	167	178	12	19
14	1	62,8	172	212	31	21
14	1	67,7	180,9	215	31	22
14	1	48,6	170,8	173	30	7
14	1	67,7	179,2	199	25	5
14	1	62,5	165,5	125	28	10
14	1	56,7	166,6	156	34	14
14	1	58	166	206	31	20
14	1	50,1	175,6	190	25	12
14	1	67	165,3	182	32	13
14	1	85,1	183,5	180	29	1
14	1	74,9	175,7	158	38	6
14	1	72,4	168,5	177	32	15
14	1	41	147	170	32	30
14	1	61,5	171	182	30	16
14	1	44,2	167	130	20	18
14	1	57,5	163,5	147	25	6
14	1	54,3	164,8	168	27	21
14	1	57,8	166,8	171	34	15
14	1	47,2	163,1	182	29	19
14	1	44,5	153,5	170	21	20
14	1	50,9	166,1	160	15	9
14	1	42,8	164	185	14	23
14	1	58,2	170	170	25	18
14	1	56,1	173,5	161	27	1
14	1	61,8	182	182	7	22
14	1	52,1	163	143	16	5
14	1	52,3	170,1	172	25	16
14	1	53,6	177,5	179	13	8
14	1	57,8	164,5	184	28	26
14	1	67,6	180	179	31	20
14	1	48,2	153,4	175	31	30
14	1	41,4	159,6	162	25	5
14	1	57,2	178,9	185	23	18
14	1	47,3	160,8	160	23	11
14	1	60,6	171,4	164	9	16
14	1	46,5	162	125	28	8
14	1	46	159,5	160	34	15
14	1	45,5	163,5	142	27	18
14	1	51,5	164,7	159	39	15
14	1	56,1	176,1	180	25	22
14	1	52,8	173,2	193	34	3
14	1	56,7	167,2	185	29	31
14	1	51	167,4	144	13	15
14	1	48,2	170,3	158	27	17
14	1	59,1	166	196	41	18

14	1	49,9	172,8	186	29	20
14	1	79,7	164	111	26	5
14	1	87	171,6	153	13	9
14	1	104	166,3	140	16	1
14	1	70,2	157,5	125	21	8
14	1	86,1	158,8	109	15	6
14	1	83,8	171,4	172	25	14
14	1	79,6	180	118	19	1
14	1	76,4	169,2	130	35	10
14	1	75,3	165	143	27	3
14	1	53,4	168,3	157	15	2
14	1	57,6	173,5	178	14	20
14	1	48,6	167	174	31	26
14	1	47,1	163,2	125	21	7
14	1	49,4	163	163	25	10
14	1	53,8	165,6	119	17	10
14	1	38	154,2	147	27	19
14	1	56,1	168,2	182	28	25
14	1	53,3	172,6	234	37	14
14	1	42,6	153,4	191	26	1
14	1	53	163,2	157	28	15
14	1	57,1	172,5	166	31	18
14	1	53,4	167,5	210	25	30
14	1	44	161	130	19	11
14	1	41,3	157,9	144	21	11
14	1	53,3	174,5	218	30	40
14	1	46,5	154,2	128	20	2
14	1	42,6	161,8	180	20	19
14	1	43,6	158,7	142	26	25
14	1	38,4	147,5	155	34	2
14	1	46,7	164,3	119,5	13	3
14	1	67,3	172,8	195	31	27
14	1	42	154,5	158	25	12
14	1	62	181	186	19	1
14	1	64,9	169,1	183	33	4
14	1	50,3	163	147	19	17
14	1	45,2	166,3	170	19	26
14	1	61,1	168,5	174	31	15
14	1	60,1	178,5	184	28	20
14	1	59,6	171,2	136	28	12
14	1	38,5	147,7	142	23	15
14	1	50,8	163,5	198	34	25
14	1	55,5	174,5	172	28	21
14	1	48,7	170,9	171	32	10
14	1	58,2	171,2	150	24	15
14	1	50	167,2	177	25	10
14	1	52,2	172,7	182	21	4
14	1	55	173,2	150	28	5
14	1	55	172	161	20	15
14	1	44,9	170,6	153	10	16

14	1	46,4	159,8	164	28	30
14	1	67	180,4	162	32	10
14	1	57,5	176,7	157	27	10
14	1	44,1	159,3	174	27	15
14	1	49,6	168,5	208	21	14
14	1	61,6	174,4	110	28	3
14	1	42	161,5	186	20	16
14	1	65,5	179,5	200	5	15
14	1	65,1	177	206	34	21
14	1	68,4	179,1	200	26	10
14	1	49,6	170,1	156	21	18
14	1	67,3	180,2	193	29	27
14	1	61,5	170,1	147	6	15
14	1	55,7	158,5	175	32	20
14	1	41,3	152,5	163	29	6
14	1	61,4	168,3	163	12	12
14	1	73,2	179,5	182	25	18
14	1	56,2	172	190	23	15
14	1	48,7	167	189	25	39
14	1	54,2	172,8	200	26	15
14	1	43,6	156,6	160	27	15
14	1	45,3	162	135	32	2
14	1	58,9	173,2	163	9	17
14	1	38,4	148,1	192	14	15
14	1	68	174,9	160	19	20
14	1	59,6	180,5	204	16	12
14	1	74,3	186,9	210	34	11
14	1	53,2	168	172	29	27
14	1	71	188,6	170	24	9
14	1	45,8	164	150	33	5
14	1	51,5	166,1	173	28	25
14	1	50,6	165	184	20	10
14	1	50,2	165,1	143	11	5
14	1	44,8	161,5	140	20	8
14	1	59,8	173,2	164	20	19
14	1	50	165,7	204	26	19
14	1	42,1	153,4	161	22	21
14	1	49,3	160,3	110	16	4
14	1	66,9	180,8	215	28	29
14	1	56,5	170,9	167	25	20
14	1	59,8	182	140	27	3
14	1	61,5	167,6	164	33	8
14	1	63,6	183	181	18	25
14	1	63,4	182,7	149	14	19
14	1	49,8	165,5	172	39	20
14	1	54,5	175	145	28	7
14	1	48	168,8	133	15	30
14	1	41,7	155,8	193	27	6
14	1	48	153,5	164	30	26
14	1	69,4	175,2	149	27	19

14	1	49,4	161,6	149	19	12
14	1	53,9	169	190	34	20
14	1	44,5	161,8	195	26	40
14	1	55,5	162,3	153	28	6
14	1	60,6	173,9	190	35	15
14	1	98,4	178,3	150	18	6
14	1	96	172,8	96	13	1
14	1	85,9	171,5	156	34	9
14	1	88,5	172	153	28	5
14	1	61,1	146,1	148	27	10
14	1	79,5	165,8	170	36	5
14	1	68,5	172,1	136	24	3
14	1	68,3	169	181	35	13
14	1	63,4	166	176	39	20
14	1	81,5	185,7	169	34	4
14	1	80,1	174,7	161	19	9
14	1	81,8	176,1	177	26	20
14	1	82,5	177	116	20	19
14	1	74,4	174	161	36	4
14	1	79,7	176	130	29	4
14	1	70,1	170,4	145	26	17
14	1	79,3	180,1	180	22	20
14	1	81	171	163	37	49
14	1	63	162,3	130	35	13
14	1	73,6	171,1	185	39	25
14	1	56,6	163,2	182	31	13
14	1	56,3	175,4	200	33	10
14	1	38,9	152,2	189	27	20
14	1	52,1	165,1	143	15	20
14	1	52,3	167,2	153	20	11
14	1	53,8	167,5	185	32	10
14	1	36,7	140,6	154	24	20
14	1	51,4	158,6	146	20	20
14	1	40,2	155,3	150	22	2
14	1	52,7	161	173	21,5	11
14	1	49	177,9	187	30	7
14	1	41,2	160,2	111	31	9
14	1	47	160,5	140,4	28	17
14	1	51,3	159,5	161	22	8
14	1	44,5	154,6	194	31	30
14	1	64	168,3	107	19	1
14	1	55,6	172,1	177	22	10
14	1	50,6	168,9	177	28	11
14	1	57,3	160,2	123	13	10
14	1	45,6	157	162	20	9
14	1	66,3	175,1	169	40	9
14	1	48,9	168,6	171	35	10
14	1	58,5	172,1	152	20	15
14	1	50	162,8	165	15	12
14	1	51,2	172	175	22	15

14	1	42,4	152	172	28	4
14	1	51,1	152,3	156	13	9
14	1	55,2	163	168	31	22
14	1	53,3	169,2	166	28	11
14	1	49,5	170	150	28	20
14	1	62,5	174,6	89	14	13
14	1	56	169	147	20	8
14	1	43,2	160,2	190	17	6
14	1	43,2	158,9	155	26	13
14	1	35,5	159,6	177	24	18
14	1	50,1	170,3	158	30	16
14	1	51,5	168	147	20	10
14	1	52,8	177,9	169	26	6
14	1	71,1	186	170	20	10
14	1	47,5	165,5	175	26	19
14	1	49,3	175,5	172	30	25
14	1	60,5	169	160	9	10
14	1	49,2	168,7	110	21	2
14	1	48,1	163,2	165	21	10
14	1	52,1	167,2	136	18	2
14	1	56,4	162,1	103	26	1
14	1	58,5	171,6	204	31	20
14	1	45,6	161,3	190	21	13
14	1	54	166	163	21	10
14	1	58,9	165,5	181	26	30
14	1	62,2	168,4	137	31	3
14	1	46,9	170,3	196	42	22
14	1	45,7	167,6	186	11	8
14	1	43,2	164,6	159	12	13
14	1	47,4	166,2	110	13	5
14	1	44,8	160,3	145	23	18
14	1	60,1	162,5	174	37	20
14	1	44,8	158	145	21	19
14	1	65,2	170,3	123	17	6
14	1	64,5	178,9	115	27	9
14	1	36,4	156,1	145	21	30
14	1	51,1	160,6	181	29	26
14	1	43,5	159,2	152	28	9
14	1	45,3	160	159	29	11
14	1	72,8	183,5	230	21	25
14	1	61,1	176,2	162	32	6
14	1	53,2	158,9	158,5	18	10
14	1	54,2	166,2	172	26	6
14	1	64,2	173,2	160	28	1
14	1	51,1	154,7	172	24	21
14	1	70,5	182,3	232	32	13
14	1	51	161,7	158	26	12
14	1	44,7	157,5	130	25	10
14	1	49,3	163,2	149	19	17
14	1	62,5	166,5	169	14	17

14	1	83	162,5	150	28	25
14	1	88,1	169,5	169	14	10
14	1	142	179	90	12	1
14	1	55,2	154,2	139	25	12
14	1	61	161,8	180	29	22
14	1	68,1	167,5	176	31	21
14	1	69,4	161,5	162	26	14
14	1	90	180	149	4	2
14	1	86,6	180,2	157	31	14
14	1	76,5	173,2	180	32	25
14	1	58,2	157,3	163	18	12
14	1	63,6	163,8	169	28	12
14	1	40,4	157	165	16	12
14	1	47,5	160,8	154	30	16
14	1	39,7	147,5	112	34	26
14	1	45,5	152,3	133	21	22
14	1	49,9	155,2	152	34	3
14	1	42,9	155	155	29	9
14	1	48,1	164,6	186	32	18
14	1	58,1	165,5	143	15	1
14	1	43,4	156,8	115	25	8
14	1	44,3	151,4	131	23	8
14	1	45,6	151,4	159	32	8
14	1	46,1	158	174,5	25	15
14	1	53,8	163,1	120	23	10
14	1	48,8	158,1	169	32	15
14	1	37,8	155,8	158	18	8
14	1	58	179,3	195	31	29
14	1	41,2	154,5	184	20	32
14	1	49,3	165,3	207	31	3
14	1	55,3	163,4	160	18	15
14	1	44,8	160,1	130	17	10
14	1	50,5	159,3	172	34	21
14	1	43,6	159,1	159	26	4
14	1	55	162,5	164	23	5
14	1	53,3	169,2	168	25	18
14	1	57,9	169,8	153	14	10
14	1	54,1	159,1	155	35	8
14	1	68	179	194	18	12
14	1	36,8	147,5	158	23	10
14	1	49,9	162,2	209	26	30
14	1	43,5	161,3	136	6	14
14	1	40,3	166,1	160	27	3
14	1	56,3	174,9	184	32	20
14	1	38,1	145,8	158	29,5	10
14	1	55,4	173,8	219	32	2
14	1	69,7	180,1	222	20	10
14	1	52,7	176,9	197	31	25
14	1	50,6	161,5	161	25	20
14	1	41,4	161,5	119	25	8

14	1	60,2	169,7	191	25	26
14	1	54,2	155,5	178	32	30
14	1	44,7	155,6	132	31	20
14	1	54,3	176,1	140	1	10
14	1	53,1	168,1	200	29	24
14	1	60,1	179,6	193	33	15
14	1	46,2	169	191	23	8
14	1	50,1	166,4	202	33	19
14	1	81,6	164,6	185	31	29
14	1	73,3	167	150,2	25	2
14	1	63,8	164,6	150	28	20
14	1	62,6	163,7	180	19	1
14	1	73,5	166,8	169	16	4
14	1	81,9	177,4	150	18	9
14	1	77	177,5	146	42	4
14	1	89,1	180	141	34	2
14	1	75,2	179,3	164	35	1
14	1	82	181,5	177	13	20
14	1	66,3	172,5	169	10	3
14	1	39,2	152,5	155	33	7
14	1	51,9	159,7	187	27	20
14	1	57,6	160,8	192	25	30
14	1	58,1	164	204	30	17
14	1	54,1	173,4	195	24	16
14	1	50,4	163,3	188	20	8
14	1	38,6	147	170	26	21
14	1	38,8	152,8	147	24	24
14	1	85,1	174	153	29	30
14	2	45,1	156	107	25	27
14	2	60,5	165,3	122	17	6
14	2	47,5	162	159	41	3
14	2	56,1	158,5	109	23	18
14	2	55,7	165,1	105	19	9
14	2	48,5	156,4	101	11	22
14	2	66,2	172,5	125	15	2
14	2	48,2	159	83	23	6
14	2	53,8	160	128	11	11
14	2	75,2	169,8	116	24	14
14	2	54,2	172,3	125	18	6
14	2	51,2	151,5	109	22	12
14	2	43,9	160,5	80	23	29
14	2	47,6	155	120	32	16
14	2	50,7	163,6	155	23	16
14	2	50,2	155	97	41	29
14	2	42,6	159,2	115	26	10
14	2	52,5	159,5	153	23	7
14	2	52,7	156,3	103	26	21
14	2	49,9	165,1	129	24	5
14	2	57,1	162,3	105	31	12
14	2	53,5	162,4	134	14	27

14	2	32,6	152,8	139	18	16
14	2	58,2	164,1	127	23	5
14	2	49,8	162,9	103	16	5
14	2	64,5	168	164	41	29
14	2	46,6	171,3	90	22	5
14	2	50,3	164,2	125	29	12
14	2	50,7	158	114	32	22
14	2	51,8	160,7	132	30	13
14	2	61,4	168	144	36	32
14	2	42,5	155	155	34	20
14	2	43,7	162,2	81	36	12
14	2	53,5	162	82	33	24
14	2	49,6	165,3	125	1	6
14	2	54,7	165,5	124	17	3
14	2	54,1	174,8	123	29	10
14	2	51,8	171,3	130	28	17
14	2	52,9	167,5	150	29	19
14	2	46,3	156,1	103	27	5
14	2	55,2	166	126	17	13
14	2	53,8	159,5	118	19	8
14	2	55,9	164,5	94	37	8
14	2	45,8	156,2	102	19	3
14	2	88,4	166	143	27	11
14	2	68,1	164,1	110	28	10
14	2	70,2	156	84	34	5
14	2	62,9	161	126	30	20
14	2	74,9	171,5	116	23	13
14	2	37,6	151,6	125	36	16
14	2	33,7	154,5	116	22	8
14	2	33,6	149,8	114	25	13
14	2	54,5	156,6	109	18	5
14	2	50,4	158,1	143	33	33
14	2	48,8	165	103	17	11
14	2	47,5	154	120	22	22
14	2	56,1	154,8	110	37	8
14	2	48,3	166,1	110	28	5
14	2	41,4	160,7	123	29	12
14	2	46,3	163	140	27	23
14	2	63,7	164,7	101	10	2
14	2	48,1	166	152	28	31
14	2	44,8	152,3	150	34	14
14	2	48	161	154	27	21
14	2	47,8	160	143	28	11
14	2	45,3	159,5	131	31	23
14	2	45	154,5	135	18	20
14	2	43,6	147,5	115	27	20
14	2	56,8	156,5	138	33	9
14	2	47,5	162,8	75	32	20
14	2	60,2	163,2	158	42	20
14	2	55,8	163,5	113	18	4

14	2	51,8	175	129	1	4
14	2	53,3	156,7	114	15	3
14	2	47,2	161,2	125	29	15
14	2	40,4	153	140	27	10
14	2	51	163,5	145	30	4
14	2	48,2	155	133	46	30
14	2	42,5	163,9	126	21	5
14	2	41,6	162,4	134	34	8
14	2	46,3	156,1	129	34	16
14	2	50,1	157,9	122	42	11
14	2	44,7	168,5	129	28	7
14	2	53,5	153,5	112	20	5
14	2	63,6	167,5	74	32	7
14	2	42	156,4	140	17	31
14	2	49,2	167,2	130	35	5
14	2	45,8	157,5	135	26	10
14	2	46,9	162,5	123	25	13
14	2	53,7	163	137	29	13
14	2	53,2	158,2	165	39	27
14	2	53,6	168	126	23	12
14	2	58,1	172,8	101	30	5
14	2	56,7	168,2	132	25	9
14	2	70,4	172	140	30	2
14	2	54,4	158,9	122	28	4
14	2	51,2	158,2	109	26	8
14	2	32,2	148	148	36	21
14	2	59,8	164	153	27	18
14	2	60,2	175,5	68	30	27
14	2	49,5	161,5	144	18	15
14	2	49,4	154,6	143	31	14
14	2	54	168	132	29	12
14	2	40	164,2	130	18	10
14	2	43,4	157,6	125	17	12
14	2	53,6	161,5	145	23	12
14	2	53,2	158,5	111	26	7
14	2	48,2	164,2	108	21	12
14	2	52	161	111	16	10
14	2	47,1	165,1	113	27	4
14	2	46,5	158,7	108	27	21
14	2	41,7	156,5	128	18	12
14	2	52	155,2	115	21	12
14	2	64,6	165,1	131	32	15
14	2	44,3	159,5	94	25	4
14	2	54,3	159,5	102	31	25
14	2	48,5	162,8	138	31	12
14	2	67,3	169,5	86	21	5
14	2	42,3	155,2	105	24	8
14	2	60,1	162,3	182	27	9
14	2	49,8	159	138	32	18
14	2	40,2	155,5	128	28	12

14	2	56,5	162,5	142	34	9
14	2	47,7	157,5	164	37	41
14	2	48,6	170	134	20	10
14	2	57	159,8	87	25	13
14	2	51,7	164,5	138	29	9
14	2	67,9	169	144	28	10
14	2	79,8	167	96	31	3
14	2	82,6	153	128	27	30
14	2	77,7	156,2	150	32	27
14	2	80,3	165,2	66	13	1
14	2	85,4	159	96	26	1
14	2	55,6	150	127	29	20
14	2	62	160	124	28	14
14	2	65	163	116	32	6
14	2	55,4	153	140	28	10
14	2	75,1	169,5	103	14	16
14	2	67	159,1	137	37	8
14	2	63,1	161,5	105	30	22
14	2	70	164	130	28	29
14	2	69	167,2	130	12	11
14	2	76	163	112,5	22	13
14	2	58,3	148,2	112	32	8
14	2	81,4	170,3	120	31	8
14	2	68,4	168,3	95	23	8
14	2	53,4	155,3	97	26	10
14	2	47,8	157,6	99	39	5
14	2	43,4	158	96	27	24
14	2	51,3	167,5	123	17	3
14	2	44,2	164,9	130	30	16
14	2	49,5	161,6	130	26	1
14	2	55,5	168,5	143	22	8
14	2	53,1	160,4	150	28	13
14	2	43,6	150,7	154	30	20
14	2	43,5	155,1	158	28	12
14	2	55,2	164,3	133	37	9
14	2	52,6	158,1	75	28	12
14	2	49,8	162,7	106	27	2
14	2	41	148,5	130	41	25
14	2	57,1	162	114	27	10
14	2	45,7	153,9	95	36	25
14	2	44,2	157,5	124	27	14
14	2	51,3	163,5	140	22	7
14	2	57,1	165	137	27	1
14	2	55,4	171,5	149	29	17
14	2	52,4	165,2	143	26	12
14	2	47,2	159,5	130	27	11
14	2	51	156,2	105	18	7
14	2	57,1	160,5	105	14	11
14	2	48,3	160	122	32	10
14	2	55,2	155	130	29	8

14	2	46,9	154,2	117	36	8
14	2	54,6	164,5	80	21	4
14	2	43,6	159,5	175	28	13
14	2	54,7	157,6	170	41	15
14	2	44,6	154,2	173	34	10
14	2	54,3	157,8	120	34	13
14	2	45,2	160,7	111	27	16
14	2	49,1	165,8	84	32	6
14	2	58,7	160	147	29	15
14	2	60,9	162,1	80	31	5
14	2	50,1	163,6	144	27	23
14	2	47,2	152,5	104	22	17
14	2	62,4	164	141	28	17
14	2	47,8	159,4	107	22	20
14	2	54,4	157,2	117	37	12
14	2	57,7	162,1	132	34	15
14	2	52	158,2	131	37	5
14	2	48,1	160	113	29	5
14	2	52,9	159,9	115	33	17
14	2	46,7	166	111	35	2
14	2	52,3	169,2	136	24	13
14	2	59,1	167,1	100	24	13
14	2	58,6	161,5	121	17	10
14	2	54,6	164,1	89	12	1
14	2	49,5	165,5	70	28	3
14	2	58,5	164,2	164	19	18
14	2	48,6	154,7	135	23	12
14	2	55,7	168,3	118	29	15
14	2	33,3	155,2	130	29	10
14	2	65	166	141	25	1
14	2	54,3	166,8	124	29	9
14	2	49,7	157,2	130	29	12
14	2	53,9	162,3	87	14	6
14	2	48,2	155,8	165	28	21
14	2	47,5	161,5	113	32	14
14	2	44,4	160,3	121	22	2
14	2	53,3	162	120	30	35
14	2	53,1	166	130	20	11
14	2	47,3	164,1	124	28	7
14	2	40,3	146,8	109	16	4
14	2	52,3	155,8	121	43	1
14	2	45,4	160	142	23	10
14	2	49,1	156,3	158	22	15
14	2	59	170,5	143	39	12
14	2	51,5	149,6	101	24	20
14	2	51	160	133	36	10
14	2	55,8	159,3	102	23	12
14	2	43,9	159,2	131	31	10
14	2	54,9	166,1	164	30	15
14	2	51,5	164,3	119	22	12

14	2	48,8	160,2	128	36	24
14	2	46	161	132	26	4
14	2	89,8	169,2	100	38	17
14	2	97,1	157	83	25	2
14	2	94,2	164,8	123	25	8
14	2	85,2	162,9	100	16	2
14	2	77,2	161	100	40	1
14	2	69,6	164,8	141	33	5
14	2	64,4	158,2	110	27	2
14	2	58,4	153,8	89	14	2
14	2	59,1	157,2	109	23	19
14	2	64,5	153,8	89	23	8
14	2	59,1	157,8	117	28	10
14	2	59,6	154,1	100	23	2
14	2	64	158	93	24	5
14	2	78,6	169,2	100	19	4
14	2	68,3	160,2	130	22	11
14	2	80,7	170,3	92	23	9
14	2	60,3	158,7	124	32	20
14	2	73,1	164,2	108	29	6
14	2	62,8	152	98	29	6
14	2	73	166	90	34	1
14	2	65,3	163,6	110	30	12
14	2	70,7	171,6	150	35	7
14	2	74,3	173,5	156	22	1
14	2	71,5	170,4	138	32	12
14	2	71,1	157,6	120	29	18
14	2	72	163,1	108	29	4
14	2	79,9	169,3	101	19	8
14	2	35,8	154,7	95	26	3
14	2	37,2	152,3	105	25	5
14	2	44,6	162,7	137	34	8
14	2	41,3	166,1	124	22	18
14	2	43,1	160,3	133	24	9
14	2	45,3	153,6	120	22	19
14	2	40,7	154,6	134	24	20
14	2	40,6	153,5	163	33	20
14	2	57,2	158,5	113	30	15
14	2	37,1	152,8	86	20	5
14	2	64	165,5	104	31	10
14	2	57,5	163,5	143	25	7
14	2	45,2	159,1	93	28	13
14	2	42	153,2	138	9	12
14	2	61,5	162,3	138	23	30
14	2	49	158	110	29	10
14	2	47,7	164,2	126	34	10
14	2	41,3	154,2	130	22	4
14	2	43,9	146	102	26	10
14	2	54,3	154,8	107	34	10
14	2	44,7	155	143	41	12

14	2	47,5	158,2	112	23	12
14	2	49	154	127	34	4
14	2	50,4	157,1	150	37	20
14	2	46,7	151,1	132	38	21
14	2	57,5	162,1	160	11	1
14	2	58,6	166,7	131	28	3
14	2	55	160,2	124	16	6
14	2	52,4	154,5	73	17	12
14	2	51,1	155,8	128	25	12
14	2	48	158,6	153,5	37	26
14	2	47,8	162,8	118	23	15
14	2	42,8	157,2	87	29	7
14	2	57,9	159,5	116	36	7
14	2	42,7	158,3	90	15	13
14	2	51	167,3	139	25	18
14	2	52,2	163,5	126,5	16	16
14	2	55,6	168,3	108	27	10
14	2	51,4	157,1	122	26	26
14	2	51,4	160	130	28	15
14	2	39,8	147,6	102	29	32
14	2	45,3	164,1	53,2	23	4
14	2	40,9	155,7	111	18	14
14	2	51,2	156,1	133	36	16
14	2	47,8	154,3	105	26	19
14	2	54,1	166,1	144	42	10
14	2	45,7	161,4	110	27	10
14	2	48,7	152,1	98	22	11
14	2	48,8	159,6	147	26	29
14	2	49,4	158,3	170	30	25
14	2	50,9	155,1	65	32	8
14	2	50,3	163,2	107	24	9
14	2	62,9	164,2	142	30	5
14	2	99,5	159,1	74	17	7
14	2	71,6	173,5	111	8	3
14	2	56,1	153,5	102	34	3
14	2	58,6	156,2	147	22	13
14	2	67,7	158,1	136	25	10
14	2	67,5	165	107	11	12
14	2	66	154,1	112	25	3
14	2	57,9	151	92	30	16
14	2	59	153,9	110	26	3
14	2	70,4	165,2	159	31	21
14	2	64,5	160,7	140	45	18
14	2	63,6	160,2	97	24	9
14	2	58,1	153,2	73	27	4
14	2	69,1	162,5	121	32	7
14	2	48,5	154,3	113	27	15
14	2	51,7	153,3	127	18	13
14	2	52,6	155,5	97	24	10
14	2	42,4	158,3	122	29	7

14	2	42,6	156	92	20	9
14	2	42,2	155,8	124	35	6
14	2	58,2	158,4	115	32	9
14	2	40,7	162,3	90	34	10
14	2	41,6	156	160	25	15
14	2	47,1	155	149	28	40
14	2	68	171	145	23	25
14	2	45	146	110	40	21
14	2	50,5	156	134	30	2
14	2	65,8	164,8	103	10	8
15	1	69	179,2	139	17	15
15	1	71	182,9	170	33	16
15	1	54,6	171,2	144	19	8
15	1	59,6	174	229	27	35
15	1	46,5	169	188	34	12
15	1	62,1	183	180	32	13
15	1	64,3	178,1	145	21	7
15	1	62,6	181,3	160	38	36
15	1	62,7	180,8	185	29	23
15	1	148	175,5	141	16	23
15	1	97,5	177,6	154	23	7
15	1	74,2	163	160	30	13
15	1	90,4	178,5	174	33	19
15	1	54,8	184,5	147	8	9
15	1	47,3	159,2	180	26	25
15	1	56,6	168,1	194	29	14
15	1	69,1	183,7	175	28	4
15	1	63,3	180,2	192	14	21
15	1	54,1	164,2	195	25	13
15	1	64	180,1	183	23	17
15	1	45,3	160,3	158	31	15
15	1	55,2	172,5	175	27	19
15	1	52,7	173,3	140	26	18
15	1	51	164,3	184	23	15
15	1	58,7	184	137	1	5
15	1	59,1	172	164	27	22
15	1	59,1	180,3	146	8	10
15	1	59	167,3	212	23	10
15	1	40	151,2	182	22	20
15	1	50,1	169,1	183	28	23
15	1	61,3	166,5	200	41	33
15	1	68,3	178,9	220	34	17
15	1	60,5	184,6	200	20	14
15	1	49	168,2	212	38	10
15	1	64,1	178	189	25	8
15	1	59,2	170,9	203	25	25
15	1	78,4	185,2	203	18	5
15	1	61	165,5	170	26	23
15	1	102,2	174	167	19	1
15	1	70,3	168,5	230	31	28

15	1	75,2	170,1	191	34	41
15	1	81	178	188	26	15
15	1	94,6	184,1	138	22	9
15	1	62,5	169,2	139	22	18
15	1	60,3	172,1	135	31	1
15	1	59,6	167,5	164	39	15
15	1	60,8	170,4	210	29	15
15	1	59,7	178,2	180	24	21
15	1	70,6	184	218	27	28
15	1	47,3	164,4	166	32	25
15	1	77,2	181,2	142	20	12
15	1	49,4	170,1	155	37	17
15	1	70,2	192,8	148	36	8
15	1	50,1	174,1	183	20	6
15	1	47,6	166,8	151	22	7
15	1	57,4	172,8	151	23	10
15	1	45	155,6	192	21	22
15	1	70,4	178,1	178	19	8
15	1	52	178	145	28	15
15	1	58,6	168,5	181	21	17
15	1	58,2	165	194	16	19
15	1	52,7	168	160	26	23
15	1	45	165,6	149	26	17
15	1	55	175,8	160	24	5
15	1	64	170,3	156	21	10
15	1	55,4	168	182	26	8
15	1	55,8	169,4	178	24	17
15	1	60,7	163,2	186	35	23
15	1	58,1	165,4	165	25	15
15	1	51,3	164,5	173	33	20
15	1	62,2	175,5	210	30	30
15	1	60,2	169,3	208	19	14
15	1	57,1	167,6	150	13	11
15	1	59,2	170,5	239	35	20
15	1	43,6	168,1	139	28	12
15	1	67,3	190,6	158	8	15
15	1	68,1	176	147	31	17
15	1	69,7	188,4	180	22	17
15	1	64,4	183,9	133	28	10
15	1	63,2	177,8	205	27	15
15	1	63,5	174,1	162	28	15
15	1	70,8	181,2	145	23	12
15	1	51,8	169,3	183	36	39
15	1	50,6	166	137	23	11
15	1	68,2	178,8	193	27	4
15	1	71,1	175	165	23	12
15	1	48,7	170,1	202	10	12
15	1	66	175	174	32	30
15	1	59,4	175,2	155	30	20
15	1	58,2	176,7	194	36	10

15	1	43,3	164	170	26	1
15	1	55,2	165,3	159	16	6
15	1	60,5	168,3	162	27	20
15	1	60	170,1	206	33	50
15	1	64,7	182,5	182	31	22
15	1	52,7	162	221	29	50
15	1	71,1	190	178	40	16
15	1	67,2	168	184	30	15
15	1	61,1	165	205	27	15
15	1	55,9	173	191	23	10
15	1	55,6	176,2	130	26	20
15	1	52,8	162	198	34	20
15	1	68,2	184,2	198	25	32
15	1	64,1	166,8	230	36	22
15	1	66,1	183,2	177	25	5
15	1	50,8	160,6	125	23	10
15	1	56,8	168	186	26	8
15	1	66	175,5	167	17	24
15	1	110	171,5	145	29	1
15	1	102,3	183,2	148	40	3
15	1	90,5	169,3	156	21	2
15	1	78,1	176,5	161	33	16
15	1	70,5	171,5	130	13	9
15	1	82,2	179	154	12	3
15	1	73,3	168,8	170	30	20
15	1	80,2	176,9	150	20	3
15	1	70,9	173,3	206	22	6
15	1	70,2	164	173	40	22
15	1	76	174,1	230	30	21
15	1	72,4	169,5	163	32	1
15	1	89	177,5	169	19	1
15	1	60,5	167,3	152	27	28
15	1	57	176	207	30	1
15	1	60,8	170,1	196	27	19
15	1	62,9	171	194	27	12
15	1	66,9	168,6	103	30	5
15	1	68,5	172,4	159	24	8
15	1	64,5	173	186	27	19
15	1	53,5	169,5	137	32	15
15	1	58,7	167,8	179	23	30
15	1	63,2	178,2	182	29	15
15	1	66,5	179	181	21	20
15	1	59	174,5	120	32	20
15	1	47,8	168	164	10	10
15	1	68,8	176,8	170	19	14
15	1	63,8	180,7	209	1	18
15	1	30,3	141,8	130	22	12
15	1	59,5	173,2	189	28	9
15	1	55,6	175,9	189	28	23
15	1	68,5	175	216	27	10

15	1	60,1	167	153	25	38
15	1	53,2	157,3	183	30	10
15	1	46,3	158	184	33	31
15	1	37,7	150	186	26	30
15	1	67,9	186,2	169	1	10
15	1	48,2	161,9	120	32	9
15	1	55,6	173,2	165	34	17
15	1	63,4	174	184	12	10
15	1	66	168,2	154	37	6
15	1	53,7	168	216	32	20
15	1	66,2	174,3	190	15	18
15	1	58,3	178,5	227	37	23
15	1	68,2	179,3	155	23	10
15	1	52,4	160,8	159	21	15
15	1	67,2	170	121	27	30
15	1	50,4	170,5	145	23	8
15	1	56,2	168,2	175	28	18
15	1	75,5	180	158	29	10
15	1	55,7	174,4	188	13	4
15	1	58,2	163,2	161	32	20
15	1	42,1	166	178	27	12
15	1	60,3	169	167	28	5
15	1	71,7	181,5	160	27	5
15	1	53,2	170,8	163	29	9
15	1	52,1	173,5	131	16	15
15	1	52,2	170	143	38	5
15	1	57,5	173,5	200	25	20
15	1	63,8	171,3	192	35	17
15	1	55,7	172,1	190	23	10
15	1	56,8	178,1	157	26	17
15	1	76,4	183	166	26	14
15	1	59,3	168	194	36	20
15	1	67	185,3	195	31	28
15	1	57,2	179,1	167	29	18
15	1	66	170	190	31	41
15	1	58,5	173,2	217	31	1
15	1	50,7	163,8	173	25	30
15	1	61,4	170,1	120	19	4
15	1	64,1	170,2	194	33	8
15	1	62,4	175	193	26	21
15	1	50	161,1	158	19	19
15	1	55,1	171,7	226	30	16
15	1	65,3	181,7	181	26	25
15	1	67,2	187	170	28	19
15	1	53	170,5	189	23	23
15	1	57,7	167,5	176	21	23
15	1	58,4	182,5	199	35	18
15	1	79,1	191,3	217	17	34
15	1	95,1	179,1	181	38	15
15	1	96,4	172,8	188	32	10

15	1	87,2	173	178	24	16
15	1	87,9	160,4	130	28	5
15	1	81,4	179,2	150	27	1
15	1	76,5	179	161	26	3
15	1	73	173,4	150	27	13
15	1	85,3	172,4	144	18	7
15	1	63,5	161	165	26	8
15	1	73,9	173	180	32	2
15	1	46,2	155,1	151	25	12
15	1	50,8	160	181	30	40
15	1	61,2	166	176	34	27
15	1	56,5	175,1	170	27	7
15	1	53,8	177,5	158	31	30
15	1	45,3	165,2	146	19	4
15	1	63	173,7	199	31	18
15	1	66,3	169,2	170	39	24
15	1	50,3	155,6	181	25	40
15	1	58	173,2	177	20	10
15	1	55,5	163,7	213	41	30
15	1	56	167,6	189	41	21
15	1	56,3	173,5	158	13	11
15	1	61,3	165	213	27	16
15	1	46,8	161,2	193	31	23
15	1	56,5	173,4	138	29	11
15	1	43,8	161,5	156	20	17
15	1	51,9	165,3	125	23	12
15	1	53,2	174,2	158	27	18
15	1	54,9	156,8	196	28	19
15	1	41,1	164,5	152	30	18
15	1	60,1	173,5	162	23	10
15	1	58,5	173,4	110	21	1
15	1	54,2	170,8	200	25	20
15	1	70	177	174	29	8
15	1	58	175,5	138	43	25
15	1	55,1	176,8	115	23	2
15	1	57	177,6	173	35	13
15	1	61,6	171	232	24	5
15	1	51,1	161,7	195	37	27
15	1	53,8	160	126	35	15
15	1	48,5	159,7	166	31	5
15	1	60,1	181,6	180	14	17
15	1	51,4	161	134	25	28
15	1	60,3	176	227	26	21
15	1	96	170	153	29	33
15	1	62,7	164,7	165	29	9
15	1	81,6	177,3	170	33	29
15	1	97	183,4	132	33	8
15	1	64,2	169,5	191	44	18
15	1	55,2	154,2	138	26	1
15	1	48,1	164,9	130	24	3

15	1	64	170,4	197	33	15
15	2	58,5	163,1	103	25	8
15	2	58,1	165	122	21	6
15	2	52,3	162,4	146	29	20
15	2	64	157,3	158	33	44
15	2	68,5	170,2	108	25	5
15	2	58,2	167,4	100	12	1
15	2	63,2	163	109	34	1
15	2	49,9	161,5	133	24	15
15	2	54,2	168	120	34	10
15	2	54	152,5	150	34	19
15	2	55,1	164	162	25	16
15	2	55,3	156,3	99	14	2
15	2	49,8	160,1	112	24	8
15	2	48,4	164,4	131	38	1
15	2	47	163,1	155	32	4
15	2	53,4	174,2	145	29	21
15	2	56,5	159	119	26	12
15	2	52,1	161,2	122	40	11
15	2	56,8	170,5	125	25	12
15	2	64,5	163,5	106	22	2
15	2	54,8	171	137	31	5
15	2	53,5	158,5	120	29	9
15	2	47,1	163,6	116	44	20
15	2	48,4	163,6	116	28	3
15	2	55	164	142	28	8
15	2	50,3	157,5	143	17	2
15	2	48,3	165,6	94	31	10
15	2	48,9	166,6	98	22	7
15	2	53,8	157	130	21	22
15	2	56,7	153	122	28	7
15	2	58,2	153,8	112	26	6
15	2	65	160,3	90	11	5
15	2	80,2	169,2	117	6	7
15	2	77,1	164,5	98	29	1
15	2	71,1	163,1	104	28	1
15	2	60,7	156,2	80	27	7
15	2	53	146	90	23	21
15	2	47,5	160,1	131	20	11
15	2	53	162	112	18	11
15	2	64,9	173	104	26	20
15	2	46,2	161,2	123	21	6
15	2	47,8	164	87	34	10
15	2	40,8	154	150	34	14
15	2	41,2	160,4	103	16	4
15	2	49,3	163,5	123	25	12
15	2	52,8	153,3	154	31	23
15	2	55,1	173,9	140	28	15
15	2	47,3	161,5	110	29	4
15	2	45,7	163,9	114	13	5

15	2	42,1	151,7	132	37	9
15	2	49,1	165	163	30	35
15	2	44,9	164,8	100	26	10
15	2	44,6	162,1	128	27	32
15	2	41,1	161,1	83	23	3
15	2	51,1	159,3	120	25	13
15	2	50,2	168,9	112	23	12
15	2	55	158,4	107	34	19
15	2	51,4	160,2	101	13	2
15	2	57,5	168,9	111	23	12
15	2	65,1	174,3	85	24	16
15	2	44	164,2	95	13	1
15	2	49,5	167,7	100	4	4
15	2	52,1	155,8	109	25	3
15	2	50,6	159,9	143	36	30
15	2	46,2	163,2	110	32	6
15	2	46,3	157,4	107	35	14
15	2	60,6	166,1	91	13	5
15	2	69,1	171,1	122	32	18
15	2	50,4	174,1	147	26	4
15	2	55,8	158,5	118	27	10
15	2	48,5	160,2	150	29	25
15	2	55,3	156,4	96	27	20
15	2	52,5	164	160	41	22
15	2	63	166,1	138	13	12
15	2	60	167,3	94	30	12
15	2	60,2	176,2	130	25	8
15	2	54	154,5	103	28	12
15	2	55,1	171,5	129	23	8
15	2	51,2	158,9	89	21	13
15	2	46,2	154,5	82	22	11
15	2	44,7	155,4	68	22	12
15	2	48,5	147,2	130	34	32
15	2	53,3	163,5	125	31	19
15	2	62,9	166,9	142	27	11
15	2	49,2	158	123	21	7
15	2	61,9	160	115	43	19
15	2	51,1	156,7	110	32	24
15	2	58,7	162,5	112	25	11
15	2	58,8	173,4	125	39	10
15	2	40,1	163,1	100	2	1
15	2	42,2	163,4	135	34	7
15	2	47,1	150	106	35	10
15	2	47,6	156,4	80	27	10
15	2	54,5	155,7	106	34	8
15	2	55,8	165,8	130	25	17
15	2	46,9	169,3	103	19	10
15	2	67,1	169,7	94	19	4
15	2	60,8	166,9	189	38	23
15	2	61,2	186	105	28	12

15	2	56,2	162,5	136	31	3
15	2	47,8	153,8	120	20	11
15	2	50,4	161,9	130	23	8
15	2	55,5	155,4	120	33	30
15	2	54,6	162	147	33	15
15	2	54,2	163,3	120	10	10
15	2	54,7	175,3	110	34	6
15	2	58,8	172,7	125	35	9
15	2	59,2	164,5	122	19	10
15	2	51,2	161,1	108	25	13
15	2	57,5	165,8	97	16	7
15	2	39,5	154,5	133	25	8
15	2	61,3	160,3	90	29	20
15	2	53,4	158,2	126	29	6
15	2	61,5	169	119	29	9
15	2	45,1	159,8	128	31	6
15	2	49,3	161,2	145	31	19
15	2	56,5	172	140	37	12
15	2	48,1	155	122	29	8
15	2	67,2	169,9	100	27	3
15	2	49	155	121	23	5
15	2	71,5	173,2	157	35	10
15	2	65,9	146,1	82	28	10
15	2	74,3	156,2	105	25	1
15	2	82,4	166	148	30	19
15	2	64,2	163,2	136	30	11
15	2	62,9	158,8	98	8	7
15	2	61,1	156,3	103	28	15
15	2	72,8	166,2	83	25	9
15	2	65,6	158	154	38	24
15	2	71	163,5	99	28	14
15	2	74,3	170,8	118	12	1
15	2	66,3	156	117	32,5	8
15	2	48,9	172,3	149	26	31
15	2	57,9	167,9	128	18,5	3
15	2	42,3	147,5	112	23	5
15	2	52,5	161,3	142,5	24	10
15	2	47,1	152,8	146	34	29
15	2	50	165	110	24	1
15	2	52,6	170,8	133	36	4
15	2	44,2	166,1	140	25	14
15	2	53	163,9	93	20	10
15	2	44,5	163,4	98	18	11
15	2	64,5	166,8	103	28	7
15	2	46,9	147,5	100	42	13
15	2	58,7	165,9	126	31	19
15	2	59	160,6	105	31	13
15	2	58,9	162	118	21	15
15	2	43,4	154,3	95	18	8
15	2	51,1	159	114	28	7

15	2	46,4	151,5	141	44	30
15	2	42,5	160,5	140	23	17
15	2	49,8	155,1	105	23	12
15	2	50,4	159,2	118	28	9
15	2	47,6	166,2	118	30	1
15	2	50,3	152,7	76	22	12
15	2	62,2	167	115	32	5
15	2	61,5	163,8	124	25	20
15	2	54,5	165	131,5	25	21
15	2	51,4	159	110	14	8
15	2	50,2	169,5	131	29	17
15	2	45,3	160,8	129	37	11
15	2	48,6	167,2	99	23	8
15	2	59,2	165,2	147	27	22
15	2	42	159,9	110	26	5
15	2	50,1	166,7	120	25	8
15	2	37,3	153,2	155	44	4
15	2	54,2	168	112	22	30
15	2	54	168	110	23	3
15	2	56,7	164,5	125	29	7
15	2	65,1	166,1	101	27	1
15	2	52	181,6	120	10	2
15	2	57,6	173,4	145,5	21	10
15	2	46,2	164,1	91	34	7
15	2	38,3	147,8	70	29	1
15	2	48,8	162,9	130	36	11
15	2	52,5	163	111	27	22
15	2	47,9	166	125	25	19
15	2	60,2	170,9	160	29	25
15	2	55,1	162,7	123	28	12
15	2	40,8	155,9	158	27	13
15	2	52,9	170	122	9	2
15	2	56,7	159,4	115	24	5
15	2	53,8	165	132	7	16
15	2	45,3	164	110	41	8
15	2	54,9	154,7	121	40	20
15	2	51,2	160,1	129	35	6
15	2	53,1	159,5	145	31	9
15	2	60,2	160	133	39	18
15	2	55,8	162,2	128	34	20
15	2	62,7	165,7	103	15	4
15	2	48,1	155	184	40	30
15	2	74,2	175	111	32	1
15	2	60,8	160,5	131	24	8
15	2	54,6	168,1	121	28	8
15	2	61,4	166	103	35	2
15	2	49,9	153	100	13	17
15	2	60,2	161,3	112	23	5
15	2	53,5	167,1	140	26	25
15	2	70	175	78	34	6

15	2	54,3	174,8	141	39	20
15	2	51,8	158,9	120	34	9
15	2	53,3	166,1	138	20	12
15	2	55,4	162,5	133	31	9
15	2	66,4	169,3	88	24	15
15	2	48,1	152,4	125	27	17
15	2	55,1	157,8	53	35	11
15	2	48,3	155,5	152	31	19
15	2	54,2	172	118	25	7
15	2	55,1	165	120	27	2
15	2	43,1	162,1	121	25	8
15	2	60,3	166,8	89	21	15
15	2	63,8	171,3	86	10	24
15	2	49,4	159,1	124	18	6
15	2	46,3	162,8	130	29	5
15	2	55,6	169	80	20	6
15	2	47,4	163,9	115	26	1
15	2	58,2	169	102	38	10
15	2	46,1	159,2	119	18	7
15	2	97,8	162	115	30	9
15	2	79,2	159,7	50	22	1
15	2	72,2	152,9	134	32	10
15	2	82,9	168,8	88	34	2
15	2	71,3	166,9	142	35	30
15	2	75,2	170,3	94	15	1
15	2	71,3	157,8	98	20	1
15	2	58,5	142,6	130	36	12
15	2	69,9	162,5	118	40	11
15	2	69,7	164	125	16	2
15	2	77	165,5	89	24	2
15	2	63,5	155,3	140	32	26
15	2	50	156,5	132	22	37
15	2	51,8	164	140	41	4
15	2	55,1	170,1	145	22	8
15	2	42	160	141	37	14
15	2	47,87	162,1	118	38	13
15	2	52,3	161,7	125	25	25
15	2	42,3	142	147	27	15
15	2	56,6	163	143	20	10
15	2	50	153,7	100	32	13
15	2	60,9	162	141	32	9
15	2	47,7	160,1	127	30	22
15	2	49,9	159,2	99	24	15
15	2	52,4	153,5	94	29	10
15	2	46,2	156,1	146	28	9
15	2	43,8	165,1	123	23	8
15	2	48,2	156,2	140	34	21
15	2	55,2	162	109	34	10
15	2	54,9	155,8	105	31	9
15	2	55,1	170,5	121	17	8

15	2	54,2	159,9	89	18	4
15	2	61,6	164	170	33	20
15	2	43,8	152,4	110	42	19
15	2	55,3	159	119	27	8
15	2	38,1	157,7	136	31	5
15	2	43,7	160,3	143	37	12
15	2	51,7	158,1	127	35	20
15	2	54,9	156,4	127	37	15
15	2	52,5	163	127	27	44
15	2	58,7	160,5	108	23	22
15	2	57,1	162,9	99	33	9
15	2	49,4	154,5	89	21	1
15	2	47,7	167,4	121	24	11
15	2	64,1	156,1	98	21	4
15	2	68,3	167	100	37	19
15	2	69,3	166,5	135	24	10
15	2	69,5	160,8	121	20	10
15	2	55,2	165,2	118	21	4
15	2	43,6	167,3	140	31	1
15	2	56,1	160,7	120	27	10
15	2	43,2	156,8	117	32	5
15	2	60	163,5	106	22	5
15	2	49,5	161	140	29	12
15	2	50,8	150	112	31	4
15	2	40,9	159,2	113	11	11
15	2	115,6	170,3	97	34	12
15	2	69,2	155	93	27	12
16	1	86,9	190,2	208	30	26
16	1	77,7	181,8	172	28	25
16	1	60,5	168,2	175	32	18
16	1	57,8	165,5	185	32	29
16	1	53,3	177,2	162	27	3
16	1	73	188,5	198	29	18
16	1	62,1	184	190	36	11
16	1	55,3	174,5	113	23	11
16	1	62,1	164,5	190	30	35
16	1	83,2	181	173	22	12
16	1	77,2	170,9	195	29	19
16	1	46,7	170,1	189	16	7
16	1	70,9	174,5	210	29	5
16	1	59,3	166	135	18	23
16	1	54	177,2	163	25	17
16	1	61,9	164,5	194	27	2
16	1	55,7	174,2	185	31	14
16	1	63,8	177,2	212	29	31
16	1	63,2	180,5	162	29	18
16	1	54,4	166	188	21	47
16	1	51,1	168	208	33	20
16	1	53,4	174,1	192	22	30
16	1	52	172	241	33	23

16	1	59,5	173,5	185	28	19
16	1	65,4	180,1	160	22	7
16	1	49,1	165,5	205	27	30
16	1	76,3	181	169	29	30
16	1	70,8	191	205	28	6
16	1	71,8	174,2	198	30	16
16	1	92,1	195	172	21	8
16	1	64,6	168	170	29	10
16	1	56,7	172,1	189	21	17
16	1	64,3	176,5	170	27	25
16	1	49,8	171	140	26	20
16	1	79,4	179,8	213	39	27
16	1	77,2	164,9	181	17	24
16	1	79,8	175,2	162	19	12
16	1	82,5	175,4	159	35	26
16	1	79,6	174,1	170	29	20
16	1	64,8	176,5	185	32	30
16	1	61,5	173,4	209	26	32
16	1	56,7	171,5	206	34	25
16	1	60,4	175,4	177	27	17
16	1	63,7	179,2	169	5	6
16	1	56,5	170	182	24	33
16	1	48,2	163,2	159	5	8
16	1	66,4	176,1	185	26	14
16	1	48,3	168	166	16	12
16	1	62,8	166,8	153	16	15
16	1	52,8	178,1	194	34	25
16	1	50,4	160,1	198	31	55
16	1	68,3	173,5	183	41	6
16	1	67,6	181,1	168	25	15
16	1	63	184,9	180	30	29
16	1	60,5	167,2	182	3	12
16	1	71,8	190,2	165	29	10
16	1	55,1	171,7	183	31	17
16	1	63,4	177,2	185	23	19
16	1	67,1	183,4	208	33	25
16	1	65,5	174	168	30	9
16	1	45,3	172,5	173	24	5
16	1	62,1	174,6	212	33	25
16	1	64,1	176,5	148	15	5
16	1	67,6	175	135	23	10
16	1	73,8	182,5	163	26	21
16	1	76,6	180	250	41	37
16	1	63	165,7	162	36	20
16	1	64,5	178	219	44	45
16	1	60,7	173,2	217	26	24
16	1	74,3	182	185	26	28
16	1	39,5	146	114,6	17	1
16	1	71,8	190,7	180	2	3
16	1	68,3	177	162	24	18

16	1	59,5	169	115	18	2
16	1	61,2	169	181	21	20
16	1	69,8	178,2	171	26	9
16	1	53	173,5	187	26	16
16	1	71,5	180	209	25	20
16	1	70,2	176,5	211	33	45
16	1	60,9	181	188	25	30
16	1	62,5	183,2	129	23	14
16	1	52,1	179,2	174	23	10
16	1	61,8	175,1	131	28	16
16	1	58,2	173,5	148	26	8
16	1	64,6	176,5	226	46	17
16	1	85,4	170,3	111	23	9
16	1	112,1	177,2	112	16	5
16	1	92	176,2	200	24	5
16	1	98,9	176,5	150	32	10
16	1	104,3	177,3	104	23	7
16	1	119,5	179,8	172	26	3
16	1	103,5	170,4	146	35	4
16	1	72,9	171,9	259	52	50
16	1	68,3	162,9	188	35	30
16	1	86,1	180,2	145	38	20
16	1	74,5	170,3	162	28	17
16	1	78,5	178,2	178	41	25
16	1	89,2	176,7	145	23	10
16	1	66,4	171,5	172	25	18
16	1	65,5	175,5	172	25	9
16	1	66,6	175,1	165	35	3
16	1	59,6	182	194	24	14
16	1	50	164,2	168	20	7
16	1	58,7	181,6	172	28	18
16	1	59,1	162	187	35	35
16	1	59,6	180	136	25	8
16	1	57,3	172	138	27	12
16	1	65,3	185,1	109	28	10
16	1	53,8	172,8	144	31	3
16	1	52,1	167,3	153	28	25
16	1	57,8	170,5	170	21	12
16	1	60,8	178,2	185	29	28
16	1	58,2	177,5	152	24	17
16	1	59,3	176,8	214	32	27
16	1	69,7	170	179	33	21
16	1	50,8	159,5	144	42	19
16	1	53,6	165,6	196	23	16
16	1	65,6	174	224	27	12
16	1	59,9	171,2	197	23	24
16	1	70,1	181	194	30	19
16	1	59,8	169,7	243	31	20
16	1	70,3	179,1	210	25	37
16	1	68	172,8	153	33	18

16	1	49,6	171,2	190	27	20
16	1	58,3	166,5	187	18	9
16	1	70,2	173,4	190	31	30
16	1	62,1	187,5	183	14	20
16	1	54,6	161,3	161	23	8
16	1	36,8	147,5	170	21	15
16	1	48,3	162	208	23	22
16	1	55,4	172	179	25	16
16	1	58,2	179,2	196	43	35
16	1	57,9	167,6	125	23	15
16	1	60,7	173,2	187	28	18
16	1	68,2	176	198	34	18
16	1	98	175	110	11	5
16	1	93,1	171,1	128	21	5
16	1	99,1	178	114	26	4
16	1	96,4	167,1	110,5	2	2
16	1	68,7	164	210	35	57
16	1	69,4	165,2	135	20	6
16	1	97,2	183	169	23	6
16	1	82,5	178,1	205	31	38
16	1	97,3	185,9	160	30	5
16	1	78,5	179	182	23	7
16	1	58	177,4	180	35	32
16	1	65	175	206	24	20
16	1	60	164,3	130	20	10
16	1	57,6	174,1	186	25	19
16	1	73,6	182,1	178	26	20
16	1	54,3	170	183	28	20
16	1	64	164	120	25	15
16	1	51,1	161,7	195	37	27
16	1	69,7	181,7	222	19	50
16	1	64,5	170	176	36	20
16	1	63,1	180,2	181	21	12
16	1	74,5	176,3	166	29	12
16	1	80,4	183,4	172	25	5
16	1	56,6	168,2	125	24	19
16	1	60,8	175,5	202	28	27
16	1	57,7	166,7	184	19	12
16	1	54,2	170,9	173	34	17
16	1	77,3	180,7	176	16	9
16	1	74,2	170,3	147	17	13
16	1	59,6	166,2	170	26	28
16	1	53,9	166,3	190	40	49
16	1	90	173,3	162	31	16
16	2	48,2	161,9	137	19	20
16	2	51,6	173	110	15	15
16	2	56,3	160,9	121	13	18
16	2	58	165,3	111	15	5
16	2	53,3	161,5	96	24	40
16	2	58	165,6	105	18	2

16	2	47,6	152,2	127	18	15
16	2	45,9	162,2	100	12	13
16	2	60,1	166,1	109	23	10
16	2	51,9	161,4	106	39	1
16	2	47	161	90	15	20
16	2	46,3	160,3	115	26	12
16	2	65,7	168,1	125	26	9
16	2	53,1	164,9	94	24	29
16	2	57,4	173,5	151	9	5
16	2	53,3	162	117	28	12
16	2	42,3	157,1	140	23	25
16	2	57,6	163,2	114	17	14
16	2	47	166,5	85	26	12
16	2	48,7	162,5	138	41	10
16	2	39,9	157	90	24	10
16	2	53,8	163	147	30	10
16	2	62,2	167	120	29	17
16	2	48,7	157,1	136	24	14
16	2	57,3	169,1	132	33	15
16	2	55,5	169,2	109	21	7
16	2	87,3	161	95	30	10
16	2	80	163,3	110	27	10
16	2	61	156,5	90	29	8
16	2	71,3	163,2	106	25	6
16	2	70,8	161,5	115	26	8
16	2	67,6	164,5	130	30	2
16	2	62	158,1	138	32	20
16	2	42,4	160,1	126	6	14
16	2	47	168,5	100	28	10
16	2	40,8	165,9	106	22	4
16	2	64,1	170,8	98	5	3
16	2	44	162	95	22	6
16	2	59,5	169,3	97	23	15
16	2	53,7	161,3	147	23	13
16	2	63,2	180,1	119	23	6
16	2	47,3	149,5	112	20	17
16	2	43,1	156,6	121	30	22
16	2	39,8	154	80	35	2
16	2	43	160,1	133	28	7
16	2	58,6	169,2	125	23	12
16	2	43,4	155	93	23	4
16	2	59,4	174	150	22	17
16	2	58,2	165	119	40	8
16	2	55,4	168,7	132	15	2
16	2	51,2	161,2	130	28	21
16	2	38,6	167,8	90	26	13
16	2	52,3	171,8	131	21	9
16	2	56	156	135	32	13
16	2	54,9	169,2	125	26	9
16	2	63,7	172,5	128	38	15

16	2	62,3	161,3	100	25	10
16	2	51,8	167,8	137	31	15
16	2	53,2	166,1	104	17	10
16	2	56,4	157,3	130	35	26
16	2	52,2	159,2	128	21	20
16	2	49,2	166,9	121	27	8
16	2	54,6	166	136	20	5
16	2	70,6	170,7	100	26	1
16	2	63,5	164,4	153	30	8
16	2	59,7	170,3	141	32	11
16	2	49,1	168,6	164	30	10
16	2	58,9	156,5	141	18	14
16	2	53,7	155,9	115	30	22
16	2	54,4	168,2	108	22	8
16	2	48	157,6	145	37	1
16	2	63,8	173,4	159	29	15
16	2	61,8	175,7	144	21	10
16	2	46,6	162,3	122	40	12
16	2	71,3	178	101	30	10
16	2	52,1	164,1	110	25	4
16	2	48,7	159,2	125	29	12
16	2	54,7	164,3	114	33	1
16	2	47,8	150,3	125	26	8
16	2	48,7	165,3	138	27	8
16	2	61,7	161,3	132	30	11
16	2	55,1	162	140	12	1
16	2	42,3	165	145	27	11
16	2	51,6	169,2	131	34	13
16	2	49,2	155,2	120	31	20
16	2	45,7	151,8	104	26	13
16	2	47,7	155,2	140	37	20
16	2	52	154,5	137	40	14
16	2	57,2	163,3	112	29	7
16	2	49,5	167,6	104	29	1
16	2	97,3	165,8	80	25	1
16	2	68,2	165,1	112	20	3
16	2	74,3	168,3	110	32	8
16	2	68,3	159	105	23	17
16	2	58,1	152,8	98	28	17
16	2	62,5	155,8	80	24	10
16	2	67,5	164,2	101	25	3
16	2	72,6	160,3	83	33	25
16	2	67,5	161,5	105	23	10
16	2	69	158	128	32	25
16	2	68,3	153,2	89	31	17
16	2	56,5	170,8	93	16	9
16	2	51,8	162	123	27	3
16	2	46,5	160	109	28	7
16	2	50,9	157,5	140	29	10
16	2	47	167,2	135	29	17

16	2	52,1	166,2	130	12	5
16	2	55,1	171,2	110	25	9
16	2	52,2	161	120	22	2
16	2	54,4	161	123	15	9
16	2	46,4	156	80	33	1
16	2	40,4	160,2	112	24	6
16	2	48,7	156,7	152	20	40
16	2	56,4	161,5	145	38	22
16	2	43,8	151,2	110	42	15
16	2	65,1	165,9	165	36	6
16	2	51,6	164,6	115	32	25
16	2	57	160	118	40	12
16	2	43,5	166,1	154	23	14
16	2	48,2	149,8	105	20	12
16	2	57,1	170	96	26	5
16	2	55,9	153	102	7	3
16	2	64	162	112	18	12
16	2	56,4	155,3	142	29	3
16	2	56,9	167,3	138	25	9
16	2	52,8	173,5	108	21	3
16	2	51,3	156	120	22	27
16	2	58,5	165	118	32	10
16	2	58,9	160	85	20	7
16	2	47,7	160,6	150	22	29
16	2	64,1	167,2	146	30	20
16	2	56,3	164,8	149	36	11
16	2	51,8	157	137	32	25
16	2	47,1	162,7	142	35	12
16	2	47,1	151,1	135	47	10
16	2	42,7	168	109	26	2
16	2	69,2	170,6	151	36	10
16	2	51	153,7	90	26	10
16	2	48,2	154,6	108	31	19
16	2	44,5	155,1	147	29	20
16	2	53,5	156,1	166	31	20
16	2	52,5	160,7	138	22	21
16	2	52,1	156	112	22	11
16	2	47	167,6	105	19	7
16	2	61,2	159,2	88	29	2
16	2	49	159,2	105	36	8
16	2	101,2	160,5	93	20	12
16	2	84	156,5	106	23	2
16	2	78,6	158,3	84	9	5
16	2	68,3	156,1	108	23	5
16	2	69,3	156,2	142	29	8
16	2	71,6	164,6	128	20	1
16	2	63,7	160	145	27	21
16	2	66,8	162,3	105	30	1
16	2	58,4	150	118	11	23
16	2	66,3	164	150	20	15

16	2	52,2	173,4	117	22	7
16	2	51,9	171,3	119	27	12
16	2	61	175,9	92	26	8
16	2	45,5	164,6	105	18	8
16	2	52,5	161,4	92	24	4
16	2	47,3	140,5	122	20	4
16	2	48,5	160,5	120	35	12
16	2	50,1	157,9	111	19	14
16	2	55,5	168,3	127	22	7
16	2	59,2	159,3	104	28	8
16	2	43,1	146,5	113	15	1
16	2	51,4	159	93	26	2
16	2	63,4	167,2	120	32	15
16	2	50,1	158,7	116	39	26
16	2	52,6	156,2	92	35	14
16	2	48,5	158,6	100	36	21
16	2	47	149	105	21	6
16	2	57,8	166,3	100	15	4
16	2	71,1	169,8	105	25	2
16	2	62,1	163,7	110	35	31
16	2	48,7	151,3	112	26	30
16	2	49,4	155,4	106	34	31
16	2	50,4	161,1	119	32	10
16	2	58,1	163,6	129	27	11
16	2	48,8	158,5	135	25	15
16	2	59,6	174,5	123	22	5
16	2	48,7	162,7	100	19	1
16	2	38,8	151,6	146	6	13
16	2	58,5	170	158	29	7
16	2	54,8	160,4	128	34	10
16	2	64,7	168	126	24	1
16	2	53,8	162,1	83	25	19
16	2	48,4	163	102	17	3
16	2	94,6	184	87	11	1
16	2	55,2	149,2	109	42	25
16	2	62,6	157,3	127	32	3
16	2	65,1	162	125	29	17
16	2	59,7	155,4	144	30	8
16	2	50,3	154,9	100	31	12
16	2	59,1	174,3	146	17	25
16	2	39,5	171,9	108	16	6
16	2	49,5	152,3	108	21	9
17	1	140	181	102	26	5
17	1	63,3	181,2	205	28	18
17	1	71,2	185	230	34	3
17	1	58,7	180,3	165	11	16
17	1	73,5	184,2	239	29	19
17	1	57,1	179,2	186	23	12
17	1	73	179,3	221	27	33
17	1	52,8	165,5	186	26	24

17	1	56,1	171,1	129	23	8
17	1	70,1	173,3	145	20	17
17	1	58,3	165,2	165	22	15
17	1	69,3	186,2	223	40	28
17	1	72,1	181,6	195	21	10
17	1	58	175,1	213	39	50
17	1	71,1	180	209	35	24
17	1	65,3	184,6	217	39	20
17	1	72,4	185,1	190	25	15
17	1	70,2	178,1	210	36	35
17	1	63,2	170,1	215	18	27
17	1	104,8	175,5	183	24	30
17	1	81,2	173	185	17	20
17	1	87	182,5	135	20	5
17	1	88	178,5	145	22	10
17	1	92,6	190,3	201	32	15
17	1	73,3	170,6	180	17	11
17	1	84,5	175	230	28	67
17	1	87	182,1	148	23	12
17	1	65,6	170,1	182	28	25
17	1	64,2	173,1	184	20	22
17	1	65,2	181,1	165	19	11
17	1	51,1	170	201	16	31
17	1	51	165,5	193	25	20
17	1	56,5	171,2	175	31	25
17	1	52,5	171	184	30	17
17	1	70	170	179	36	9
17	1	68,4	177,5	245	30	13
17	1	60,7	171,5	192	35	10
17	1	51,7	173	204	25	30
17	1	61,5	180,8	190	20	9
17	1	60	173,6	223	37	25
17	1	55,9	179	160	20	24
17	1	58,6	177,1	207	25	16
17	1	74,8	186	170	14	9
17	1	72,5	176,1	160	35	31
17	1	72,1	183,3	239	38	24
17	1	75,2	178	208	25	15
17	1	65,6	166	143	11	16
17	1	75,3	178,4	156	26	18
17	1	74,5	174,3	120	23	18
17	1	57,8	181	192	25	14
17	1	71,1	183,5	161	20	13
17	1	70,3	191,5	119	17	9
17	1	76,7	194	160	22	15
17	1	66,1	181,1	176	25	30
17	1	60,3	180,6	220	1	8
17	1	58,6	176,2	186	29	7
17	1	77,7	183,9	225	29	30
17	1	73,1	184	222	23	10

17	1	70,2	182,2	180	11	21
17	1	55,4	170,2	150	17	12
17	1	52,5	167	208	30	28
17	1	54,1	167,6	174	27	11
17	1	68,5	188	196	31	2
17	1	61,2	165,9	182	32	30
17	1	62,9	182,6	124	24	10
17	1	74,5	176,1	194	22	44
17	1	58,5	173	174	30	29
17	1	61,2	170,8	187	23	25
17	1	119,8	183	142	34	8
17	1	81,5	176	215	29	21
17	1	75	173	193	28	17
17	1	84	180,1	224	47	38
17	1	80,6	173	230	42	20
17	1	84,5	183,2	156	15	15
17	1	90,9	181,5	162	23	4
17	1	77,1	173,6	130	23	22
17	1	73,3	163	165	19	2
17	1	67,9	177,2	156	21	14
17	1	61,9	183,5	182	22	19
17	1	56,5	167,2	193	42	30
17	1	52	166	151	33	7
17	1	59,7	163	157	27	35
17	1	59,7	172	195	26	18
17	1	69,5	172	178	26	20
17	1	75,4	182,4	220	33	8
17	1	73,8	182	188	22	27
17	1	69,5	181,9	213	31	38
17	1	57,3	176,2	167	23	12
17	1	55,7	170,6	197	20	3
17	1	71,1	178,9	176	33	13
17	1	80,2	184	190	35	15
17	1	61,5	180,4	185	20	9
17	1	64,2	172,5	158	22	2
17	1	48,5	170	138	25	7
17	1	58,9	171,8	164	24	14
17	1	64,1	167	190	30	54
17	1	55,5	174,2	175	33	8
17	1	68,3	178,5	254	38	52
17	1	89,3	191,5	231	26	30
17	1	55,1	181,1	163	21	11
17	1	58,9	171	160	43	21
17	1	51	158	143	33	11
17	1	59,2	163,8	208	15	33
17	1	60,5	164,5	232	24	30
17	1	59,1	171,8	213	36	11
17	1	56,4	173,5	190	20	42
17	1	97,1	171	200	38	15
17	1	92,8	171,8	185	36	20

17	1	94	172	190	26	2
17	1	95,8	175	152	28	27
17	1	92,7	178,4	158	24	25
17	1	90,5	177,6	217	33	51
17	1	75	159,2	182	25	18
17	1	74,3	187	209	34	15
17	1	49,3	167	245	31	20
17	1	61,1	181,4	192	29	18
17	1	51	158,3	154	30	41
17	1	61,2	176	205	32	20
17	1	46,5	165,5	193	30	11
17	1	55	163,3	187	37	13
17	1	59,9	167,2	160	32	5
17	1	65	174,2	185	29	39
17	1	51,9	162,5	185	19	20
17	1	65	171	191	36	16
17	1	65,5	184,2	198	25	19
17	1	88,1	177	140	16	5
17	1	84	187,3	165	18	5
17	1	63,5	172,2	215	29	10
17	1	61,6	169,4	199	31	9
17	1	46,3	165,1	121	13	2
17	2	59,1	163	159	39	22
17	2	67,5	172,7	114	30	20
17	2	58,4	173,6	112	31	1
17	2	61,8	159	105	33	10
17	2	54,1	164	110	29	9
17	2	60,4	158,1	136	28	30
17	2	52,4	160,1	130	29	18
17	2	48,3	160,2	102	25	8
17	2	48,5	167,2	130	23	8
17	2	47	150,5	140	35	10
17	2	53,5	163,5	140	37	7
17	2	58,5	154,3	120	36	17
17	2	77,1	162,1	65	21	6
17	2	68,8	162,3	132	21	30
17	2	71	163,9	130	39	30
17	2	48,9	158,1	109	22	22
17	2	52	159	121	35	21
17	2	48,2	160,9	122	29	12
17	2	60,1	167,9	91	30	15
17	2	48,4	163,5	130	31	11
17	2	43,3	159,8	131	34	36
17	2	43,8	162,6	106	21	8
17	2	54	159,6	103	25	8
17	2	68,5	168,3	142	30	10
17	2	47,4	162,9	152	29	6
17	2	48,3	164,2	130	28	12
17	2	58,7	162,2	123	23	5
17	2	48,3	150,5	110	19	6

17	2	51,8	165,4	126	31	12
17	2	63,9	170,2	89	13	14
17	2	54,5	165,2	125	36	3
17	2	59,1	165,7	132	38	11
17	2	49,9	164,2	120	23	6
17	2	48,9	164	108	34	8
17	2	60,9	165,5	110	30	15
17	2	48,2	161,1	142	27	18
17	2	51,2	160,5	120	34	7
17	2	49	172,4	117	25	20
17	2	61,9	178	130	17	19
17	2	47,9	157,4	100	35	6
17	2	43,8	160,2	120	28	12
17	2	55,6	165,5	160	36	30
17	2	51,6	161,1	96	16	8
17	2	57,3	163	132	38	12
17	2	53,2	165,2	80	33	8
17	2	69,9	169,2	140	25	1
17	2	48,5	153,2	123	35	18
17	2	58,2	159,5	131	30	20
17	2	58,6	162,7	166	20	14
17	2	49,2	154,3	103	30	1
17	2	41,2	160,2	125	17	21
17	2	58,3	173,2	109	15	11
17	2	54,7	178,1	161	27	8
17	2	44,1	168	144	21	8
17	2	69	174	80	32	30
17	2	68	168	90	35	15
17	2	58,9	154,1	148	33	8
17	2	50,3	169,1	131	28	12
17	2	54,6	172,1	120	34	4
17	2	55,3	167	110	25	8
17	2	58,1	155	112	29	5
17	2	52,8	162,1	134	30	20
17	2	54,8	171	118	23	10
17	2	58	159,2	143	31	3
17	2	93,9	161	120	17	11
17	2	68,4	165,2	101	31	13
17	2	69,9	162,5	108	23	8
17	2	70,3	163,5	100	28	16
17	2	65,3	157,1	104	31	8
17	2	46	152,5	102	28	28
17	2	47,6	160,2	121	35	2
17	2	60,8	174,3	217	28	22
17	2	58,9	164,1	120	13	10
17	2	61,9	163,5	152	15	8
17	2	58,7	170,6	145	32	8
17	2	65,3	166,7	99	10	2
17	2	55,1	170,2	125	29	8
17	2	64,5	163,2	96	32	14

17	2	64	166	90	22	2
17	2	61,8	166,3	118	25	10
17	2	56,7	158	104	26	20
17	2	68,4	170,4	132	29	12
17	2	46,5	162,5	142	32	18
17	2	49,3	156,5	95	30	12
17	2	36,1	152,1	96	19	7
17	2	50	164,2	128	34	28
17	2	46,9	161,3	113	29	11
17	2	55,6	165,4	123	30	18
17	2	48,1	150,3	92	37	10
17	2	67,5	165,3	110	31	5
17	2	61,9	166,8	118	25	27
17	2	46,5	157,3	109	29	16
17	2	55,1	167,3	123	31	13
17	2	58,2	162,1	108	25	8
17	2	64,3	164,5	138	36	22
17	2	68,1	168,9	100	10	3
17	2	43,2	161,2	128	8	3
17	2	68,6	175,5	91	15	4
17	2	44,9	152	96	27	5
17	2	59,4	163,2	118	28	18
17	2	52,8	165,1	110	23	22
17	2	53,1	162	145	26	12
17	2	50	160	140	35	20
17	2	63,2	164	129	32	3
17	2	51,4	156,1	110	36	15
17	2	47,1	156,3	77	34	20
17	2	59	160,1	103	25	1
17	2	51,5	161,3	113	23	20
17	2	56,2	162,1	120	29	6
17	2	55,4	164,5	112	30	1
17	2	80	158	98	32	1
17	2	62,2	153	92	28	18
17	2	63	156,3	107	37	10
17	2	67,6	164,6	119	27	6
17	2	57	145,3	87	43	10
17	2	62,2	153	92	31	30
17	2	71	155,8	85	2	1
17	2	46	155,9	90	20	2
17	2	49	158	82	19	12
17	2	55,3	170,5	119	27	10
17	2	53,5	156,6	120	25	16
17	2	60,9	160,9	143	29	12
17	2	60,5	162	110	17	2
17	2	56,5	151	121	29	5
17	2	53,5	157,2	112	24	13
17	2	53,7	158,4	133	37	11
17	2	72,3	178	112	25	32
17	2	50,6	155,3	153	37	12

17	2	55	154,8	109	30	13
17	2	81,5	162,3	100	32	11
17	2	94	157,9	122	36	5
17	2	73,8	156,9	136	24	26
17	2	60,5	154,3	100	22	27
17	2	63	152	120	29	2
17	2	46,4	156,4	114	38	1
17	2	67,5	151	108	21	3
17	2	65,7	152,2	169	40	29
17	2	80,3	165	103	21	7
17	2	62,3	156,8	130	29	10

20-m Shuttle ı Σ skinfolds
12,5
11,5
13,2
12,6
12,9
12,2
11,1
17,3
11,3
11,4
17
12,4
13,6
10,8
20,6
17,8
23,2
13,9
9
9,3
11,9
15,9
11
9
11,2
8,7
14,4
16,6
9
16,2
10,4
10,3
16,4
12,8
16,5
10,1
22,6
27,3
31,9
16,5
18,1
24,4
27,6
16
9,9
16,4
10,8
19,1
14,8

8,3
9,8
21,5
13,5
17,9
12,1
11,7
12,4
28,1
12,6
12,3
15,5
13,2
14,3
15,1
11,5
14,2
14,4
16,4
9,1
18
14,2
18,3
10,8
17,5
13,8
18,6
13,6
10,7
18,8
12,3
9,7
9,4
13,5
12,6
16,5
16,2
15,2
14,4
10,9
11,5
11,1
10,2
9,9
17,7
11,4
15,4
10,7
12,2
20,3

12,1
11
10,5
12,4
9,8
10,8
25,4
14,7
10,5
18
14
15,6
17,4
14,6
12,8
18
12,5
11,9
8,7
9,9
12,6
11,2
16,2
17,2
20
10,5
9,6
12,8
20,4
19,1
55,8
36,9
42,8
44,4
15,5
18,5
20,5
22,5
15,2
20,4
18,7
16,3
27
31,6
13,4
12,1
11,2
10
28,7
7,7

12
11,4
11,8
14,2
21,1
15
12,5
9,1
11,7
15,7
14,1
10,9
9,6
13,7
18,9
8,2
13,5
13,8
11,7
14,5
13,4
8,7
14,8
16
13,7
14,2
10,7
17,1
12,5
10,7
14,7
16,5
11,4
10,5
11
16,3
13
10,4
11,4
11,2
16
10,3
12,2
13,4
12,1
19,3
11,5
14,4
14,9
9,7

18,8
17,5
11,3
21,5
14,9
15,4
10,2
16,7
12
12,6
13,7
11,2
14,4
10,3
12,1
12,3
13,6
14,3
9,6
10
22,9
21,6
9,8
12,1
12,4
19,3
13,2
14,1
10,7
18,4
16
13,3
11,5
15,1
23,9
66,9
33,4
20,1
19,1
27,1
26,1
23,8
21,1
39,3
16,2
16,6
11,9
14,4
10,3
13,7

8,9
11,3
12,1
10,2
21,6
13
16,2
8,2
15,5
14
14,8
13,9
12,8
13,2
14,1
11,9
15,8
16,6
10,7
12,4
10
16,4
47,6
18,8
19,3
17,3
23,8
35
21,1
15,9
9,2
14,5
7,5
14
11,1
19,9
16,4
15,7
23,5
19,2
37,1
28,7
20,9
12,4
16,8
12,7
16,3
22,3
13,4
10

15,1
12
15,6
14,9
17,6
11,3
12,5
25,2
14
19,7
11
18
11,7
17,4
19,5
17,1
23,9
26,2
31,9
29,6
26,1
23
14,1
19,8
25
16,3
14,2
15,6
9,3
16,8
17,1
11,4
16,7
17,8
9,5
12,6
9,3
14
14,9
25,4
16,4
13
12,2
17,8
14,2
12,4
17
11
12
19,3

21
14,9
13,9
15,3
14,9
12,7
10,3
14,6
15,7
18,1
18,5
11,8
19,2
20
15,8
20,3
19,7
12,4
13,1
18,7
16,7
11,3
14,9
17
12,9
13,9
16,3
16,2
10,2
16,4
10,5
17,7
15,4
15,9
17,5
13,9
11,1
16,9
16,9
16,4
18,8
16,2
10,1
15
63
24,4
54,6
38,6
62,3
29,5

33,7
33,3
31,2
19,6
33,7
25
26,4
26,8
37,3
31,5
44,1
43,8
27,3
25,3
15,8
17,7
20,2
20,6
35,2
37,4
15
15,8
18,3
13,1
10,3
14,4
17,7
20,1
18
14,8
16,8
21
13,3
17,5
11,6
14,4
24
13,6
20
22
18,4
19,6
13
18,4
21,6
9,6
14,5
26,4
14,4
18,1

16,1
12,3
18
9,7
19
13,8
18,6
16,7
14,4
21,5
13,5
10,5
11,3
12,5
15,3
13,6
10,3
17,5
16,2
14,7
14,3
14,4
10,1
10,5
15,7
14,9
16,8
16,6
40,4
40,2
36,5
48,2
20,5
18,6
33,7
16,7
24,2
31
22,8
29,9
13,9
8,2
16,6
14,5
17
18,3
12,6
14,1
15,8
20,1

31,7
15,7
10,3
16,6
12,1
9,9
16,3
23,6
17,4
17,2
19,5
10,6
10,1
32,7
19,2
39,3
17,4
27,7
34,6
11,3
14,8
9,9
17,8
12,1
14,3
12,5
18,5
10,1
27,2
11,2
12,5
17,7
19,8
11,6
12,7
10,5
11,9
17,9
14,2
13,3
14
13,2
18,1
22,5
18,3
11,8
14,2
7,4
12,2
9,4

17,7
12
12
21
24
33,2
28,8
51,2
31,5
13,8
26
23,4
26,4
40,5
13,1
10,4
15,8
12,1
19,1
13,8
18
13,8
11
13,3
16,6
13,3
11,2
13,2
11,5
13,5
13,9
14,7
11,7
10,5
10,9
15,5
14,4
15,5
11,5
11,7
13,3
15,4
14,5
13,1
11,1
8,9
9,6
11,5
9,7
12

12,3
14,1
11,3
18,7
11
11,4
11,6
7,7
11,9
15,1
12,5
10,7
15,1
14,8
9,7
28
10,9
10,2
16,1
10,2
12,3
8,6
20,5
10,5
12,6
8,9
15,8
14
11,6
35,1
43,4
35,6
26
19,4
22,1
23,5
37,3
26
29
22,8
12
16
9,8
14,3
13,9
12,6
11,1
11,1
8,4
9,8

10,9
10,3
10,8
14,1
18
18,3
16,5
12,5
17,4
10
13,6
11,9
11
18,6
13,3
9,3
11,7
10,9
14,4
18,4
35
15,1
9,8
16,4
24,3
13,7
13
19
13,5
15,9
12
10,4
15,4
13,4
14
12
11,1
21
18,6
11,3
15,9
14,5
11,4
8
36,6
40,5
38,8
42,5
26
18,5

20,5
22,5
20,3
41,1
32,1
24,9
31
14,9
11
14,5
10,3
16,9
15
15,8
25,9
10,5
11,8
11,1
15,2
12,2
12,4
15,4
15,5
12,3
15,7
10
13,5
9,5
10,8
13,3
9,3
9,1
9,3
9,7
10,7
12
43,5
17
10,7
11,6
10,3
8,3
9,8
11,5
12,3
7,3
26,8
22,7
12,8
21,3

17,1
16,5
12,4
22,6
16,2
15,7
13,3
12,5
12,9
21,5
10,7
16,7
25
16
24
32,9
27,2
23,2
26
8
19,8
15,5
18,2
22,3
17,8
15,6
12,7
13
12,6
17
10,5
21,2
16,6
14,5
14,5
12,1
16,2
13,1
13,4
14,1
17,8
19,4
18,4
17,1
22,8
13,2
13,7
19,7
19
23,6

18,1
21,7
17,8
15,3
20
20,5
13
12,6
11,1
18,3
13,6
34
29,3
15
13,8
29,1
22,2
15,2
12,6
18,5
19,2
16,3
14,9
16,2
42
47,3
53
35
44,8
30
56,5
41,7
54,7
41,2
36,4
22,3
30,5
39
23,4
36,8
42,4
33,4
24,3
11,9
14,7
16,6
17,5
23,6
13,7
11,7

26,9
16,4
40,4
13,3
21,8
10,1
19,3
12
13,8
13,2
36,6
14,2
27,6
17,5
12,5
17,1
14,7
17,8
18,3
17,4
18,9
29,7
17,2
15,4
20,1
19,5
17,1
11
19
18,8
10,8
15,2
18
31
28,7
14,6
20
19
14,8
19,3
12,6
20,5
18,8
14,6
18,4
23,4
12,3
9,4
18,7
17,1

14,4
26
34,6
56,3
51
46
41,5
32,5
29,2
27,8
28
21,3
38,4
27,7
26
43
12,3
15
10,8
11
17
16,1
23,1
14,7
18,1
9,7
15,5
16,6
20,1
18,2
15,2
10,2
13
11,7
55,9
18,5
25,8
38,9
23
27,4
19
14,9
8
13,1
14,1
15
12
10,6
41,5
11,1

19,8
13,4
10
17,4
12,6
56,4
61,9
22,6
28,2
8,8
29,5
16,7
9
21,5
15,9
18,5
9,9
22,4
10,9
13
11,3
15,3
18,1
21
22,6
31,1
12,8
13,7
39,7
25,1
29,8
40
41
34,5
21,8
36,9
40,2
26,4
42
32,4
38
47,2
18,1
17,7
10,3
24,5
26
13,8
13
13,7

14,5
15,5
15
11,3
11,3
9,1
22,1
15,2
17,6
12,9
11,3
20,4
11
18,6
16
12,9
20
16
17,5
7,3
24,4
13,8
9,8
16,1
15,7
8,3
10,8
16,3
12,3
20,3
12,6
18,2
23,2
9,3
9,6
11
22,1
13,6
15,4
16,7
23,3
10,5
13,3
13,9
17,9
8,6
46,4
45,9
32
22

22,6
25,6
45,5
35,2
30
42,2
29
30,1
26
21,8
31,8
45,5
14,5
19,7
21,4
7,7
7,9
11,8
13,6
9,3
16,7
13,4
10,5
12
17,2
14,2
16,3
10,3
10,1
11,9
13,5
25,5
17,1
11,1
15,3
9,7
22,5
17,1
13,1
22,2
15
10,4
12,6
16,5
14,3
19,1
15,7
13,8
13,5
11,8

15,9
17,6
26,3
18,7
13,8
13,5
10,1
11,8
43,3
48
37,3
52,7
26
23,1
22,9
19,7
20,5
23,4
23,8
34,3
31,3
19,5
33
13,9
10,4
9,1
19,9
9,7
16,2
12
14,7
20
9,4
11,8
10,7
11,1
12,2
9,5
16,8
13
11,4
11,1
9,6
19
12
11,5
11,2
15,7
11,9
14,3

10,9
58
26,6
32,5
24,2
23,5
19,3
8,2
29
16,1
10,1
22,8
39
20
30
20
15,6
15,4
22,2
14,8
25,2
10,9
11,3
21
14,8
21,3
26,5
13,2
41
44
27,1
33,2
54,7
34,5
11,7
9,8
14,5
18,7
15,4
17,5
20,4
10,9
11,8
9,2
11,2
17,1
24
25,4
23,7
21,8

27
13
22,2
15,6
25
23,7
20,2
19,7
26,2
18,1
23,7
14,1
24
13
19,3
14
8,5
23,4
20
13
9,4
16,7
23,1
20,5
15,1
18,5
14,7
15,7
41,5
22,4
19,5
41,9
33,8
37,5
32,9
70,4
47,5
18,8
29,6
24
26,6
31,7
35,7
29
29,9
29,2
26,6
34,9
22,6
25,3

18,5
16,3
23,1
19,4
24,3
23,4
11,7
28,8
22
12,2
22,5
13,8
20,5
14,9
35,6
19
12
14,7
17,5
14,1
13
8,2
18,1
22,3
25,4
13,9
13
34
14,5
14,4
14,8
19,7
19,1
19,7
17,7
11,3
17,1
12,2
23
16,6
16,5
43,3
48,3
77,5
38,7
22,4
31,1
32,5
48,3
33,8

46,4
41,2
20,9
15,3
10,6
11,6
13
20,4
10,5
16,1
36,3
14,1
15,4
38,5
15,8
13,3
17,8
15,2
13,3
17,7
15,7
33,3
44,8
64
25,1
28,5
8,3
22
12
18
15,4
9,8
15,8
32,3
27,2
11,4
11,5
18
12,1
30,5
12,3
14,6
9
14,4
25,2
19,6
26,5
21,4
9,7
10

25,5
15,9
13,9
36
50,6
39,6
67,3
23,5
37,3
22
36,7
20,6
45
34,7
13,7
10,7
18,4
14,2
17,1
16,8
21
18,6
20,2
21,2
12,1
19,5
20,7
12,9
14
10,6
17,7
23,3
19,5
13,4
16
12
17,8
10,6
12,7
10,6
9,4
21
29,8
9,9
20,1
22,2
21,7
21,9
12,4
17,3

	11,1
	29
	8,9
	9,2
	12,8
	14,7
	13,7
	13,2
	17,8
	25,2
	18,2
	13,7
	7,6
	20,4
	7,3
	12
	9,8
	14,4
	12,6
	26,3
	8,6
	27,8
	13
	18,1
	21
	13,1
	7,8
	26,2
39	10,4
	20,5
	21,2
	14,7
	13,3
	7,4
	20,1
	30,2
	58,6
	44,5
	57,6
	33
	51
	23,6
	72
	64
	21,3
	22,8
	39,2
	31,3
	21,8
	36,5

34,5
34,2
25,7
34
40,1
20,5
9,5
11,6
19,6
34
26,5
9,2
15,4
29,3
13,2
35,8
13,8
23,3
15,9
17,1
17,7
26
12,4
11,5
17
18,4
19,8
18,6
10,5
8
13,4
18,3
32,7
20,3
13,1
13,4
31,7
13,1
18,9
16
14,5
10,8
23,9
20,8
15,7
15,4
10
11,7
17,6
16,7

11,3
20,1
13,5
23,3
12,3
16,3
17,8
10,2
12,4
24
19,2
17,5
21,9
12,9
17,3
47,8
61,3
74
53,6
64,5
53
45,7
37,5
44,5
30
23
40,4
27,5
20,1
51,6
12,3
13,6
12,9
16,7
12
16
13,6
9,5
11,2
18,1
9,9
11,9
19,3
8,9
22,5
10,4
75
26,4
30,8
11,7

12,1
20,5
12
56,6
54,2
45,2
25,5
39,2
13,4
18,3
23,7
17,4
17,3
17,4
27,5
23
21,4
15,2
12,2
11,4
25,3
18,2
20,4
22,9
15,7
36,5
24,7
11,4
21,3
11,4
13,8
17
17,3
15,1
22,3
22,5
28
13,3
24,2
11,1
24,2
23,2
13,2
15,7
23,5
12,1
42
61,1
34,1
32

39,8
34,8
40
10,7
10,5
14,9
11,7
27
13,8
10,7
21,4
22,9
13,8
22
28
16,5
13
16,9
17,6
17,4
32,3
15
10,1
14,7
16,2
18,4
37
17,8
21,6
11,3
16
18
17
21,3
24,1
18,1
27,6
14
19,6
13,7
11,7
27,8
18
18,6
23,6
14,2
24,4
22,2
63,9
51,4

	60,6
	44,5
	31,7
	40
	35,6
	37,8
	25,9
	40,6
	40
	28,1
	37,6
	39,5
	22,6
20	45,3
	19
	33,7
	13,5
	15,5
	19,7
	21,7
	14
	16,1
	14,1
	46
	29,7
	17,4
	24,3
	14
	13,4
	17
	20,8
	9,6
	23,7
	18,4
	16,6
	20,6
	24,3
	19,4
	17,7
	20,3
	19
	18,2
	35,5
	29
	25,4
	9,5
	18,8
	30,8
	31,1
	10,8

18
16,9
31,3
24,5
18,2
26
10,2
12
12,2
18,8
28,4
43
34,6
32,9
54,4
43
39,7
40,2
26,5
31,7
45,1
31,1
51,8
44,5
12
21,5
12,8
10,4
23
31
23,5
18,1
13
21,9
17
18,4
24,7
28,3
38
13,3
20,4
23
16,6
12,2
24,1
19,3
14,9
13,2
20
18

	17,8
	18,6
	26,7
	18
	14
	16,9
	12,7
	20,9
	28,1
	29,2
	21,2
	17,6
	31,2
	35,5
	48,2
	34
	25,5
	39,5
	24,7
	19,9
	12,5
14	21,3
	60,3
	35,4
	15,6
12	23,5
	9,1
	16,6
26	14,3
38	18,1
21	44,5
16	52,5
	28,1
	39,1
	22,5
	22
	13,9
	19,4
24	28,2
25	20
	9,4
	13,2
	27,7
	15,5
41	13,3
19	22,2
	19,7
29	32,3
34	10,3
46	10,3

20	19,2
	23
46	17,7
35	14,2
28	13,8
24	18
	26,8
33	18,2
	18,4
	15,3
21	10
	15,9
	48,4
	85
16	56
17	55,2
	43
	29,1
	39,5
	42,3
20	42,2
38	30,8
	34,8
20	53,2
37	13,7
	11,9
	26
	19
	9,3
	11,6
	12,4
	25,6
34	17,2
	13
	11
	29,2
42	9,2
	12,7
	17,2
39	12,1
	13,3
11	10,2
	9,2
	19,3
	24,2
34	17,8
	12,7
35	13,5
29	13,4
21	29,9

39	25,5
23	12,1
34	18,4
	10,9
	10,5
13	15,6
37	13,8
	14
	10,5
25	27,8
	18
27	10,4
	14,6
15	15,2
	33,5
	17
	11,7
25	10,7
	18,5
	11,8
18	22,8
22	30,8
17	19,8
45	15,6
34	15,4
16	27,7
30	25,4
41	11,2
16	16,2
22	11,6
21	16,3
46	20,3
24	14,1
24	18,4
46	13
53	10,4
56	11,9
	12
25	14,1
16	23,9
15	40,7
	75,1
9	58
17	41,8
	19,7
	23,7
	23,5
	39,5
	34,2
	40,4

	46,1
	35,5
	31,7
30	21,6
34	31,2
	13,9
	30,8
22	26,8
	39
	12,1
	11,7
	13,1
	15,6
	12,6
	19,4
	22,8
	12
	17,5
	11,6
	33,5
	14,9
	14,1
	14,8
	11,6
	23,5
	18,4
	15,6
34	15,3
45	11,5
	15
	6,9
	22,6
	16,6
14	14,3
	11,7
	10,7
33	14,9
16	13,1
	13,8
33	13,9
	16
	26,1
	18,9
20	28,5
14	14,8
	18,2
	13
24	22,2
23	16,6
34	17,2

40	10,4
	10
	8,9
14	10,7
34	15
37	19,8
21	20,4
39	12,8
	15,5
	15
	29,8
48	19,6
	11,4
	29,5
23	35,6
	54
	62,5
17	57,6
	59,7
	35,5
	33,7
	35,3
	24,3
14	39,7
	46,5
	41
20	25,2
11	28,5
32	20,1
	38,5
	45,5
31	23,1
	28,8
	19
	13,5
	16,5
25	10,7
	10,2
24	11,2
	13,6
22	21,6
23	11,8
41	11,2
	26,2
43	14,5
25	8,5
	9
21	30
	21,2
42	19,4

	61,8
	61,6
	40,5
	41,9
	28,5
39	24,8
	13,7
	20,6
	12,2
	31,7
15	20,7
	44,3
31	13,3
34	10,7
	13,9
45	11,6
15	18,4
42	19
	33,2
21	27,2
10	62,5
8	42,3
20	28,8
20	32,4
	24,4
	13,8
	30,1
	27,3
12	15,9
	20
28	26,8
25	15,7
26	35,5
29	21,5
29	9,3
	15,7
	13,4
20	15,3
35	17,7
	13,1
	14,2
26	26,3
	27,2
9	29,8
23	20,5
	16,5
17	37,4
13	28,7
15	29,3
26	23

	20,1
19	17,1
22	19,2
17	16,4
10	24,2
36	13,5
5	65
15	35,2
	36,5
23	26
15	38,3
	37,3
12	42,3
13	35
	40,8
	26,4
	23,5
	13,1
	22,3
	16
	20
	23,3
	25,9
	23
	31,6
	14,2
	31,8
	30,4
	28,9
	23,2
	20,1
18	20,2
25	14,6
	12,1
	17,5
	24,5
	17
	14,8
	14,1
9	12,2
	35,2
22	15,3
20	23,8
	18,6
	18,1
25	12,9
31	21,7
	18,1
	39,6
	30,3

	15,8
	28,2
24	18,1
	23,4
	21,5
21	16
	18,2
17	20,9
	10,5
	15,2
21	40,5
9	20,5
32	42,8
32	17,1
31	20,3
	25
32	17,6
23	15,1
	27
22	10,5
29	13,7
29	23,4
15	23,6
14	14,2
	27,8
17	34
15	32,8
14	54,1
12	20,4
44	11,6
	14,5
44	21,6
	19,7
21	29,5
	28,2
21	13
15	21,6
29	14,5
	21
	55,5
	56,5
	64,7
7	50,2
8	38,6
	69
	71,6
10	55,2
	42,7
	42,9
	42,7

	39,5
	50
27	45,2
21	34,8
23	25,2
16	51
14	38,5
17	36,5
18	28,8
	43
	19,6
	14,7
	25
	19,6
	22,8
	30,3
	12,5
	20,8
	23,4
	12,8
	26,6
	18,6
	20,2
	14,6
	34,2
	24
	24,2
	17,6
	40,9
	17,3
	20,7
	16,7
18	13,8
	20,9
14	29,5
	26
34	14,4
20	15,4
	19,8
	39,7
27	17
	18,5
21	22,9
12	22,1
13	19,1
	17,3
	24,1
44	21,3
11	17,2
29	15

32	11
13	19,8
12	22,7
15	17,5
30	16
	12,8
20	30,9
15	21,9
15	21,4
15	19
10	24,6
27	13,8
22	21,4
	31
	17,3
20	27
	25,8
23	14,4
12	20,3
14	34,2
	55,6
	53,1
	46,1
	31,5
	11,3
22	29,2
25	28,5
15	33
	40,6
17	43,9
18	27,9
13	49,3
13	28,2
	55,6
	17,5
	37,5
	14,8
	36,1
	21
	16,9
	13,9
	36,1
15	31,6
31	16,8
	15,5
	31
	14,1
	17,5
	15,3
28	13,6

29	24,3
	13,4
34	25,5
	34,5
	31,6
37	13,8
	17,4
12	15,7
22	18,2
15	11,4
23	15,1
32	12,9
	15,4
38	22,1
	21,4
	13,7
	14,4
23	18,5
23	15,9
	51,6
6	67,1
	35,6
15	28,4
29	46,5
38	25,9
	32
26	53
15	24,8
	48,7
13	40,6
28	15,1
25	13,9
29	22,9
	9,8
	27,8
	35,2
17	21,6
26	12
25	15,1
27	17
59	13,4
46	9,9
	68,3
30	42,8
15	53,8
23	19,1
40	17
	18
37	17,9
16	11,7

53	24,7
30	16,7
21	16,1
21	17,7
	13,8
45	12,2
15	15,9
56	20,4
39	15,1
26	25,7
52	16,5
17	33,3
39	20,4
33	10
16	31,7
13	13,3
16	19,6
19	17
14	56,3
11	62
10	50,1
8	30
10	48,7
23	30,7
29	20,1
17	28,5
15	21,4
23	22,2
48	21,8
35	23,1
56	14
23	17,7
31	10
42	20,5
	20,2
60	12,5
19	11,5
34	14,4
16	18
35	19,7
15	11,1
31	15,7
14	21,5
25	28,7
32	14,7
	8,8
23	11,5
19	18,5
41	7,9
	12,7

52	11,2
52	17,6
50	11,4
49	22
20	13,9
11	14,5
30	17,4
17	29,4
21	20,4
37	34,6
52	22,7
17	13,4
31	23,5
18	11,6
14	28,1
21	14,9
22	22,6
15	23,3
53	11,3
52	9,6
23	20,6
27	12,8
27	17,1
29	32,7
16	10,2
53	12,5
25	11,1
9	14
62	13,4
36	13,3
8	43,2
30	46,6
10	42
13	46,8
16	38
15	36,4
26	40,6
22	55,5
24	45,9
11	29,6
26	45,5
16	48,5
11	44
20	45,5
22	38,9
28	34,1
41	11,2
26	37,5
24	23,4
	48,5

11	32,9
47	29,1
46	18,4
13	15,5
	12
41	9,5
17	56
28	15,4
30	21,5
44	10,2
	24,4
	13,5
40	23,6
25	15,6
	14,6
56	12
	17,5
25	12,3
17	32,8
30	19,2
	10,1
47	11,4
43	15,6
31	11,7
30	9,6
16	20,1
28	13,2
39	13
46	15,3
56	12,4
46	13,3
56	12,7
	61,7
19	12,6
24	13,4
37	21,2
60	13,4
51	11,7
41	14,3
57	13,6
44	17,3
22	14,5
44	16
53	9,6
16	11,8
34	10,1
40	12
38	37,6
43	12,8
41	15,3

24	12,6
33	14,7
73	11,4
36	12,4
16	19,7
72	12,1
38	22,8
	14,4
20	12,2
53	9
11	68,2
17	62,8
19	58,3
	67
33	50,5
23	40,3
43	35,9
	40,3
17	51
15	44,6
26	40,5
18	42,1
35	42
	36,5
24	27,7
30	24,4
33	58,5
27	48,4
6	29
	12,5
52	11
34	25,6
34	19,7
15	17,8
52	14,7
30	17,5
	13,9
	12,3
	28
37	16,1
32	16
	11,9
	13,2
35	14,8
	13,4
56	13,4
	13,5
19	13,8
30	8,6
40	9,4

30	15,2
16	12,5
22	14,1
60	13,8
50	15,9
20	25,7
21	12
59	26,5
27	23,5
21	13,7
17	20,8
31	11,5
30	12,2
60	26
23	51
	56,5
23	60,6
33	38
8	40,3
49	37,8
16	45
24	39,1
	23,7
11	14,5
	16,8
14	15,6
38	32,3
28	10,8
15	44
6	11
20	14,4
20	15,8
9	27,5
26	20,3
28	21,6
22	15
30	22,1
14	34,5
14	37,1
8	19,7
37	16,7
9	37,2
25	14,6
28	28,7
28	26
	42,6
23	19,3
25	22,1
16	20,5
19	19,1

23	21,8
17	16,8
17	30,6
10	21,3
22	25,9
16	21,4
19	16,4
	13,4
46	6,4
12	30,4
7	23,1
24	15,2
10	11,8
17	26,7
23	14
46	11,1
29	17,6
7	20
6	19,6
23	11
26	22,4
30	32,7
	16,3
23	16
15	58,3
20	40,4
15	33,6
20	45,1
12	40
35	35,9
14	53
6	66,5
20	44,5
24	45,3
25	14,7
9	16,3
21	22,4
	14,8
	23
26	28,7
11	20,3
21	33,6
13	14,1
15	15,4
17	35,4
25	28,2
26	16
43	29,2
17	9,7
22	22,9

29	15,4
23	11,4
15	15,2
20	17,8
30	19,8
41	14,1
37	31,1
17	16,2
21	22,5
20	21,1
26	20,2
29	27,6
26	17,3
12	22,6
30	18
22	9,4
	17,5
26	33
31	29,8
14	16,2
29	20,5
19	24,2
48	11,1
19	17,4
17	14,9
23	27,7
25	18,9
41	22,1
32	22,1
27	12,2
11	26,9
31	31,8
20	30,5
24	20
24	15,2
18	50,3
15	20,8
35	24,8
17	12
15	12,9
	18
37	24,5
21	20,2
22	36,5
25	21
12	21,3
31	21,3
24	18,2
21	11
15	25,9

22	32,7
19	23,5
29	22,4
22	13,2
15	17,5
28	20,4
26	36,4
10	48,1
8	61
23	44,5
12	71
7	66,1
30	40,3
23	56
7	63,2
20	42,3
17	36,9
19	49
11	36,3
15	58,8
15	28,6
30	53,8
31	31,2
	37,5
13	46
23	56
31	27,7
20	32,9
14	41,6
8	45,1
14	45,4
11	48,3
16	51,9
12	42,7
	12,5
17	32
13	22,4
17	26,1
19	23,2
12	28,7
17	15
19	17,2
	11,9
34	23,7
26	19,4
15	19,8
25	19,3
52	18,9
20	27,2
11	15,7

16	19,2
21	19,8
22	13,8
31	16,2
10	40
17	24,9
19	20,9
23	20,6
48	22,6
27	13,9
23	16,9
21	18,6
21	31
21	16,7
22	61,2
19	36,9
46	19,1
11	19,9
17	13,4
29	15,4
11	22,9
21	32,8
	15,8
17	23,3
20	17,4
20	22
18	25,3
19	13,9
22	23,6
	33,5
19	30,3
25	16,2
25	17,4
33	23,8
9	36,8
20	13,6
9	25,9
15	17,9
	11,7
36	20,9
40	14,7
20	43,2
10	27,7
23	16,9
26	20
43	13,4
42	20
41	23,1
36	15
25	16,2

26	15,1
34	15,3
35	19
19	30
11	26
13	39,6
32	24
27	22
21	27,6
8	17,7
24	28,4
20	10,8
27	28,5
22	35,7
20	23,4
33	14,5
28	16,7
13	72,5
13	64
14	52,8
12	79,5
17	63,4
17	48,9
10	88,6
9	57,1
17	35,1
30	30
8	34,7
26	36,1
9	68
14	31,1
17	41,5
9	36
17	43,9
5	52,6
17	36,6
10	44,2
27	37,4
11	49,8
19	38,3
15	29
8	43,4
10	39,3
	48,3
14	39,7
14	29,4
18	16,8
23	19,3
25	26
	29,7

38	13,6
	20,8
36	24,2
19	29,8
34	11,7
27	9,7
18	20,7
6	25,1
33	14,4
27	20,2
26	16,5
28	16,9
13	19,3
26	16,3
25	16,9
27	21,2
26	24,5
27	31,1
13	13,3
23	11,7
	14,3
61	13,1
55	17,8
15	21,3
31	36,5
8	26,3
31	26,8
19	31,7
30	23,3
24	37,1
22	20,3
27	20
28	25
7	16,4
24	19,3
	15,5
23	15,7
	64,5
23	33
24	27,1
21	45,3
11	41
18	31,8
17	33,7
11	33,9
14	42,9
22	29,5
19	58
29	51,3
17	21

24	23,1
19	14,3
26	23,9
59	14,6
46	17,6
25	16,2
36	13,7
16	28,7
61	12,3
34	29,2
62	10,7
26	64,8
30	70
	57,5
31	43,3
27	30
63	14,5
15	20,5
39	17,1
30	25,4
53	18,6
34	27,2
33	22,9
	25,1
23	13,9
18	21,6
15	21,4
20	19,3
74	9,5
15	48
36	19,1
30	14
31	12,1
19	45,3
30	21,5
28	27,3
35	17,6
61	16,3
35	16,2
39	17,7
60	22,5
46	14,5
17	35,2
41	41,3
35	14,3
34	14,4
16	65,5
33	49,5
23	56,6
42	53,3

23	30,1
18	36,3
12	56,5
47	22,2
62	44,2
31	38,5
45	11,5
32	9,6
31	10,1
33	16,2
23	12,1
51	14,2
52	18,8
9	13,8
20	16,7
36	10,1
22	16,6
32	28
39	10,1
60	16,3
30	21,2
59	9,1
16	42,7
35	13,7
15	17,9
61	14,6
17	18,9
15	11,4
21	22,9
73	19,8
52	14,8
13	25,8
42	13
48	17
44	15,6
35	15
64	17,2
23	21,5
26	19,5
67	9,8
38	15
57	17,8
23	15,4
36	8,6
36	30,9
14	23,2
43	21,2
39	15,4
60	13,9
18	16,2

33	13,1
26	16,6
49	14,7
49	14
53	17,4
34	25
35	27,5
26	12,8
70	11,7
22	18,4
62	18,1
23	12,7
40	14,3
48	11,5
14	16
44	21,1
45	19,1
51	16,6
35	7,9
27	27,7
37	17
18	19
54	16,1
53	17,5
34	13,2
41	13,2
30	14,7
37	19
38	18,2
44	16,4
23	17,4
46	13,7
	8,5
35	12
54	17
49	13,4
40	11,9
18	23,6
23	38,6
18	62,5
20	49,2
34	42
24	52,9
35	46,5
39	24,2
31	52,5
20	32,5
23	39,7
15	47
28	63,5

19	45
15	43
26	30,5
5	49,3
9	41
46	44,7
42	32,5
22	44,3
21	35,3
16	48,2
14	40,5
35	23,3
22	21,9
26	16,1
51	18,3
42	33,7
20	15,9
45	16,5
24	39,8
40	25,2
25	16,2
40	12,9
25	18,6
45	15,1
36	14,9
55	14,5
41	23,3
44	11,1
	10,3
23	20,3
63	10,2
61	16,6
27	21,7
93	11,5
11	26,2
29	16,3
17	74,6
56	21,5
25	24,3
29	12,1
52	15,7
22	20,9
48	13,9
24	16,8
38	11,5
45	9,1
30	21,6
28	10,1
34	30,4
24	24,3

23	16,9
62	21,1
23	20,9
24	30,5
53	22,5
43	10,7
41	21,5
43	13,5
46	14
53	13,2
20	19,4
53	9,6
30	22,5
23	11,8
25	14,9
22	20,7
54	11,9
	42,8
56	9,6
25	17
42	14,8
19	26,6
32	13,2
19	48,5
25	65
40	45,9
13	59,3
11	68
35	75,1
34	56,7
61	40,7
40	39,8
17	37,9
17	48,5
16	36,2
46	33
41	35,6
24	46,8
40	27
30	39,4
21	40,4
17	56,4
16	47,2
13	39,2
20	30,2
29	35,4
31	30,9
10	34,5
20	62,7
24	16,8

46	12,4
20	23,5
35	12,7
39	12,5
27	10,3
57	10,2
35	18,2
46	12,8
21	13,7
14	21
32	14,6
49	16,9
	14,7
12	26,9
42	14,2
31	20,5
46	12,9
20	16,3
31	19
37	11,4
	13
32	12,3
46	10,2
32	23,6
31	11,3
31	15,2
67	18,4
37	12,9
15	12,9
24	11,1
44	19,2
78	11,4
46	14,8
55	18,4
23	54,2
19	53,5
20	41,9
34	24,8
9	62,4
15	61
59	21,6
17	38,5
45	11,4
11	20,3
37	14
23	11,7
25	13,1
78	19,8
16	43,5
16	49,5

27	18,7
20	15,3
17	21,1
20	38,3
4	19,6
42	22,1
34	22
41	20,5
13	13,9
24	15,4
10	28,5
25	22
16	17,6
44	23,3
26	24,8
14	27
23	25,6
30	24,6
17	24,4
27	21,8
44	21,6
24	26,6
33	22,1
23	17,8
18	16,2
18	23,5
31	19,5
46	17,5
27	12,1
34	31
19	11,4
16	17
33	13,7
30	17,6
23	19,6
23	33,4
23	66
15	51
9	47
21	52,4
11	42,7
24	24
32	38,7
16	40,3
19	17,2
24	15
12	39,6
34	22,4
21	18,7
23	18,6

17	16
21	19,1
30	46,4
14	16,7
22	15,3
17	27,2
23	23,8
32	24,8
28	19,2
6	14,4
17	12,4
16	37,6
20	34
18	26,7
23	18,2
10	28,6
46	23,4
18	16,3
15	18,2
28	32,2
25	22,4
14	21,9
19	19
21	27
24	24,7
6	24,1
36	12,2
29	17,3
27	18,5
34	20,6
18	17,9
18	25,2
19	35,7
35	12
7	39,7
20	27,8
17	16,1
21	19,1
25	13,7
7	13,2
21	44,3
54	27,4
40	19
20	20,8
32	16,4
22	21
12	52,6
17	14,3
15	15,8
23	35

13	20,8
23	13,9
24	21,4
14	26,1
24	17,6
30	18,5
32	26,3
41	23,8
12	13,3
53	20,2
20	13,8
44	11,5
15	28,9
33	18
25	13,7
15	17,6
17	18,7
39	13,7
20	17,8
6	19,9
39	21,1
11	19,9
6	23,1
29	29,2
11	49,3
14	53,8
16	31
17	56,2
15	41,1
17	51,3
20	34,1
30	34,5
10	25,9
	32,6
20	35
33	41,5
23	41,4
32	22,6
10	45,6
39	54,6
29	42
15	65,8
22	24,5
13	50,1
35	19,7
35	12,2
26	25,1
15	24,5
24	16,7
30	13

30	14,5
5	28,4
32	11
38	21,7
24	18,2
24	23,7
39	15,3
28	19,4
32	16,9
28	51,6
6	16,1
26	15,1
10	23,9
8	20
37	27,1
8	30,9
3	27,9
48	15,9
28	29,7
27	21,3
42	15,1
17	15,1
21	38,2
20	18
24	26,8
21	22,4
60	21,5
17	28,2
22	22,8
24	17,4
12	21
31	26,8
33	20,1
13	32,5
36	16
23	17,3
26	24,7
29	24,9
49	19,7
16	16
34	20,3
28	14,2
15	17,7
24	15
60	20,5
24	17,1
42	17,2
	12,1
25	28,3
27	14

9	31
19	21,6
9	28,2
12	20,1
38	18,3
44	17,9
36	16,7
26	35,1
27	29,9
27	44,4
29	15,3
33	19,4
41	13,9
12	34,1
26	24,7
37	19,6
10	18,4
15	15,9
23	21,9
41	12,8
13	30,6
37	16,6
33	24
32	25,8
35	18,1
8	15,7
24	16,2
33	11,2
29	15,2
19	68,6
19	75
	54,5
17	53
21	45,5
17	24,2
12	33,5
16	39,7
20	32,8
23	26,5
20	48,1
12	49,4
18	51,3
34	28,2
25	38
8	42,6
21	42,5
22	15,6
17	27,3
31	16,7
15	31,2

27	17
40	21,2
16	18,3
20	16,4
15	30,3
12	34,9
8	21,5
49	25,7
	25
25	11,8
12	28,1
20	18,4
25	9,6
31	29,4
13	25,3
	20
44	25,9
29	39,8
31	14,6
46	21,8
20	16,1
21	21,8
18	33,7
34	21,7
37	16,7
16	20,6
12	20,9
20	17,8
16	35,3
25	20,8
45	22,5
29	21,5
22	22,8
23	22,3
33	26,7
22	26,7
24	16,7
23	43,8
13	13,8
23	15,5
12	11,8
13	26,2
25	20,7
27	27,4
17	25
44	73,7
13	49,5
12	59
14	34,8
17	40,7

19	58,6
13	55,3
31	23,8
40	10,5
20	12,1
29	36
34	22,8
26	19,7
12	31,7
20	39,2
11	45,2
20	20,5
35	20,9
42	13,5
25	30,4
36	12,3
57	20,2
44	27,3
34	8
69	14,4
16	33,7
10	29,6
9	39,5
13	50,6
21	53,5
76	15
24	26,4
39	13,8
35	18
58	15
25	16,9
66	14,2
54	9,7
49	17,3
45	19,5
81	11,2
44	12,4
35	21,5
10	26,9
44	14,8
35	12,7
36	20,9
35	19,8
56	23
23	20,7
34	13,3
53	17,1
49	13,6
49	26,5
11	15,6

59	12,8
47	13,7
70	12,1
57	44,5
9	59,9
47	44,9
23	28,6
39	34,2
15	55
16	27,1
23	20,1
24	39,3
35	25,5
20	24
24	13,5
24	41,5
67	14,3
47	13,5
53	12,2
25	12,4
52	38,4
53	23,1
62	12,9
31	21,4
47	32,9
40	10,2
23	23
47	13,8
43	16,9
25	18,2
96	15,9
68	12,7
68	12,7
52	9,6
53	15,1
54	17,6
89	11
29	16,1
21	27,1
25	16,8
23	13,8
68	17,1
6	31,7
66	11
36	21,4
22	22,6
59	13,6
24	14,3
40	11
41	28,1

43	17,9
57	12,1
38	13,4
25	19,1
29	11
30	19,2
65	30,5
62	11,1
45	15,7
38	14,4
26	34,1
33	18,4
25	13,2
39	18,1
17	10,5
33	15,4
30	11
33	18,5
40	12,7
34	16,8
60	13,8
19	32,3
61	14,4
48	17,5
44	13,5
20	18,7
27	13,2
60	11,2
25	29,4
42	22,1
64	10
48	9,4
44	14
46	34,5
23	20,3
21	15
40	10,6
61	19,7
55	15
36	20,6
25	10,3
65	23,8
33	12
35	20,1
7	14,1
63	26,9
35	13,6
32	13,6
37	14,4
46	14,4

47	17,4
38	24
27	12,8
21	12,4
30	23,4
86	13,4
53	11,2
36	23
22	12,8
23	12
95	11,6
16	63,4
26	54,2
26	59,1
17	58
20	52,3
	49,5
7	54,5
73	17,1
23	43,5
26	28,2
45	25,8
18	31,5
37	19,9
15	34
23	38,5
45	18,1
23	24,2
28	32,9
30	38,5
42	73,8
13	50,3
26	28,9
71	51,6
9	52,5
17	47,1
29	19,4
55	41,7
31	46,5
53	12,2
64	18,7
46	12,1
35	19,9
17	40
37	28,7
13	21,3
63	21,7
47	14,6
64	16,6
37	12,9

17	11,7
	18,1
60	16,7
49	16
54	26,7
30	17,2
26	15,7
61	11,9
21	15
55	11
14	13,1
36	19
57	13,2
19	19,9
49	17,6
45	16,6
41	14
28	20,1
76	15,3
47	15,1
54	16,9
54	10,5
32	12,2
40	10,4
51	20,4
30	33,7
44	12,8
26	13
60	14,6
21	41,7
39	19
30	21,3
63	12,4
61	11,8
5	45,8
14	13,7
18	12,9
28	24,5
24	14,5
49	10,6
68	26,4
43	8
56	17,3
55	15
38	18,8
65	8,4
47	14,3
33	8
29	18,5
43	13,8

48	11,3
75	12,4
39	15,7
51	13,4
30	9
56	12,5
27	19
19	18,7
61	20,8
81	17,2
33	13,4
54	24
62	16,7
41	27,7
21	19,3
26	19,4
15	12,1
58	11,7
67	15,7
41	20,2
15	21,3
42	20,9
50	12,5
48	13,9
5	29,5
12	56,8
33	28,6
10	86,5
34	35,2
16	44,7
13	48,2
12	50,4
9	43,3
17	45,2
43	29,1
22	40,5
	37,2
20	41,1
18	26,2
17	30,8
76	23
26	26,8
78	16,1
15	41,5
64	20
56	20,1
31	37,6
76	22,7
46	22,2
22	13,2

56	18,3
59	13,8
15	10,8
12	10
39	12,4
52	14,3
36	25
25	14,9
29	21,2
46	33,2
34	16,5
30	17,9
43	16,9
42	13
70	13,7
38	12,4
36	11,9
34	13,2
42	13,6
62	11
19	13,9
30	16,1
22	20
30	12,3
57	17,5
61	22,3
17	24,7
41	24,3
25	12,8
49	15,4
49	11,3
23	13
35	18,6
42	18,2
64	17,9
29	19,8
27	17,8
43	12,4
34	19,5
28	17,3
69	15,1
17	48,1
19	65,3
16	19,2
29	70,5
33	43,6
29	26,5
35	25,1
38	14,4
35	33,1

25	32,3
27	19,2
59	17,9
22	34,9
41	16,3
25	24
57	19,7
29	17,6
51	12,6
26	17,4
64	14,5
40	11,8
22	39,7
45	35,1
20	9,8
17	16
24	30,3
19	34
20	20,1
16	18,3
51	40,5
17	40,6
26	15,2
18	34
29	30,8
21	20,9
21	20
19	17
13	30,5
19	36,6
17	19,3
38	28,4
35	15,8
17	22,5
46	25,8
40	27,7
26	30
16	17,5
16	25,5
12	15,8
37	23,3
11	30,6
17	27,5
20	21,7
35	23,8
30	21,1
24	22,8
21	30
6	37,5
13	57,4

12	31,7
16	27,6
12	55,1
23	36,6
40	28
19	75,1
14	15,7
21	14,5
34	18,8
35	13,8
29	16,9
25	21,8
10	19,3
61	17,6
35	17,4
22	21,1
30	15,1
12	16
17	21,6
35	20,9
23	9,8
31	17,8
19	19,8
31	28,5
11	14,5
21	13,3
23	19,3
44	42,5
9	26,9
17	39,3
17	41
21	32,9
19	21,5
14	16,8
26	18,9
14	25,8
10	27,7
25	21,5
31	31
35	28,7
23	32,1
19	22,3
17	19,8
25	24,6
14	22,6
4	19,4
24	14,2
17	35,5
12	16,3
41	28,8

24	37,5
23	35,4
24	31
24	52,9
26	19
15	24,2
29	32,6
16	43,5
19	13
18	37,3
10	19,4
39	19,3
12	18,3
26	15,9
24	26,7
34	26,9
12	28,4
23	19,3
16	17,9
22	31,5
10	23,2
35	21,1
17	25,3
16	23,4
16	35,5
31	29,3
24	11,6
16	43,6
15	30,2
23	25,7
27	21,3
21	15,6
10	23,7
17	42,2
36	54,8
11	31,4
14	26,8
22	34
11	42
10	22,8
18	39,1
24	28,9
25	27,6
16	19,7
30	26,8
35	11,9
16	17,2
26	24,5
34	22,5
21	31,7

21	21,3
11	24,8
25	15,2
25	27,5
30	34,6
10	37,6
22	19,2
17	24,6
15	57,2
18	48,9
6	59,5
8	79,6
14	54
10	62
29	41,9
18	35,3
40	62,9
13	48,7
19	49,7
24	52,9
15	51,5
41	20,3
25	54,5
21	40,7
17	45,2
34	49,7
14	43,7
15	46,1
29	34,6
39	18,1
30	18,2
25	44,5
12	28,8
14	18
20	15,7
54	17,3
19	27,2
19	19,1
7	29,7
21	22,6
10	28,7
39	22,7
6	27,3
25	21
7	24,3
32	19
7	28,8
15	26,3
16	30
23	22,4

24	22,9
33	16,8
16	39
33	20,3
21	37,3
10	28,2
14	16,7
27	22,6
16	21,4
13	42,5
25	15
33	25,4
23	32,3
27	22,9
14	24,5
17	11,4
40	15,9
19	27,5
22	24,3
17	28,9
44	20,1
22	14,9
28	19,4
33	14,7
23	30,6
	45,5
21	18,9
26	22,7
23	14,2
38	11,1
7	19,7
17	31
24	20
26	23
12	44
34	14
5	25,7
24	17,8
21	20,1
16	19,5
30	33,2
14	10,5
25	23,1
23	12,5
12	39,2
14	15
18	36,4
15	21,4
30	26,2
17	19,5

10	40,8
16	18,7
54	11
27	19,6
11	31,7
18	25,2
26	19,5
19	35,7
	48,9
14	26
15	35,5
38	16,5
16	29,7
26	30,4
20	32,8
28	23,8
25	11,1
23	21
12	28,4
21	32,4
7	34,4
19	21,3
14	56,4
27	53,6
7	98
10	71,5
4	48,6
15	50,6
14	35,8
39	20,1
23	37
20	48,2
29	45,3
17	47,2
32	49,6
25	48,9
10	51,6
27	37,7
7	36,7
32	39,2
5	48,4
25	45,3
16	38,5
35	36,7
18	36,2
14	44,9
24	62,2
27	41
12	25,8
50	26

14	24,7
35	22,7
8	33,6
9	28,1
20	17,7
17	34,6
22	16,3
39	15,4
41	23,5
26	24,9
28	35,3
18	20,3
44	18,4
37	12
24	15,3
26	16,9
21	24
12	20
16	26,9
28	24,2
19	23,7
20	31,8
42	15,1
17	18,7
26	23,5
29	23,7
19	38,2
8	32
27	33,8
19	17,7
42	24,4
33	20,2
22	20
15	23,2
63	16,2
24	55,3
16	46,5
27	78,7
19	38,6
15	43,4
14	46,8
21	37,9
15	52
16	38,8
17	19,1
34	25,9
10	26,2
23	15,7
17	11,9
12	28

9	42,8
8	36,2
25	13
57	18,6
31	11,9
30	14,5
59	14,2
53	13,6
58	18,8
24	26,8
68	19,4
60	13,6
20	13,4
54	23,8
41	41,1
50	43,5
64	21
23	14,4
46	26,7
58	11,3
30	22,4
53	26,1
31	18,5
69	10,1
33	16,4
42	18,8
45	12,9
62	17,5
21	19,3
35	15
36	29,1
30	14,4
57	14,2
65	15,4
64	15,1
52	21,1
23	15,7
63	14,3
21	17,8
47	18
35	18,6
60	11,7
44	14,3
25	16,1
37	10,9
46	14,7
68	11,1
51	12,7
49	12,8
21	12,6

20	13,9
10	59
24	51
23	58
24	48,1
26	68,3
8	34,7
19	43
43	53,4
30	46,5
34	12,2
44	12,8
40	13
45	14
63	21,4
39	13,8
44	10,1
56	13,6
40	17,3
47	12,7
18	12,6
44	17
77	13,2
27	11,5
17	12,5
52	11
50	16,7
43	11,1
64	10,2
45	18,5
5	20,2
73	19
48	14,4
54	28,2
53	28,6
37	16,1
61	8,7
70	19,2
69	12,4
64	18,7
40	13,4
53	13,5
54	11,4
28	14
33	13,5
42	13
54	10
57	13,7
54	17,7
38	14,6

62	8,2
52	20,1
37	12,3
67	8,6
40	13,3
29	21,1
8	13
62	12,9
56	13,3
49	18
38	15,7
66	12
40	23,4
82	23,1
25	16,2
32	14,3
43	25,1
49	13,3
29	19,7
81	15,7
28	12,9
46	14,5
48	18,5
72	10,2
49	18,7
74	18,4
70	13,5
35	10,8
35	17,1
22	10,3
63	11,2
46	13,9
35	22,8
31	12,7
55	17,7
71	9,6
58	15,7
37	16,9
64	15,7
69	14,4
29	13,3
45	25,4
84	9,2
43	15,9
52	17,7
26	10
48	13,6
33	13,5
73	14,7
20	21,3

43	17,4
64	13,9
26	12,9
53	26,8
71	15
14	54,3
28	61,5
36	48,4
23	51
14	49,8
26	56,4
26	22,2
36	22,7
56	25,5
57	25,4
26	33,4
32	22,3
12	45,6
21	45,3
14	30,6
41	23,5
43	19,7
44	42,9
33	32,3
64	41,1
29	12,5
21	12,7
22	19,6
62	12,6
47	13,5
	16
60	15,4
31	27,6
14	11,5
76	21,6
7	9,4
22	10,9
60	13,7
56	28,4
68	11,6
25	32,7
56	8
64	12,7
20	37,7
47	15,6
80	22
44	11,2
42	18
40	14,5
34	13,3

69	12
38	24,7
76	12,7
24	13
10	10,4
20	18,2
17	13,2
42	11,2
40	11,1
36	10,2
63	9,7
22	24,6
52	12,6
28	15,6
53	14,1
55	12,4
57	17,8
49	11,2
39	12,5
48	17,2
14	27,7
85	15,2
63	16,8
37	25,9
52	17,5
32	30,1
75	9,5
58	15,5
24	17,7
42	27,7
59	16,2
61	15,9
32	13,2
26	30,1
36	19,3
33	13
31	18,8
44	11,9
33	12,8
64	11
32	14,3
43	19,5
60	16,8
33	35,8
53	20,8
85	18,4
32	18,8
35	9,5
59	12,3
30	18,2

33	45,2
29	51,5
6	94
26	42,4
54	18,5
43	12,1
60	25,7
35	45,3
34	28,8
50	32,5
26	20,5
30	24,8
66	12,8
53	13,1
26	20,1
33	15
33	24
47	21,9
78	12,2
42	19,1
49	15,7
14	14,8
48	17,8
31	11,3
28	16,3
38	17,8
25	12,1
69	16,1
62	10,3
85	9,7
17	29,3
50	24,2
67	13,6
26	10,8
29	24,4
62	10,9
56	13,5
54	23
39	14,2
37	12,9
66	15
43	10
22	7,8
45	11,6
64	19,9
44	11,4
63	18,4
79	11,1
64	14,1
25	12

48	17,7
36	13
25	15,7
28	13,4
77	11,4
45	11,8
13	9,2
89	11,8
62	36,2
35	31,5
50	15,8
36	37
43	34,5
41	26,6
49	39,4
44	25,7
37	40,7
25	18,6
28	31,3
42	13,1
85	15,2
78	12,2
40	13,7
56	13
41	17,5
61	8,3
78	12
69	18
39	20,7
25	34,8
38	19,8
20	39,9
26	34
16	22,2
24	37
18	25,4
15	35,3
24	43,5
15	30
26	26,5
22	16
35	26,1
11	22,8
28	30,1
17	24,5
9	20,2
35	23,4
26	21,8
21	41,4
17	25,1

16	13
14	30,7
25	23,5
17	29,4
16	13
25	30,2
30	23,9
9	29,6
19	54
19	13,9
16	17,4
39	34
31	23,5
3	34,9
19	16,7
18	21,9
8	24,5
19	24,1
31	29,9
25	31,5
18	37,7
11	18,1
16	51,7
12	49,7
24	52,5
16	54,9
31	44
34	16
28	15,7
23	22,5
14	32,2
19	22,5
24	26,9
15	25
27	40
23	16,8
30	13,2
15	34,8
11	46,5
36	22,3
20	21,2
26	20,3
26	28,6
32	23,7
24	22,5
20	35,5
20	36
20	25,2
43	28,4
23	43,6

19	16,1
16	49
33	27,7
39	20,7
6	31
15	19,3
12	15,1
33	11,3
33	18,7
13	22,1
35	17,6
17	27,1
34	39,2
37	18
17	24,9
22	20,9
15	18,4
14	25,3
18	33,3
29	23,2
8	28
22	24,8
16	55,8
17	40,1
10	33
15	14
21	35,5
24	29,7
15	20,7
52	21,5
33	31,9
23	17
22	17,2
40	23,6
20	39,2
18	24,2
11	31
30	17,7
30	25,5
28	15,4
22	34,5
13	43,7
20	38,1
33	29,1
28	20,9
8	44,6
15	21,7
51	38,4
33	21,3
22	14,8

17	40,4
22	20
33	26,3
33	33
25	33,6
29	30,7
10	68
43	85
25	45,6
17	72,9
6	67
18	42,4
27	37,8
17	51,3
19	32,4
21	37,6
15	52,4
16	41,9
14	42
18	30,8
15	65,8
33	45,7
15	76
27	36
24	40,9
17	26,5
24	21,4
38	25,7
19	17,4
15	24,2
18	25,6
23	27,6
20	16,1
30	21,1
48	19,7
13	32,6
15	28,5
30	19
19	38,9
46	21,9
20	14,4
23	31,5
14	31
33	20
17	29,6
24	18,8
10	18,8
25	25,6
20	26,1
33	43,3

20	27
9	28,4
25	13,5
28	23,5
45	22
23	29,2
17	17,1
34	25,7
22	39,7
23	28,9
36	28
17	27,2
52	34,2
28	29
15	31,8
25	26,2
25	34,2
18	38,6
30	34,5
10	17
12	15,6
17	32,5
26	26
12	27,5
14	23,7
34	34,1
24	29,2
23	23,7
34	12
12	37,1
10	28
34	32,7
17	27,8
47	20,5
31	21,7
22	23,5
44	29,3
23	10,8
11	16,8
12	18,5
23	28,7
25	24,4
26	22
25	35,4
15	42,5
30	24,7
25	38
14	22,6
35	28,3
18	20,5

18	16,2
15	19,6
34	52,2
25	68,5
21	86,3
19	77,8
7	44
19	37,4
19	61,4
11	42,3
7	68,6
23	47,7
15	51,3
14	45,9
17	48,6
10	64
12	42,5
31	50
10	52
30	48
10	36,6
8	44,3
12	49,5
26	34,2
10	50,5
29	48,5
23	52
16	60
8	50,3
24	16,5
31	14,7
15	13,7
21	30,1
39	17
10	26,5
22	23,4
29	22,4
16	33,4
14	20,1
16	42,6
21	24,3
22	27
23	20,9
36	33,6
26	21
10	20
36	15,4
12	33,1
17	37,1
12	17,4

43	20,6
38	23,1
20	28,9
36	18,7
9	28,4
16	27,3
14	29,4
10	23,5
30	26,6
24	22
12	26,7
16	19,8
16	38,1
20	18,2
21	21,1
19	19,2
13	32,2
20	32,5
24	20,1
	19,4
13	19,1
16	19
31	32
10	25,7
9	28
3	20,1
22	26,3
33	24,1
20	19
25	24,2
7	24,6
34	42,4
5	81
10	53,3
24	26
16	45,4
21	57,3
14	52
15	26,8
34	44
16	32,6
23	50,5
39	50,3
15	53,6
16	34,2
22	42,5
39	32,6
29	28,3
11	31,5
25	15,8

20	19
11	17,5
35	41,7
	33,7
15	19,5
33	18,4
24	26,1
20	24,9
8	27,8
37	49,3
30	18,9
62	14
46	17,7
84	13
22	12,1
54	14,2
33	18,7
60	16,6
56	22
18	104
17	67,2
63	46,8
40	43,8
30	11,3
50	9,2
29	15
55	14,6
16	14,9
42	16,4
74	16,7
40	12,2
48	13,8
36	11,5
73	10,5
37	18,9
58	17,4
18	18,2
46	18,6
49	11,4
54	11,4
31	17
17	20,6
36	17,9
49	9
34	14,1
58	12,5
46	29,2
48	19,2
19	58,2
79	19,5

48	26,6
18	30,9
35	40,9
43	21
35	36
51	13
89	13,7
72	11,8
49	19
63	12,4
40	25,8
52	8,4
56	14,1
39	11,8
18	9,3
54	16,4
50	18,8
34	35,9
30	6,8
53	13,7
46	14,5
81	12,7
38	11,4
40	16,2
67	20,5
46	16,9
51	19,5
32	15,4
21	14,2
56	11,3
77	13,4
52	18
50	18,2
70	10,2
42	10,2
40	13,7
54	20,3
42	11,4
48	13
48	16
69	14
28	23,7
86	10
42	16,5
47	14
35	24,2
56	10,5
36	18,6
35	10,8
41	12,7

23	15,4
35	11,7
39	21,1
37	14
60	9,9
60	13,7
44	14,4
49	18,7
41	17,6
62	13,8
59	14,9
86	17,2
68	12,6
54	19,9
35	13,5
27	15
68	13,1
46	11,2
23	45
18	48,6
43	65
42	28,7
34	20,7
22	37,6
49	22,9
22	39,8
61	16,9
46	33,3
55	22,5
30	43
26	34,8
63	19,8
37	16,4
64	9,9
68	15
17	31,6
31	18,5
63	16,6
44	10,8
82	12,5
28	18,5
76	20,2
46	11,8
36	11,9
42	14,9
68	13,9
63	11,4
33	14,8
64	10,8
76	24,2

17	20,3
53	27,7
54	12,4
83	11,1
29	14,5
27	11,7
54	14,6
42	15,3
14	18,8
53	13,1
83	13,2
63	9,7
35	17
68	18,7
68	12,2
26	16,9
46	16,1
40	21,7
49	15,2
64	16
54	8,9
30	17,8
54	22,4
54	11,7
10	12,5
46	13,2
35	13,6
23	22,8
32	20,1
46	21,7
39	27,9
43	15,3
67	12,6
40	12
54	13,8
37	12,5
71	16
37	18,7
15	18
61	14,1
49	12,2
77	13
47	17,2
47	10,2
81	10,7
41	13,9
31	10,1
36	17,6
34	24,5
76	31,3

67	37,7
25	66,3
35	21,7
22	52,1
52	28
34	40,5
93	15,1
23	32,1
44	14,5
65	10,8
37	21,7
57	11,9
54	8
10	14,3
36	15,7
39	15,8
20	21,2
41	12,9
87	15,9
83	14,4
89	11,1
88	15,9
48	16,5
52	13,6
52	11,3
26	17,2
53	10,7
74	16,8
34	10,7
40	11,9
12	15,9
43	12,3
47	17,3
33	11,1
7	16,5
18	11,6
43	13
52	10,9
87	12,9
54	11,2
64	11,6
72	6,8
48	13,1
58	52
46	29,8
65	17,7
36	54,1
33	15,5
11	42,4
39	12,8

3	18,7
12	38,7
48	30,2
20	32,8
31	38,5
24	31,5
22	31,8
15	43,4
24	25,8
53	20,1
20	33,3
39	26,1
15	7,5
25	19,1
3	23,1
21	24,2
29	18,3
21	35
27	26,5
26	28,7
20	50,5
41	25
17	17,8
18	20,4
15	17,8
10	23
10	31,3
18	17,6
13	20,7
15	32,9
25	36,7
17	40,7
9	48,9
10	63,2
18	50
10	59,7
23	44
15	45,1
22	30,5
16	32,3
35	31,3
33	16,2
14	19,6
41	14,8
18	22,7
26	25
23	28,7
30	23,9
23	19,2
19	18,2

24	21
19	25,3
33	19,7
25	16,5
8	18,6
23	33
12	22,3
17	33,1
42	38,5
26	23,3
47	38,3
9	23,2
25	30,8
12	32,7
42	28,8
22	16,1
29	27,8
20	53,1
10	35,3
21	21,2
26	19,3
28	16
10	30,3
46	26,1
15	25,8
32	43
23	20,6
27	28,5
24	22,3
10	25,3
14	25,5
15	26,5
36	38
20	24,2
15	43,2
24	29,1
23	35,6
41	35,6
20	29,6
42	20,9
16	19
23	15,5
16	26,8
15	34,1
29	30,9
17	33,3
31	23,2
8	36
40	26,9
33	20,5

12	24,5
20	31,3
28	20,5
23	30,3
25	25
20	22,7
16	25,4
42	31,6
14	38,5
30	26,5
10	35,3
30	16,2
20	49,3
10	23,3
13	28,8
15	17,3
30	16,9
10	22,3
29	22,1
15	52,9
22	23,7
29	50
19	55,9
10	72,5
40	67
23	45,3
9	68,1
42	42
8	48,5
19	52
23	55
22	54,4
15	58
16	18,8
10	40
15	35,6
12	21,3
24	36,1
16	21,8
30	23
10	20,1
28	27,4
9	21,7
17	44,2
22	31,7
26	37,5
24	32,1
25	32,5
11	24
15	29,8

61	19,5
26	17,7
12	27,7
24	30,8
23	24,6
10	38,9
33	26,6
15	39,5
7	47,7
35	33,9
30	24
22	17,4
17	24
39	24,5
33	20,1
17	22,1
17	17,1
26	23,8
15	27
19	35,2
12	45,8
17	15,9
21	34,1
4	22,3
33	22,6
26	18,5
14	32,9
15	21,9
26	26,4
34	29,7
15	20,2
26	29
10	43,3
21	26,2
42	19,8
20	32,2
35	19,4
34	26,2
17	35
25	22,4
11	37
20	29
15	33,7
30	41,4
35	20,5
17	29,9
10	28
10	52,6
33	27,1
16	66,2

27	21,4
28	24,4
12	32,3
26	37,5
20	45,4
43	34,5
27	27,5
34	33,2
19	27,8
19	26,8
33	14,6
15	35,4
23	34,9
10	26,3
24	26,3
18	23,6
14	25,7
25	30,3
23	19,4
8	82
17	60,4
40	73,5
10	58,1
34	55,5
15	43,3
20	71
39	34,4
10	72
20	43,2
11	54,5
19	44,3
12	38,3
19	17,5
28	24,5
15	15,8
28	18,8
28	22,8
36	22,5
21	32,2
16	37,5
23	26
20	21,3
35	35
16	44,7
29	29,4
42	15,2
7	27,1
35	25,9
10	42,1
26	31,7

16	26,8
30	34,5
34	22,6
13	35,4
10	15,6
22	21
46	21,2
15	27,9
28	30,7
25	51,5
13	44
24	27
17	14,6
10	17,5
15	35,6
15	38,8
19	46,5
30	20,9
8	16,8
27	30,5
15	22,1
43	25,4
29	16,9
14	29,3
10	16
12	84
17	55,1
20	19,2
75	28,6
40	14,9
57	17,3
18	14,9
56	16,9
39	18,2
51	16,3
28	16,5
35	38,8
51	24,5
37	12,2
23	25
41	18,3
40	11,2
41	15,3
54	14,2
80	14,3
54	12,9
64	13,5
55	10,7
64	14,8
77	17,2

69	14
56	21
67	12,6
38	23,9
37	18,7
36	25,6
44	21,4
70	20
49	11,6
36	16,8
44	11,7
56	15,9
72	40
54	32,2
36	19,7
55	39
57	17,6
62	9,8
21	14,2
50	13,8
14	18,5
42	16,5
16	14,3
54	35,5
71	14,8
36	28,1
44	10,4
53	12,7
23	14
39	14,4
26	15,7
72	14,7
52	12,1
44	9,7
40	13,7
49	18,1
81	19
62	10
85	13
60	22
93	21,3
42	19,2
22	17,4
84	12,5
58	13,6
63	16,7
40	19,6
31	22,4
38	16,9
75	18,8

41	25,4
63	19,8
52	23,3
44	11,8
46	23
70	23,3
42	14
65	11,3
90	9,5
44	23,4
58	21,2
81	14,4
26	48,3
26	56,6
74	54,2
22	60,1
26	54,3
20	65,7
31	62,7
102	12,6
90	10,9
46	24,6
46	32,7
40	20,6
25	46,2
54	17,3
35	14,2
28	19,6
63	12,2
15	10,9
40	11,3
91	20,1
47	11,2
36	12,8
28	18,7
49	7,8
42	12,2
41	19,3
62	12
40	15,3
76	15,8
89	15,7
42	24,8
54	14,8
64	21,4
57	12,7
41	12,9
90	12,4
89	11,1
63	12,5

39	12,5
49	13,8
49	15,5
65	9,6
29	23
41	12,9
69	14,9
54	12,7
47	13,8
38	15,7
75	24,3
66	17,7
35	49,8
22	55
8	58,7
7	52,3
71	15,4
19	31
36	25,5
65	14,5
31	36,7
47	21,1
69	11,5
73	10,2
26	16,8
52	11,1
50	18
68	13,2
51	21,5
52	10,9
77	18,2
7	14,9
46	17,7
51	18,8
41	29,5
29	16,7
54	11,3
50	12,6
64	9,4
57	30
56	21,3
65	20,1
97	10,2
80	27,2
10	24,9
40	17,5
22	20,5
13	43,4
54	24,1
7	48,5

21	32,5
12	25,3
33	24,5
4	24
35	25,6
14	26,6
29	39,7
45	36,5
24	32
24	34,4
24	20
34	38,5
33	20,3
17	19
34	22
25	27
26	44,3
24	47,9
15	30,4
24	35,4
15	61
10	53,6
18	51,5
26	59,3
19	36,2
12	32,8
47	24,4
24	17,1
14	24,3
17	21,3
12	41
12	18
16	20,3
19	25,7
10	25,6
26	23
36	14,1
16	12,8
24	20,4
31	43,8
15	23,6
41	29,6
31	30
19	35,5
31	29,7
15	17,1
15	23
29	45,2
30	21,2
30	34,3

24	48,3
17	21,4
9	25,6
35	38,6
34	32,7
21	15,7
8	18,4
1	50,3
47	40,8
13	28,9
25	18,4
17	50,4
42	27,7
35	24,5
21	25,8
19	30,4
22	32,6
19	19,5
18	52,1
17	27,6
25	21,7
15	31,4
26	29,3
25	20,7
13	37,3
11	30,9
34	17,4
15	20,3
8	26
23	31,5
36	27,8
34	24,3
15	33,7
9	21,9
9	73,9
12	37,4
34	51,2
21	48,6
23	43,4
14	34,6
7	40,5
30	63
15	51,7
17	36
23	63,9
33	19
50	26
34	22,7
18	23
18	16

11	17,3
13	32,7
14	25
20	29,8
11	11,8
33	15,4
28	25,8
19	23,7
15	16,4
33	32,2
37	24,3
14	32,1
61	12,6
33	25,3
13	23,3
10	20,4
8	43,2
15	24,7
23	27,4
17	21,4
35	23,6
14	34,4
17	37,2
19	18,9
21	40,6
32	28,9
17	21,8
22	19,7
54	18,5
28	13,6
26	26
33	32,9
34	25,2
25	16,7
34	30,6
12	26,4
23	26,4
14	24,2
9	38,3
32	26,3
35	94,3
22	39,9
8	46,5
17	45,6
25	43,4
14	44
21	36,5
10	45,3
16	43,6
28	39,1

23	21,8
25	33,8
19	33,2
18	13,7
25	24
14	35,4
50	20,1
21	19,2
24	20,7
18	56,9
37	33
30	24,9
34	28,3
24	25,8
20	31,3
24	20,7
11	29,4
15	33,5
18	34
17	38,3
25	33,3
32	29,7
27	23,5
46	17
57	16,8
25	21,2
14	23,1
15	17,2
16	30,5
20	30
5	56,5
8	31
15	21,8
23	60,9
24	22,7
13	47,3
13	53
13	42,4
29	18
21	31,3
12	16,1
17	25,4
44	86,3
90	12,9
26	16,7
64	15,5
82	14
52	12,5
69	13,5
35	8

20	12,4
37	20,5
49	22,8
59	12,1
62	21,7
102	10,8
68	21
84	10,7
53	15,7
85	17,7
37	17
37	47,5
31	44,6
21	33
35	29,8
44	27,7
59	22,9
27	13,8
33	41,2
68	25,6
60	10,5
58	17,3
85	14
70	10,9
65	15,1
32	14,5
20	32,5
16	19,1
65	13,1
69	11
59	12,4
71	9,6
59	12,9
56	12
56	15,4
58	23,5
73	13,4
88	18,8
56	22
44	18,7
36	23,8
65	11,5
62	23
32	19,8
73	15,1
75	17,8
68	14,3
41	15,5
81	14
36	28

63	27,3
22	15,4
71	13,4
43	12,7
54	16,7
113	16,7
75	16,3
46	19,4
65	13,9
54	19,9
61	60,5
63	30
65	41,2
61	25,5
77	22
25	33
53	45,5
77	24,4
20	33
54	14,9
62	10,1
65	14,4
75	13,7
54	17,4
59	12,8
46	22,5
84	16,2
88	16,3
63	15,9
63	10,9
43	19,1
71	19,3
66	24,7
75	12,4
49	20,8
17	41,2
46	17,2
26	14,1
76	11,7
84	15,2
107	25,4
34	11,6
80	18
65	18,9
88	11,7
69	15,5
63	13,7
45	9,2
30	62
48	50

63	52,9
64	32,6
47	32,3
75	47,5
62	19,7
64	24,1
73	11,1
47	12,4
42	12,7
44	16,5
55	12,7
116	15,1
86	17,8
66	15
38	14,4
75	24,1
59	13
40	36,2
89	24,9
67	19,6
47	21,8
37	10,8
46	30,2
34	38,4
23	32,3
18	36,1
25	27,6
42	44,3
39	29,9
25	21,7
24	15,9
44	25
21	23,8
30	44,9
10	48,1
34	44,4
37	47,4
20	22,2
24	18,1
15	18,8
24	34,8
10	32,2
60	13,6
26	19,8
44	27,8
30	24,7
31	19,5
27	15,6
14	36,6
12	32,7

43	32,2
13	30,5
9	22,6
34	22,4
23	22,7
14	25,7
15	28,1
22	19,8
26	38,7
19	24,5
13	31,7
19	27
52	14,1
46	32,7
21	21,3
36	27,4
16	32,7
23	42,3
23	25,4
17	38,3
25	21,5
21	39,8
11	14,4
16	24,8
47	17,7
15	16,3
37	36,4
21	45,5
13	36,8
17	22,6
20	25,2
16	33,5
16	37,7
19	27,7
25	22,7
10	43,2
19	93,8
15	41,7
18	60,7
23	64
16	42,8
10	26
21	40,7
73	15,1
23	23,5
13	54,6
34	24,3
15	31,1
12	23
46	50,5

24	46,2
15	41
17	33,7
31	43,6
11	26,7
20	29,8
16	16,2
17	18,8
31	17,4
10	32,2
15	28,2
21	28,9
37	37,5
11	22,5
34	24,1
25	35,5
23	38
13	39,6
10	18,6
34	54
9	22,8
26	33,7
23	21,3
8	27,5
18	20,4
15	43,7
31	22,5
16	31,1
16	28,6
22	27
31	35,2
20	19,4
43	53,7
30	48,3
22	45,5
40	45,5
24	42,5
23	55,3
15	78
21	23,5
22	26,5
18	27,3
16	30,9
19	28,6
12	42,3
16	37,4
45	24,5
23	21,2
49	40,7
23	18,1

13	31,9
19	57,1
15	56,1
14	51
27	45,1
22	12,6
27	21
20	39,1
33	35,6
11	63,3
13	27,4
